# Development of a Manufacturing Process toward the Convergent Synthesis of the COVID-19 Antiviral Ensitrelvir

**DOI:** 10.1021/acscentsci.2c01203

**Published:** 2023-04-07

**Authors:** Takahiro Kawajiri, Akihito Kijima, Atsuhiro Iimuro, Eisaku Ohashi, Katsuya Yamakawa, Kazushi Agura, Kengo Masuda, Kensuke Kouki, Koji Kasamatsu, Shuichi Yanagisawa, Sho Nakashima, Setsuya Shibahara, Takashi Toyota, Takafumi Higuchi, Takahiro Suto, Tadashi Oohara, Toshikatsu Maki, Naoto Sahara, Nobuaki Fukui, Hisayuki Wakamori, Hidaka Ikemoto, Hiroaki Murakami, Hiroyasu Ando, Masahiro Hosoya, Mizuki Sato, Yusuke Suzuki, Yuta Nakagawa, Yuto Unoh, Yoichi Hirano, Yoshitomo Nagasawa, Satoshi Goda, Takafumi Ohara, Takayuki Tsuritani

**Affiliations:** † API R&D Laboratory, Research Division, Shionogi & Co., Ltd., 1-3, Kuise Terajima 2-Chome, Amagasaki, Hyogo 660-0813, Japan; ‡ Laboratory for Medicinal Chemistry Research, Research Division, Shionogi & Co., Ltd., 1-1, Futaba-cho 3-Chome, Toyonaka, Osaka 561-0825, Japan

## Abstract

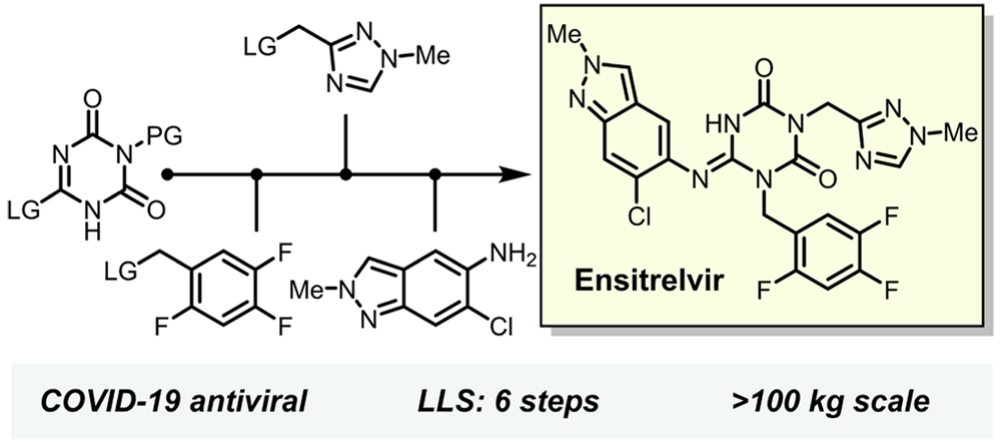

We describe the development of the practical manufacturing of Ensitrelvir, which was discovered as a SARS-CoV-2 antiviral candidate. Scalable synthetic methods of indazole, 1,2,4-triazole and 1,3,5-triazinone structures were established, and convergent couplings of these fragments enabled the development of a concise and efficient scale-up process to Ensitrelvir. In this process, introducing a *meta*-cresolyl moiety successfully enhanced the stability of intermediates. Compared to the initial route at the early research and development stage, the overall yield of the longest linear sequence (6 steps) was improved by approximately 7-fold. Furthermore, 9 out of the 12 isolated intermediates were crystallized directly from each reaction mixture without any extractive workup (direct isolation). This led to an efficient and environmentally friendly manufacturing process that minimizes waste of organic solvents, reagents, and processing time. This practical process for manufacturing Ensitrelvir should contribute to protection against COVID-19.

## Abstract 2

Ensitrelvir is a COVID-19 antiviral candidate with 3CL^pro^ inhibitory activity. Here we describe a practical synthetic process for Ensitrelvir by a convergent approach.

## Introduction

The coronavirus disease 2019 (COVID-19) pandemic caused by severe acute respiratory syndrome coronavirus 2 (SARS-CoV-2) continues to threaten human activity around the world.^1^ COVID-19 has been recognized as a palpable threat that demands efficacious treatment. Until now, two novel oral antiviral drugs, Molnupiravir^2−4^ (MK-4482 from Merck, formerly EIDD-2801 from Drug Innovations at Emory, LLC) and Nirmatrelvir^5^ (PF-07321332 from Pfizer), have been available and have helped reduce the risk of hospitalization or death.^6,7^ These breakthrough drugs based on small molecules can be supplied affordably, thus offering a remedy for a broad range of patients including those in developing countries.

**Figure 1:**
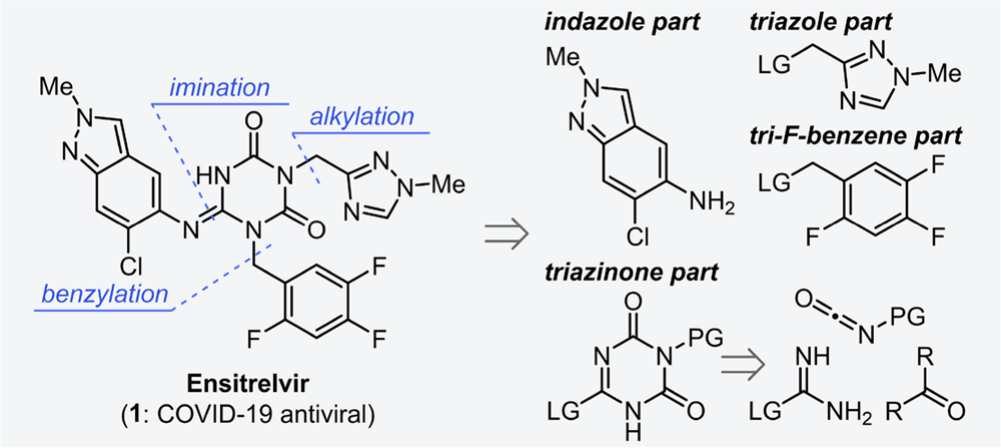
Chemical structure and synthetic strategy of Ensitrelvir **1**. LG: leaving group, PG: protecting group.

Here we introduce another clinical candidate for treating COVID-19, Ensitrelvir (a nonproprietary name for S-217622, **1**, Figure 1), which was discovered through a research collaboration between Shionogi & Co., Ltd. and Hokkaido university, as the first nonpeptidic, noncovalent 3CL^pro^ inhibitor.^8^**1** exhibited significant potent antiviral activity and high bioavailability arising from a favorable drug metabolism and pharmacokinetic (DMPK) profile as a once-daily oral medicine to treat COVID-19. Therefore, unlike Nirmatrelvir, **1** can be used alone, without a pharmacokinetic booster like Ritonavir. Treatment using **1** will offer a therapeutic option not only for severe or high-risk patients but also low-risk patients without underlying disease and should help to curb the spread of COVID-19. Recently, the therapeutic value of **1** against Omicron variants of SARS-CoV-2 has also been reported.^9−11^ Given the high demand for this medication, it is critical to develop a practical and sustainable manufacturing process of **1**.

**Scheme 1:**
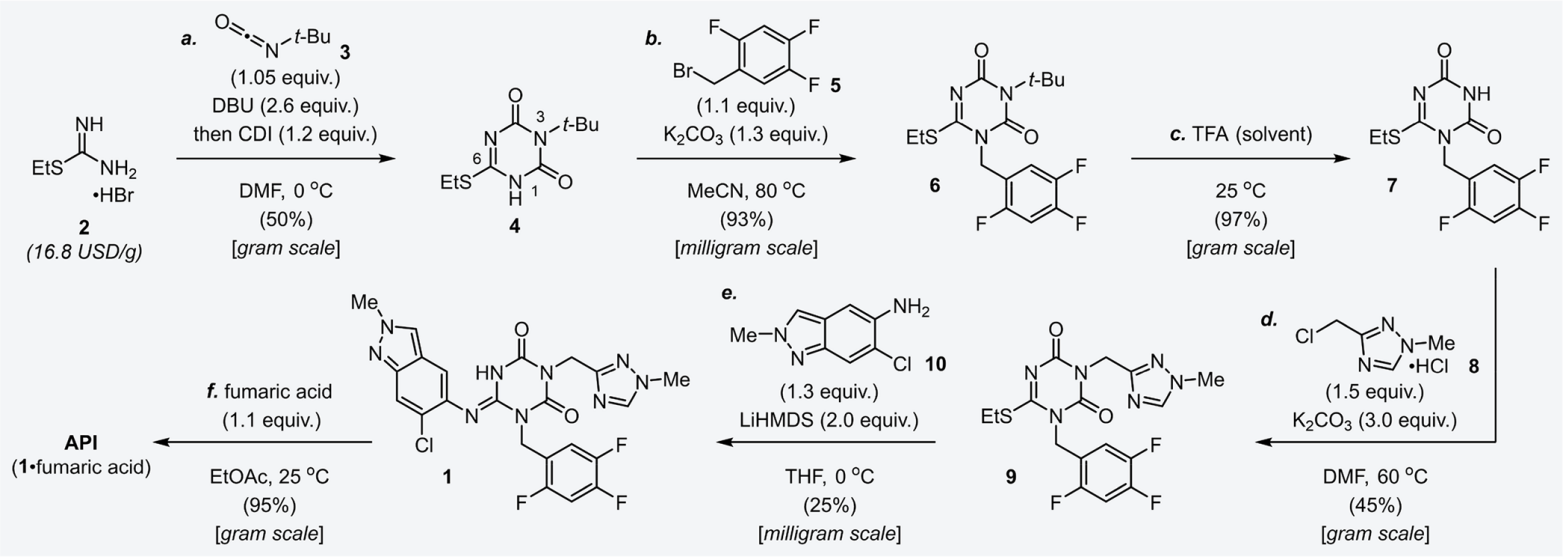
Synthetic Route to Ensitrelvir **1** at the Early Research and Development Stage

Structurally, Ensitrelvir **1** consists of a central 1,3,5-triazinone core structure and three (hetero)aromatic components including a nitrogen atom rich 1,2,4-triazole and an indazole motif. As shown in Figure 1, the strategy of sequential introduction of the three (hetero)aromatic compounds into the 1,3,5-triazinone core is an efficient and rational approach. From the viewpoint of manufacturing scale, the shown convergent synthetic strategy^12,13^ was also beneficial for reducing lead time for the supply of **1**. Namely, manufacturing time could be shortened by individual preparations of 1,3,5-triazinone, indazole, 1,2,4-triazole and 2,4,5-trifluorobenzene parts in parallel, followed by coupling of the fragments. The convergent approach was applied to the synthetic route, however, insufficient yield and the use of expensive compounds or cumbersome reagents were required at the early research and development stage, resulting in difficulty for large-scale synthesis (Scheme 1).

To overcome these problems, we established a practical and sustainable synthetic process of Ensitrelvir **1** by a convergent approach.

## Results and Discussion

### Outline of the Synthetic Route of Ensitrelvir at the Early Research and Development Stage

In the early research and development stage, the convergent synthetic strategy of Ensitrelvir **1** was also conducted for a structure–activity relationship (SAR) study (Scheme 1).^8^ According to the protocol reported previously,^14^ 1,3,5-triazinone derivative **4** was constructed from a commercially available material **2** in one step (step a). 2,4,5-Trifluorobenzyl bromide **5** as the first aromatic fragment was introduced into **4** in the presence of potassium carbonate (K_2_CO_3_), and the *N*-benzylated compound **6** was obtained in high yield (step b). The *tert*-butyl group on the 3-position of compound **6** was removed in trifluoroacetic acid (TFA) as solvent to afford the corresponding compound **7** (step c). Fragment coupling of **7** with 3-chloromethyl-1-methyl-1*H*-1,2,4-triazole **8** was performed under basic condition to provide **9** in moderate yield (45%, step d). Subsequently, **9** was coupled with 2*H*-2-methyl-5-amino-6-chloro-indazole **10**, and the target molecule **1** was obtained in 25% yield (step e). Concerns about inadequate bioavailability due to the low solubility of **1** necessitated the design of an appropriate salt or cocrystal of **1**. After comprehensive screening, including sodium salt, potassium salt, cocrystal with succinic acid, cocrystal with fumaric acid and anhydride form, the cocrystal of **1** with fumaric acid was selected as an active pharmaceutical ingredient (**API**) that exhibits excellent solubility and stability. Finally, **API** was crystallized from the ethyl acetate (EtOAc) solution in a 95% yield (step f). While the synthetic route at the early research and development stage was short and retrosynthetically reasonable, for sustainable manufacture of **1**, we needed to develop the process further. Specifically, the route at the early research and development stage gave an insufficient total yield for a steady supply of medications (4.8% from **2**). In addition, heteroaromatic compounds **8** and **10** were expensive, and their suppliers were limited. Therefore, an efficient synthetic method for these compounds was required. Furthermore, there were issues blocking scale-up synthesis, such as the evaporation of corrosive acid (TFA, step c), the noxious odors generated from thiol derivatives (steps a and e), and purification by silica gel column chromatography (steps b, d, and e).

### Preparation of the 1,2,4-Triazole Motif

**Scheme 2:**
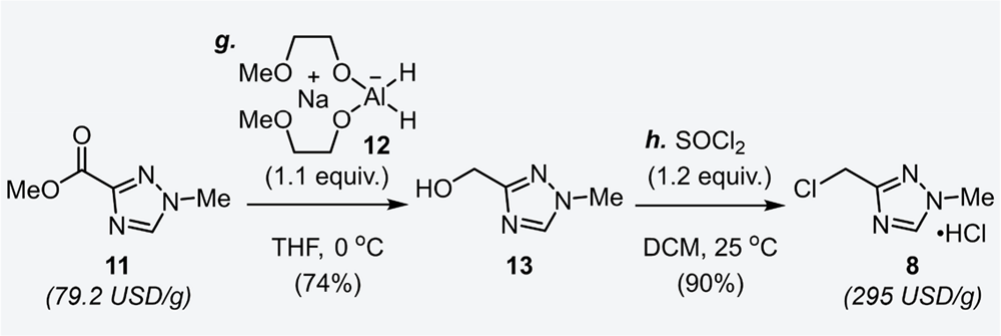
Manufacturing Route to 1,2,4-Triazole Motif **8** via Ester Reduction and Deoxychlorination

To establish a refined manufacturing process, synthesis of 1,2,4-triazole derivative **8** was initially investigated (Scheme 2). Starting from a commercially available compound **11**, reduction of the ester moiety was smoothly performed by sodium bis(2-methoxyethoxy)aluminum dihydride **12**, which can be purchased as a toluene solution and does not have hazardous pyrophoric nature like LiAlH_4_.^15,16^ Given the high solubility in water of the generated alcohol **13**, an extractive process had to be avoided. Therefore, potassium sodium tartrate, known as Rochelle salt, was added directly into the reaction mixture to form a chelate complex with aluminum.^17^ After removal of the aluminum residue by filtration, the crystals of **13** were directly isolated in 74% yield by switching to methyl *tert*-butyl ether (MTBE) as an antisolvent. Alcohol **13** was chlorinated immediately at room temperature using thionyl chloride (SOCl_2_) to afford the desired **8** in high yield.^18^

### Preparation of the Indazole Motif

Indazoles as pharmacophores have a broad variety of biological utilities, which have encouraged the development of novel synthetic methodologies for the synthesis of indazole derivatives.^19−21^ Several unique synthetic methods of indazoles have been reported, but most require harsh conditions and/or inaccessible starting compounds making it difficult to design scalable processes.^22−26^ Therefore, a mild preparation of indazole **10** needed to be optimized in anticipation of scale-up.

**Scheme 3:**
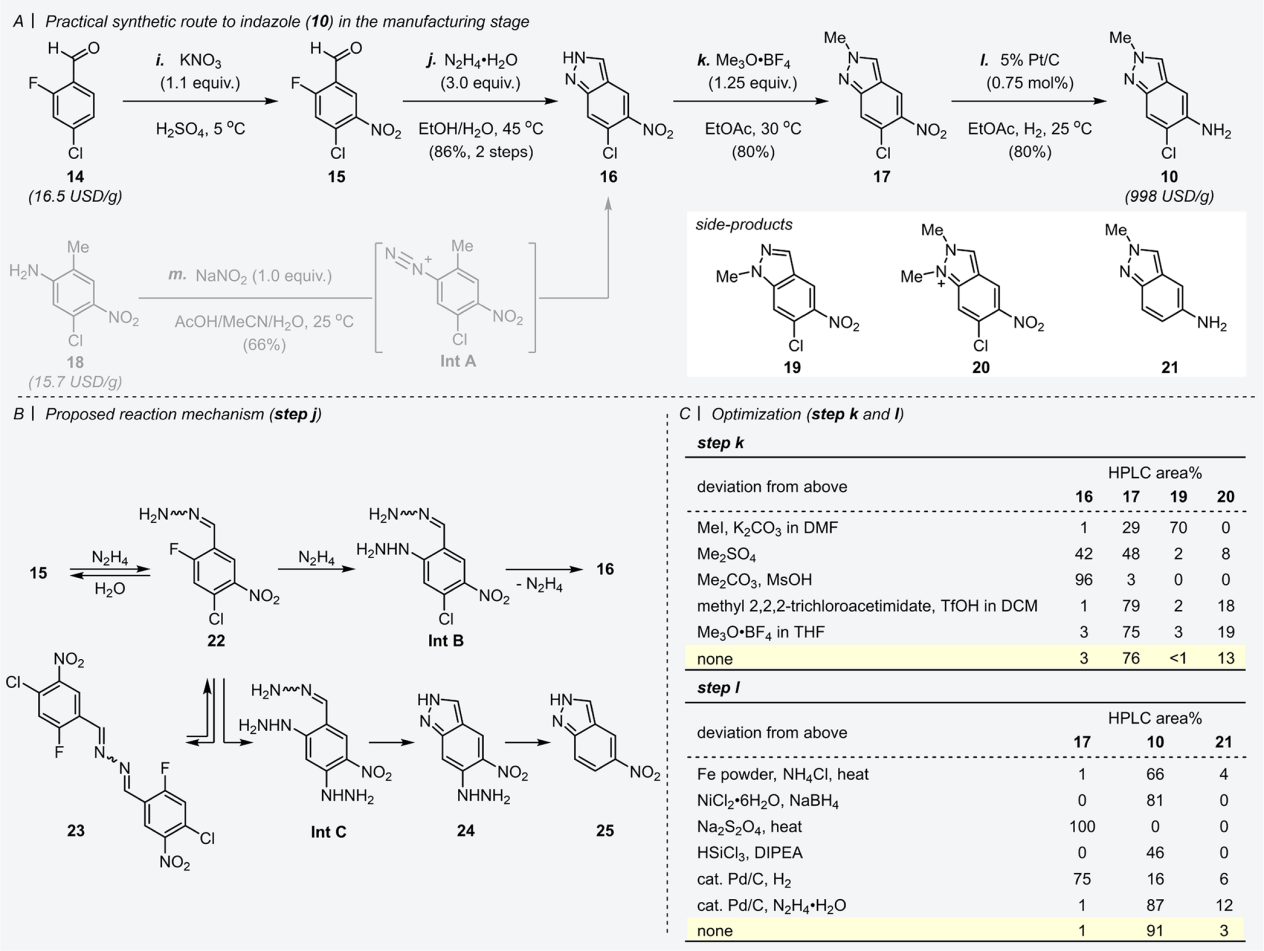
(A) Practical Synthetic Route to Indazole Motif **10** in the Manufacturing Stage. (B) Proposed Reaction Mechanism in Step j. (C) Optimizations of Steps k and l

Our synthesis began with electrophilic aromatic nitration of inexpensive feedstock aromatic aldehyde **14** under classical but reliable conditions (Scheme 3A, step i).^27^ Crystallization was directly conducted in the reaction mixture to afford the corresponding compound **15**, which could be used in the next reaction without drying. With reference to the pioneering study,^25^ cyclization of **15** was performed in the presence of hydrazine to form indazole motif **16** via formation of a hydrazone intermediate and a subsequent S_N_Ar reaction (step j). Through optimization of the reported protocol, we identified an excess amount of hydrazine as the key to achieving mild conditions, and **16** was directly obtained from EtOH/water cosolvent in 86% yield (from **14**, over 2 steps). While indazole **16** could be prepared from tetra-substituted benzene **18** via the diazonium compound (**Int A**) in one step (step m),^26^ the former synthetic route from **14** was superior in terms of yield and feasibility of scale-up.

The proposed reaction mechanism in step j is shown in Scheme 3B. The aromatic aldehyde **15** reacted with hydrazine to afford the corresponding hydrazone **22**. A reactive intermediate (**Int B**) was generated by substitution of the aryl fluoride **22** with another molecule of hydrazine, followed by the immediate cyclization of **Int B**. In the present step, excess hydrazine is expected to accelerate this S_N_Ar reaction. Indeed, when using 1.1 equiv. of hydrazine, high temperature (ca. 150 °C) was needed to proceed. Although impurity **23** was generated by the reaction with **15** and **22**, water as cosolvent enabled the equilibration between **22** and **23** to regenerate **22**. In addition, impurities **24** and **25** were formed via an intermediate (**Int C**). To support our proposed mechanism, all impurities **22**, **23**, **24**, and **25** were identified to control the quality of the final product.

With a scalable assembly method of indazole **16** in hand, the subsequent *N*-methylation was examined (Scheme 3C, top). Using trimethyloxonium tetrafluoroborate (Me_3_O·BF_4_, known as Meerwein’s reagent) gave the best result compared with other methylation reagents, such as methyl iodide, dimethyl sulfate, dimethyl carbonate, and methyl 2,2,2-trichloroacetimidate.^28,29^ The present reaction conditions using Me_3_O·BF_4_ could suppress the generation of side-products **19** and **20** to an acceptable level. It has been reported that the regioselectivity between two nitrogen atoms on the indazole depends on the nature of the alkylating reagents.^30^ Consistent with these reports, a trend of low regioselectivity was observed under basic conditions, and the acidic reagents improved the regioselectivity. Because methylation of **16** in tetrahydrofuran (THF) caused a problem in stirring due to a sticky slurry, we chose EtOAc as an optimal reaction solvent to afford **17** in sufficient isolated yield (80%). Next, the practical reduction conditions of the nitro group were explored (Scheme 3C, bottom).^31^ Although Béchamp reduction using iron under acidic condition is a powerful tool for converting aromatic nitro compounds into the corresponding anilines,^32^ iron-derived insoluble materials adhered to the reactor and were difficult to remove. Reduction by NaBH_4_ and catalytic NiCl_2_ was unsafe due to the difficulty of controlling hydrogen gas and the exothermic reaction.^33^ Various other conditions using Na_2_S_2_O_4_, HSiCl_3_, or palladium on carbon (Pd/C) under hydrogen atmosphere were investigated but resulted in insufficient conversion or failure to suppress the undesired dechlorination (generation of **21**). Fortunately, the desired reduction proceeded in the presence of Pd/C catalyst and N_2_H_4_ as a hydrogen source to give the aniline derivative **10** in 71% isolated yield. After screening of the heterogeneous catalyst and hydrogen source, 0.75 mol % of platinum on carbon (Pt/C) was found to perform effectively under hydrogen atmosphere to reduce the nitro group selectively as a scalable process (80% isolated yield).^34^ The scalability of this manufacturing process of **10** from **14** was demonstrated on the scale of several hundred kilograms.

### Manufacturing Process of Ensitrelvir

**Scheme 4:**
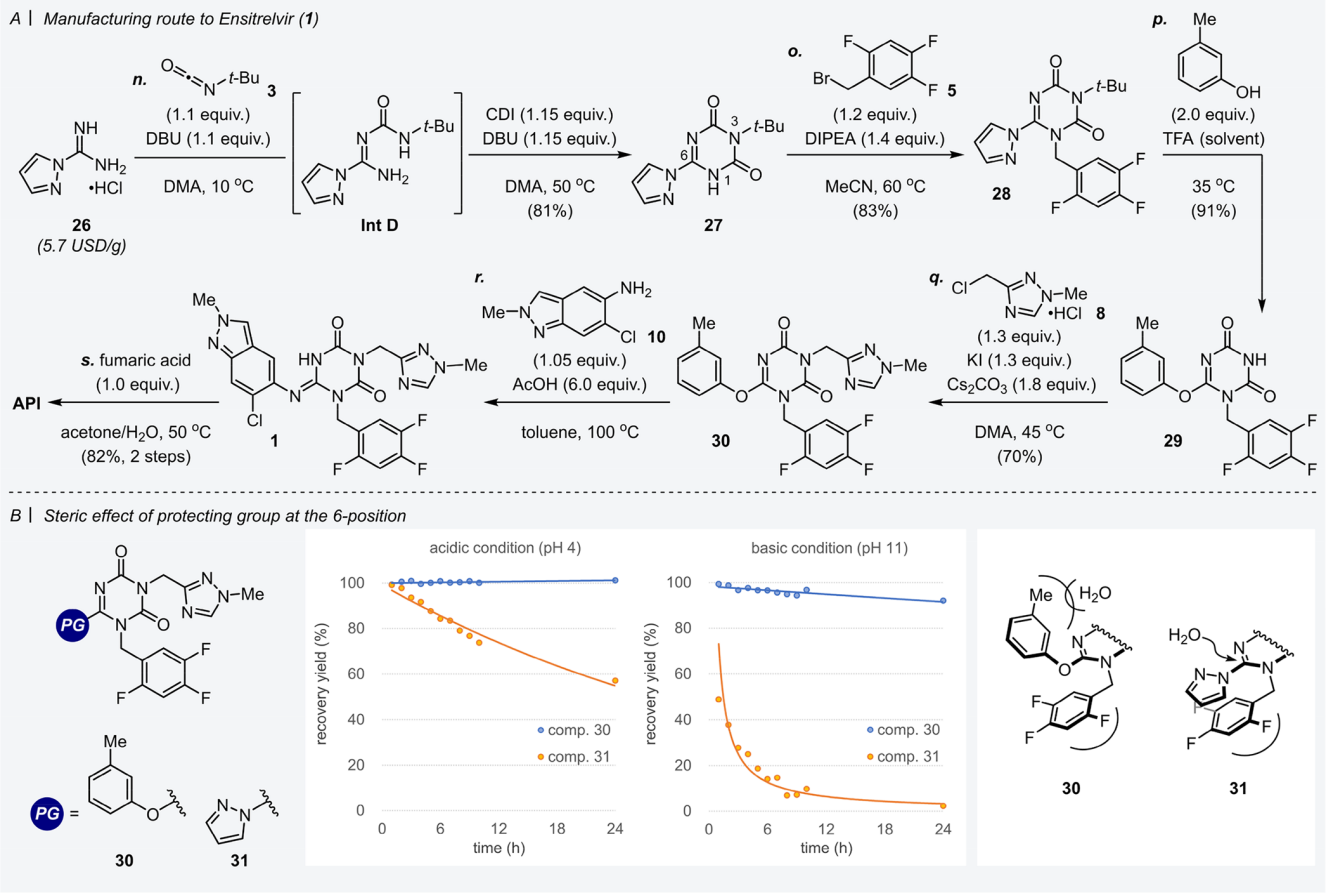
(A) Manufacturing Route to Ensitrelvir **1**. (B) Steric Effect of Protecting Group at the 6-Position Conditions of the stability test: To a mixture of **30** (or **31**) (10 mg), DMA (100 μL) and water (50 μL) was added acetic acid (2 μL, 1.5 equiv., acidic condition) or DIPEA (5 μL, 1.5 equiv., basic condition). After stirring for an adequate time at 25°C, the recovery yield of **30** (or **31**) measured by HPLC was plotted.

Having established an efficient synthesis of 1,2,4-triazole derivative **8** and indazole derivative **10**, we next turned to the synthesis of a 1,3,5-triazinone scaffold for the final convergent coupling. As described in Scheme 4A, our synthetic route of the 1,3,5-triazinone motif began with a multicomponent reaction of 1-amidinopyrazole **26**, *tert*-butyl isocyanate **3**, and 1,1′-carbonyldiimidazole (CDI) in *N*,*N*-dimethylacetamide (DMA, step n).^14^ To avoid the generation of the noxious sulfurous odor, the starting material *S*-ethylisothiourea **2** (Scheme 1) used at the early research and development stage was replaced by **26**. Compared with **2** (16.8 USD/g, more than 60 suppliers), **26** was inexpensive and easily available (5.7 USD/g, more than 90 suppliers).^35^ After the cyclization reaction of **26** with **3** and CDI, addition of 10% aqueous H_2_SO_4_ to the reaction mixture delivered the target 1,3,5-triazinone derivative **27** in 81% yield. For this neutralization, H_2_SO_4_ was better than HCl as a neutralizer because 1,8-diazabicyclo[5.4.0]undec-7-ene (DBU)-HCl salt was insoluble in DMA/water solution and there was the possibility of contaminating the crystals of **27**. Notably, it was found that the yield of **27** was improved by the fractional addition of DBU, i.e., two separate additions before and after the formation of the intermediate (**Int D**). This simple manipulation seems to minimize the decomposition of **3** caused by excess DBU to afford **Int D** with high conversion. *N*-Benzylation of **27** with 2,4,5-trifluorobenzyl bromide **5** proceeded smoothly using *N*,*N*-diisopropylethylamine (DIPEA) as a scalable organic base (step o). Direct isolation was also effective in step o, and the crystals of the desired molecule **28** could be obtained with high purity by simply adding water to the reaction medium.

Next, we investigated the removal of the *tert*-butyl moiety at the 3-position of compound **28** obviating the evaporation of corrosive acid, i.e., TFA. Unfortunately, only TFA (as solvent) could remove the *tert*-butyl group, with a failure of the desired deprotection using alternative acids (methanesulfonic acid, H_2_SO_4_, HCl, and BF_3_·Et_2_O). With the above results, a scalable neutralization method was explored to remove TFA; however, the deprotected compound bearing a pyrazolyl moiety was unstable under neutralization conditions. Thus, an alternative substituent to the pyrazolyl moiety was required to enhance the stability of the intermediate toward hydrolysis. After various substituents (e.g., phenol, imidazole, pyrrole, indazole, and indole derivatives) were investigated, *meta*-cresol was strategically substituted at the pyrazolyl position from the viewpoints of cost, isolation of intermediates and toxicity as well as stability. In other words, the deprotection at the 3-position and the protection of the 6-position were conducted simultaneously in the presence of *meta*-cresol in TFA to give the corresponding compound **29** (step p). Interestingly, an excess amount of *meta*-cresol also played a role in capturing the *tert*-butyl cation derived from the deprotection, and unexpected side-reactions could be prevented.^36,37^

Since the target compound **29** had better stability, the appropriate workup conditions for the removal of TFA were examined again. Aqueous sodium acetate (NaOAc) could neutralize TFA, and target compound **29** was crystallized directly from the reaction medium without an extraction procedure. In this case, the decomposition of **29** was suppressed, but **29** was obtained as cocrystals with *meta*-cresol, which affected the next alkylation step. The neutralization using triethylamine (Et_3_N) and subsequent extraction were unacceptable from the standpoint of loss of **29** during the extraction process. Neutralization by aqueous tripotassium phosphate (K_3_PO_4_) or trisodium citrate showed good performance without marked loss or decomposition of **29**. Finally, a practical workup method was established by neutralization and extraction using aqueous sodium hydroxide (NaOH), and subsequent crystallization from EtOAc/*n*-heptane. The above process, which avoids the evaporation of TFA, offered us a wide selection of production site and equipment as no special corrosion protection equipment is required.

The strategy of introducing a *meta*-cresolyl moiety to improve hydrolysis resistance also had a pivotal impact on the next *N*-alkylation (step q). A preliminary stability test between the compounds bearing different substituents **30** and **31** revealed the significance of protecting groups at the 6-position (Scheme 4B). Namely, 1,3,5-triazinone bearing a *meta*-cresolyl moiety **30** was more stable than **31** under both weak acidic (pH 4) and basic (pH 11) conditions that mimicked step q (Scheme 4B, center). Differences in stability might be manifested by electronic effects and/or steric factors. In particular, steric hindrance of the *meta*-cresolyl moiety compared to the flat pyrazolyl moiety probably enhances the stability toward hydrolysis (Scheme 4B, right). In addition to the above results, using cesium carbonate (Cs_2_CO_3_) instead of K_2_CO_3_ improved the reproducibility of the reaction. Moreover, **30** could be crystallized directly from the reaction mixture to obtain a 70% yield.

Installation of indazole **10** was the most important step affecting the quality of Ensitrelvir **1**. Therefore, both high efficiency and robustness were required in the final step. The *meta*-cresolyl moiety also had the crucial role of a good leaving group in this step, and the targeted **1** was generated smoothly after heating in toluene with **10** in the presence of acetic acid (step r). When using other substrates bearing different leaving groups, such as ethylsulfanyl **9** or pyrazolyl **31** moieties instead of the *meta*-cresolyl moiety, the yield of **1** was insufficient and/or hard-to-use reagents were required for scalable processes (see the Supporting Information). The present results indicated that the synthetic strategy using *meta*-cresol, which was irreplaceable not only as a protecting group but also as a leaving group, was essential. **1** was directly isolated from the reaction mixture after cooling and then used in the next step without drying. Crystallization of **1** with fumaric acid in cosolvent (acetone and water) gave cocrystals of **1** with sufficient purity for pharmaceutical medicine (step s). The process to **1** just described involving the preparation of **8** and **10** showed good to excellent yield at each step, and was successfully applied to synthesis on the scale of several hundred kilograms. All the target compounds were isolated by crystallization, completely avoiding purification by column chromatography.

## Conclusion

We have developed a practical route to Ensitrelvir **1**. The unprecedented strategy utilizing a *meta*-cresolyl moiety enhanced the stability of intermediate compounds and enabled scalable manufacturing of **1**. The 6-step longest linear sequence (from **26**) delivers the target molecule in 35.1% yield. This overall yield was improved by approximately 7-fold compared to the earlier process used in the early research and development stage (4.8%). Our convergent approach to the synthesis of **1** made the manufacturing process concise, streamlined, and short. Direct crystallization was achieved in 9 out of the 12 steps, thus establishing a greener process without silica-gel column chromatographic purification.^38^ In addition, the present practical process settled all the issues that made large-scale synthesis difficult, i.e., removal of corrosive acid by evaporation and the generation of noxious odors from thiol derivatives. We believe the development of this practical process for manufacturing of **1** contributes to re-establishing the safety and security of society and addressing the COVID-19 pandemic.^39^

## Supporting Information Available

Experimental procedures, additional optimizations, and characterization data (PDF)
Transparent Peer Review report available (PDF)
