# Supplementary material for: Development of a Manufacturing Process toward the Convergent Synthesis of the COVID-19 Antiviral Ensitrelvir
Source: ACS Cent Sci. 2023 Apr 7;9(4):836–43. doi: 10.1021/acscentsci.2c01203 (PMC10108738; doi:10.1021/acscentsci.2c01203)
Supplement: Supplementary file 1 — oc2c01203_si_001.pdf [file oc2c01203_si_001.pdf]

Supporting Information

Development of Manufacturing Process toward the Convergent Synthesis of the COVID-19 Antiviral Ensitrelvir

Takahiro Kawajiri[*†], Akihito Kijima[†], Atsuhiro Iimuro[‡], Eisaku Ohashi[†], Katsuya Yamakawa[†], Kazushi Agura[†], Kengo Masuda[‡], Kensuke Kouki[‡], Koji Kasamatsu[‡], Shuichi Yanagisawa[†], Sho Nakashima[†], Setsuya Shibahara[†], Takashi Toyota[†], Takafumi Higuchi[†], Takahiro Suto[‡], Tadashi Oohara[†], Toshikatsu Maki[†], Naoto Sahara[†], Nobuaki Fukui[†], Hisayuki Wakamori[†], Hidaka Ikemoto[†], Hiroaki Murakami[†], Hiroyasu Ando[†], Masahiro Hosoya[†], Mizuki Sato[†], Yusuke Suzuki[†], Yuta Nakagawa[†], Yuto Unoh[‡], Yoichi Hirano[†], Yoshitomo Nagasawa[†], Satoshi Goda[†], Takafumi Ohara[†], and Takayuki Tsuritani[†]

[†]API R&D Laboratory, Research Division, Shionogi & Co., Ltd.,
1-3, Kuise Terajima 2-Chome, Amagasaki, Hyogo 660-0813, Japan

[‡]Laboratory for Medicinal Chemistry Research, Research Division, Shionogi & Co., Ltd.,
1-1, Futaba-cho 3-Chome, Toyonaka, Osaka 561-0825, Japan

*Email: takahiro.kawajiri@shionogi.co.jp

Table of Contents

General Information S2

HPLC method S3

Manufacturing route of Ensitrelvir S4

Additional optimization S5

Experimental Procedures and Characterization

- Compound **13** S8
- Compound **8** S9
- Compound **15** S10
- Compound **16** S11
- Compound **17** S12
- Compound **10** S13
- Compound **27** S14
- Compound **28** S15
- Compound **29** S16
- Compound **30** S17
- Compound **1** S18
- Compound **API** S19

Characterization of other compounds S20

^{1}H and ^{13}C NMR spectra of synthesized compounds. S22

References S54

General Information

All reaction were carried out under an inert nitrogen atmosphere. Unless otherwise noted, the substrates and anhydrous solvents were purchased from commercial sources and used without further purification. NMR spectra were recorded on a Bruker AVANCE III HD 400 spectrometer at room temperature using residual solvent as an internal standard ($CDCl_3$ at 7.27 ppm, ^1H NMR, 77.00 ppm ^{13}C NMR; DMSO-d_6 at 2.50 ppm, ^1H NMR, 39.51 ppm ^{13}C NMR; pyridine-d_5 at 8.74 ppm, ^1H NMR; THF-d_8 at 67.57 ppm, ^{13}C NMR). The following abbreviations (or combinations thereof) were used to denote multiplicities: s = singlet, d = doublet, m = multiplet (complex pattern), brs = broad signal. Mass measurements for high-resolution mass spectra (HRMS) were performed on a Waters BioAccord calibrated against sodium iodide and rubidium iodide clusters and using a LeuEnk lockmass.

In all steps, unless otherwise noted, the solvent was concentrated under reduced pressure and the crystallization occurred without seeding process.

Abbreviations

AcOH = acetic acid, AmOH = amyl alcohol, $BF_3 \cdot Et_2O$ = boron trifluoride diethyl ether complex, BuOH = butyl alcohol, CDI = carbonyldiimidazole, $CHCl_3$ = chloroform, Cs_2CO_3 = cesium carbonate, DBU = 1,8-diazabicyclo[5.4.0]undec-7-ene, DCM = dichloromethane, DIPEA = *N*,*N*-diisopropylethylamine, DMA = *N*,*N*-dimethyl acetamide, DMBA = 2,2-dimethylbutylic acid, DMF = *N*,*N*-dimethyl formamide, DMSO = dimethyl sulfoxide, Et_3N = triethylamine, EtOH = ethanol, EtOAc = ethyl acetate, HCl = hydrogen chloride, $HSiCl_3$ = trichlorosilane, H_2SO_4 = sulfuric acid, K_2CO_3 = potassium carbonate, $KHCO_3$ = potassium bicarbonate, KI = potassium iodide, KNO_3 = potassium nitrate, K_3PO_4 = tripotassium phosphate, $LiAlH_4$ = lithium aluminium hydride, LiHMDS = lithium hexamethyldisilazide, MeCN = acetonitrile, Me_2CO_3 = dimethyl carbonate, MeI = methyl iodide, $Me_3O \cdot BF_4$ = trimethyloxonium tetrafluoroborate, MeOH = methanol, Me_2SO_4 = dimethyl sulfate, MsOH = methanesulfonic acid, MTBE = methyl *tert*-butyl ether, $NaBH_4$, = sodium borohydride, NaCl = sodium chloride, $NaHCO_3$ = sodium bicarbonate, $NaNO_3$ = sodium nitrate, $Na_2S_2O_4$ = sodium hydrosulfite, NaOAc = sodium acetate, NaOH = sodium hydroxide, NaO*t*-Bu = sodium *tert*-butoxide, NH_4Cl = ammonium chloride, $N_2H_4 \cdot H_2O$ = hydrazine monohydrate, $NiCl_2 \cdot 6H_2O$ = nickel(II) chloride hexahydrate, Pd/C = palladium on carbon, Pt/C = platinum on carbon, Rh/C = rhodium on carbon, $SOCl_2$ = thionyl chloride, TFA = trifluoro acetic acid, TfOH = trifluoromethanesulfonic acid, THF = tetrahydrofuran, $TiCl_4$ = titanium(IV) chloride, $Yb(OTf)_3$ = ytterbium(III) trifluoromethanesulfonate.

HPLC method

Method A (Scheme 3C)

Column : Xselect CSH C18, 150 × 4.6 mm, 3.5 µm (Waters)
Mobile phase A : 0.1% formic acid aq.
Mobile phase B : MeCN
Flow rate : 1.0 mL/min
Injection volume : 5 µL
Column temperature : 40°C
Wavelength : 254 nm
Gradient program

Time (min)	Mobile phase A (%)	Mobile phase B (%)
0.00	95	5
17.00	5	95
20.00	5	95
20.01	95	5
30.00	95	5

Method B (Scheme 4B)

Column : Xselect CSH Fluoro-Phenyl, 150 × 4.6 mm, 3.5 µm (Waters)
Mobile phase A : 0.1% phosphoric acid aq.
Mobile phase B : MeCN
Flow rate : 1.0 mL/min
Injection volume : 5 µL
Column temperature : 40°C
Wavelength : 255 nm
Gradient program

Time (min)	Mobile phase A (%)	Mobile phase B (%)
0.00	80	20
6.00	80	20
27.00	58	42
31.00	50	50
34.00	5	95
34.01	80	20
45.00	80	20

Manufacturing route of Ensitrelvir

S1 | *Practical route of 1,2,4-triazole (**8**)*

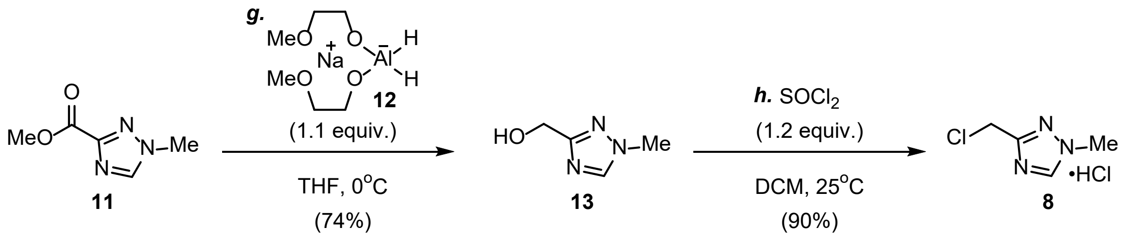

total yield and step count	
38.2% (from **11**, 5 steps)	
45.1% (from **14**, 6 steps)	
35.1% (from **26**, 6 steps, LLS)	
**operation**	
extraction	: 3/12 steps
chromatography	: 0/12 steps
drying	: 9/12 steps

S2 | *Practical route of indazole (**10**)*

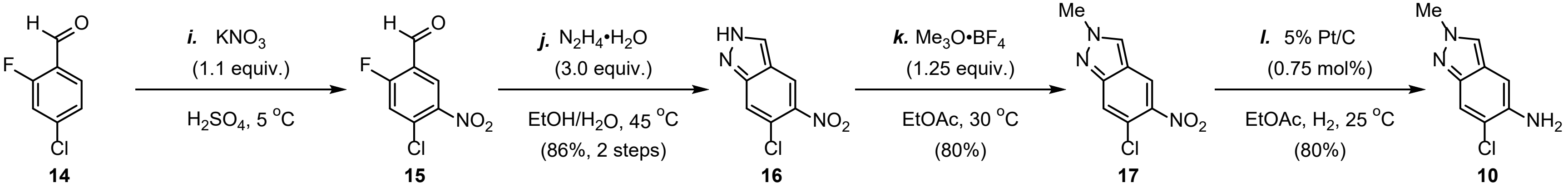

S3 | *Practical route of Ensitrelvir (**1**)*

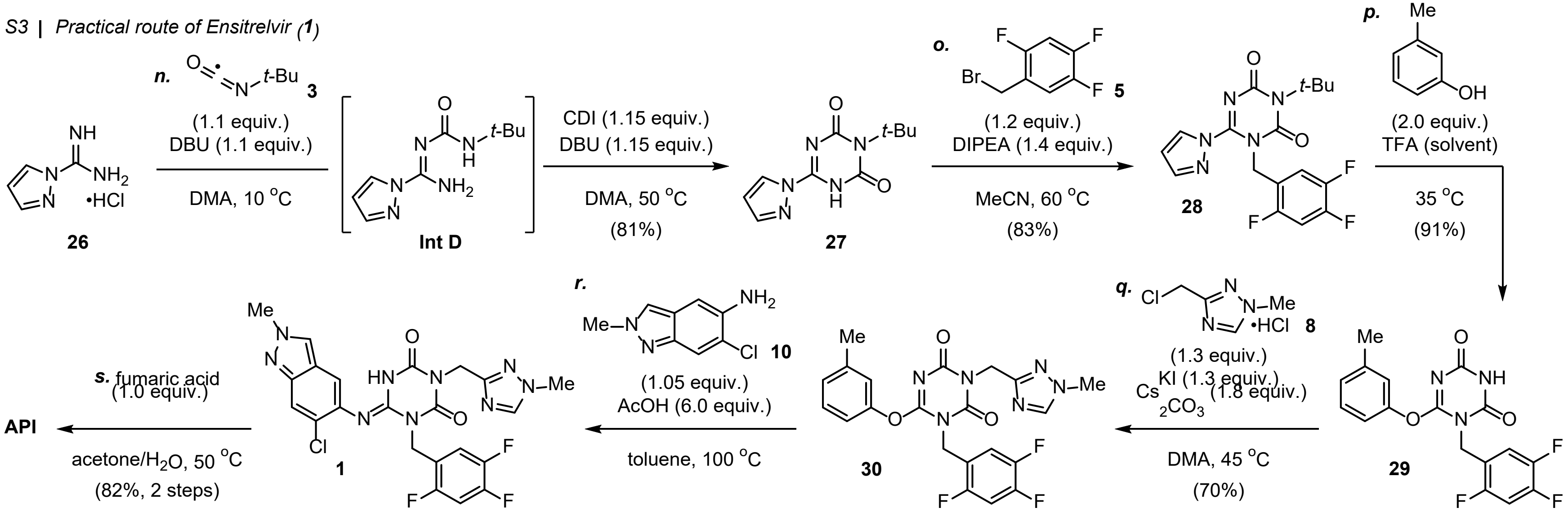

Additional optimization

Optimization of step k.

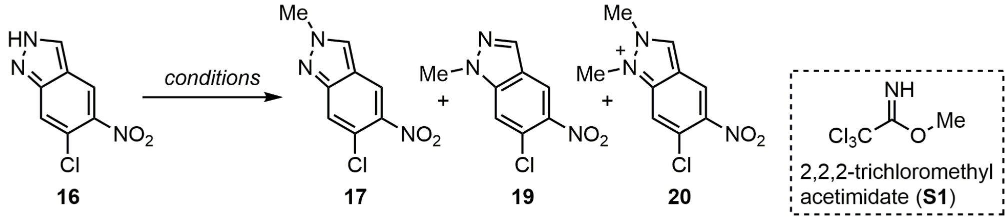

entry	reagent	additive	solvent	temp. (°C)	time (h)	HPLC pa%[a]			
						**16**	**17**	**19**	**20**
1	MeI	none	DMF	80	3	84	14	1	1
2	MeI	Et_3N	DMF	50	2	85	13	2	1
3	MeI	K_2CO_3	DMF	25	1	1	29	70	<1
4	MeI	NaO*t*-Bu	*tert*-BuOH	40	7.5	12	42	46	<1
5	Me_2SO_4	none	MeCN	70	7	23	57	2	18
6	Me_2SO_4	none	EtOAc	70	7	42	48	2	8
7	Me_2SO_4	none	acetone	50	3	92	6	<1	<1
8	Me_2SO_4	none	MeCN/DMA	70	7	66	30	1	3
9	Me_2SO_4	none	THF/DMA	70	7	69	28	1	2
10	Me_2SO_4	none	toluene/DMA	70	7	26	61	3	10
11	Me_2SO_4	Et_3N	toluene/DMA	50	4	99	<1	<1	<1
12	Me_2SO_4	none	acetone/DMA	50	1	88	10	<1	1
13	Me_2SO_4	$NaHCO_3$	acetone/DMA	50	1	77	14	9	0
14	Me_2CO_3	none	MeOH	80	2	>99	<1	<1	0
15	Me_2CO_3	Na_2CO_3	MeOH	80	2	>99	<1	<1	0
16	Me_2CO_3	DBU	MeOH	80	2	97	<1	<1	<1
17	Me_2CO_3	$Yb(OTf)_3$	MeOH	80	2	>99	<1	<1	0
18	Me_2CO_3	$TiCl_4$	MeOH	80	2	73	1	<1	25
19	Me_2CO_3	$TiCl_4$	EtOAc	80	2	70	<1	14	16
20	Me_2CO_3	MsOH	Me_2CO_3	90	7	96	3	0	0
21	**S1**	TfOH	DCM	25	3	1	79	2	18
22	**S1**	TfOH	DCM/MeOH	25	8	66	32	<1	1
23	$Me_3O \cdot BF_4$	none	DCM	25	8	9	76	0	15
24	$Me_3O \cdot BF_4$	none	toluene	25	1	82	15	<1	3
25	$Me_3O \cdot BF_4$	none	THF	25	1	3	75	3	19
26	$Me_3O \cdot BF_4$	none	2-MeTHF	25	1.5	57	37	2	5
27	$Me_3O \cdot BF_4$	none	MeCN	25	2.5	10	71	1	18
28	$Me_3O \cdot BF_4$	none	EtOAc	0	6	17	55	2	26
29	$Me_3O \cdot BF_4$	none	EtOAc	35	8	3	76	<1	13

a) HPLC method A.

Optimization of step l

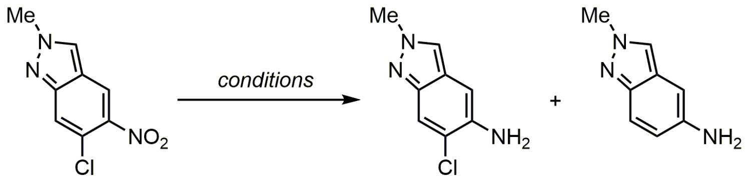

entry	reagent	additive	solvent	temp. (°C)	time (h)	HPLC area%[a)]		
						**17**	**10**	**21**
1	Fe powder	NH_4Cl	$THF/EtOH/H_2O$	70	1	1	66	4
2	$NiCl_2 \cdot 6H_2O$	$NaBH_4$	$MeCN/H_2O$	25	0.5	0	81	0
3	$Na_2S_2O_4$	none	DMSO/THF	90	2	100	0	0
4	$HSiCl_3$	DIPEA	MeCN	25	18	0	46	0
5	2.1% Pd/fibroin	H_2	MeOH	25	21	1	35	44
6	5% Pd/C (en)	$N_2H_4 \cdot H_2O$	EtOAc	25	46.5	0	96	4
7	5% Rh/C	$N_2H_4 \cdot H_2O$	EtOAc	25	3	0	81	19
8	10% Pd/C	H_2	EtOAc	25	15.5	75	16	6
9	10% Pd/C	$N_2H_4 \cdot H_2O$	EtOAc	25	18.5	1	87	12
10	5% Pt/C	H_2	THF	25	5	0	83	9
11	5% Pt/C	H_2	EtOAc	25	5	1	91	3

a) HPLC method A.

Optimization of step r

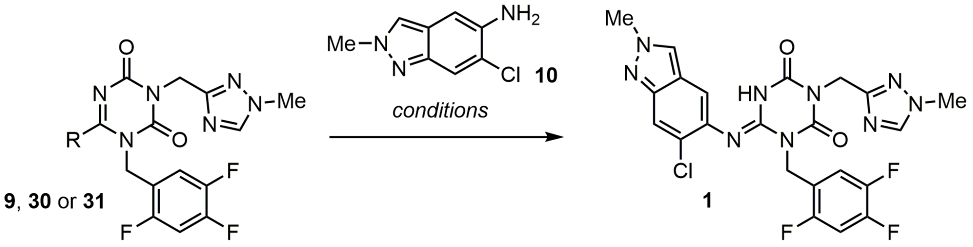

entry	reagent	R	solvent	temp. (°C)	yield of **1** (%)[a)]
1	LiHMDS	EtS	THF	0	25
2	$AgNO_3$	EtS	DMBA	90	23
3	none	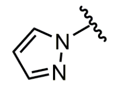	*tert*-AmOH	120	56
4	none	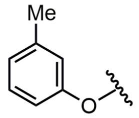	DMBA	100	55
5	AcOH	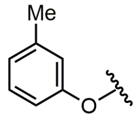	*tert*-BuOH	100	39
6	AcOH	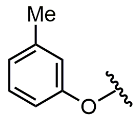	toluene	100	82

a) Isolated yield.

Experimental Procedures and Characterization

Compound **13**, (1-methyl-1*H*-1,2,4-triazol-3-yl)methanol

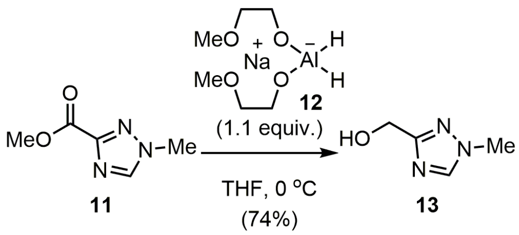

To a mixture of methyl 1-methyl-1*H*-1,2,4-triazole-3-carboxylate (**11**; 500.0 kg, 3545 mol) in THF (5108 L, 10.0 vol) was added bis(2-methoxyethoxy)aluminum hydride (**12**; 70% in toluene solution, 1120 kg, 3878 mol) at 0°C. After stirring for 3 h, acetone (354 L, 0.7 vol) was added to the reaction mixture. Then potassium sodium tartrate tetrahydrate (803 kg, 2845 mol) and THF (3505 L, 7.0 vol) were added to the reaction mixture at 25°C. After stirring for 5 h, the mixture was filtered and the filtered solid was washed with THF (2347 kg). The obtained filtrate solution was concentrated to 3.0 wt. To the concentrate was added MTBE (756 L, 1.5 vol) at 25°C, then seed crystal was added. After stirring for the adequate time, MTBE (4044 L, 8.0 vol) was added to the mixture. The mixture was concentrated to 5.5 wt. Then, MTBE (3503 L, 7.0 vol) was added again to the concentrate. The mixture was cooled to − 5°C and filtered. The filtered solid was washed with MTBE (1025 L, 2.1 vol). The obtained solid was dried under reduced pressure at 35°C to afford (1-methyl-1*H*-1,2,4-triazol-3-yl)methanol (**13**; 308.5 kg, 73.7%).

Physical state: white solid.

Melting point: 81°C

^{1}H NMR (400 MHz, DMSO-d_6): δ 8.34 (s, 1H), 5.17 (t, J = 5.5 Hz, 1H), 4.40 (d, J = 5.3 Hz, 2H), 3.82 (s, 3H).

^{13}C{^{1}H} NMR (100 MHz, DMSO-d_6): δ 163.5, 144.8, 56.7, 35.5.

HRMS-ESI (*m/z*): $[M + H]^+$ calcd for $[C_4H_8N_3O]^+$ 114.0667, found 114.0670.

Compound **8** (3-(chloromethyl)-1-methyl-1H-1,2,4-triazole hydrochloride)

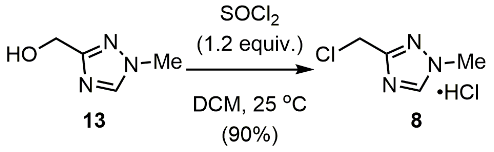

To a mixture of (1-methyl-1*H*-1,2,4-triazol-3-yl)methanol (**13**; 308.0 kg, 2723 mol) in DCM (4634 L, 15.0 vol) was added $SOCl_2$ (359.6 kg, 3023 mol) at 25°C. After stirring for 20 h, MTBE (1537 L, 5.0 vol) was added to the reaction mixture. The mixture was stirred at 25°C for 35 h and filtered. The filtered solid was washed with MTBE (1063 L, 3.5 vol). The obtained solid was dried under reduced pressure at 25°C to afford 3-(chloromethyl)-1-methyl-1*H*-1,2,4-triazole·hydrochloride (**8**; 419.64 kg, 89.9%).

Physical state: white solid.

Melting point: 69–70°C

^{1}H NMR (400 MHz, DMSO-*d*$_6$): δ 11.63 (brs, 1H), 8.77 (s, 1H), 4.76 (s, 2H), 3.90 (s, 3H)

^{13}C{^{1}H} NMR (100 MHz, DMSO-*d*$_6$): δ 158.5, 145.4, 37.3, 36.4.

HRMS-ESI (*m*/*z*): $[M + H]^+$ calcd for $[C_4H_7ClN_3]^+$ 132.0328, found 132.0324.

Compound **15**, 4-chloro-2-fluoro-5-nitrobenzaldehyde

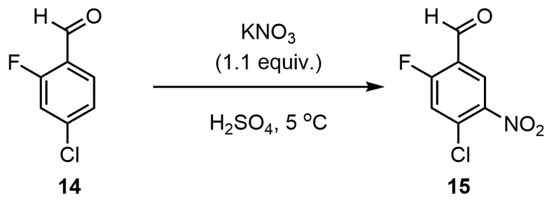

To a mixture of 4-chloro-2-fluoro-benzaldehyde (**14**; 235.0 kg, 1482 mol) in 98% H_2SO_4 aq. (2162.0 kg, 5.0 vol) was added KNO_3 (164.8 kg, 1630 mol) at 5°C. After stirring for 5 h, the reaction mixture was added to water (2350 L, 10.0 vol) at 5°C. Then the resulting slurry was filtered. The filtered solid was washed with water (2350 L, 10.0 vol × 3). The obtained wet solid of 4-chloro-2-fluoro-5-nitrobenzaldehyde (**15**; 292.4 kg) was used in next step without drying.

Physical state: white solid.

Melting point: 59°C

^{1}H NMR (400 MHz, $CDCl_3$): δ 10.31 (s, 1H), 8.46 (d, J = 6.6 Hz, 1H), 7.47 (d, J = 9.2 Hz, 1H)

^{13}C{^{1}H} NMR (100 MHz, $CDCl_3$): δ 183.6 (d, $J_{C\text{-}F}$ = 5.1 Hz), 164.4 (d, $J_{C\text{-}F}$ = 67.0 Hz), 144.9 (d, $J_{C\text{-}F}$ = 4.0 Hz), 135.0 (d, $J_{C\text{-}F}$ = 11.7 Hz), 126.4 (d, $J_{C\text{-}F}$ = 4.4 Hz), 123.1 (d, $J_{C\text{-}F}$ = 11.0 Hz), 120.7 (d, $J_{C\text{-}F}$ = 24.9 Hz)

HRMS-ESI (*m*/*z*): $[M + H]^+$ calcd for $[C_7H_4ClFNO_3]^+$ 203.9862, found 203.9864.

Compound **16**, 6-chloro-5-nitro-2*H*-indazole

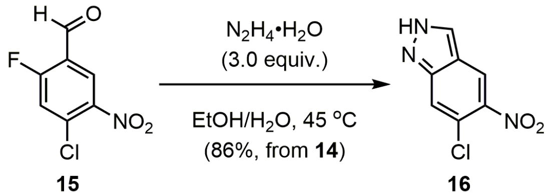

The molar equivalent, vol and weights in this step were based on the amount of compound **14** used.

To a mixture of hydrazine monohydride (204.8 kg, 4091 mol) in EtOH (2191 kg, 11.7 vol) and water (1943 L, 8.3 vol) was added wet 4-chloro-2-fluoro-5-nitrobenzaldehyde (292.4 kg) at 45°C. After stirring for 11.5 h, 8% $KHCO_3$ aq. (1858 kg) was added to the reaction mixture at 45°C. Then the resulting slurry was cooled to 5°C and filtered. The filtered solid was washed with a mixture of water and EtOH (water/EtOH = 5/2 vol/vol; 5386 L). The obtained solid was dried under reduced pressure at 50°C to afford 6-chloro-5-nitro-2*H*-indazole (**16**; 252.1 kg, 86.1% for 2 steps).

Physical state: yellowish red solid.

Melting point: 240°C

^{1}H NMR (400 MHz, $CDCl_3$): δ 8.42 (s, 1H), 8.23 (d, J = 1.1 Hz, 1H), 7.68 (d, J = 0.7 Hz, 1H)

^{13}C{^{1}H} NMR (100 MHz, DMSO-*d*$_6$): δ 142.1, 140.7, 136.6, 123.0, 120.9, 120.7, 113.3.

HRMS-ESI (*m*/*z*): $[M + H]^+$ calcd for $[C_7H_5ClN_3O_2]^+$ 198.0070, found 198.0069.

Compound **17**, 6-chloro-2-methyl-5-nitro-2*H*-indazole

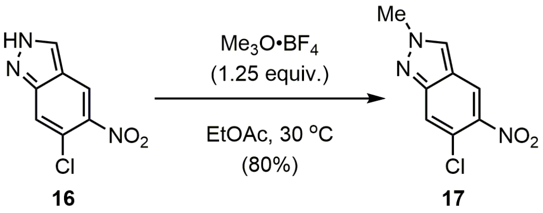

To a mixture of 6-chloro-5-nitro-2*H*-indazole (**16**; 160.0 kg, 810 mol) in EtOAc (2336 L, 14.6 vol) was added $Me_3O{\cdot}BF_4$ (149.7 kg, 1012 mol) at 30 °C. After stirring for 10 h, MeOH (379.4 kg, 3.0 vol) was added to the resulting mixture. The reaction mixture was mixed with 10% $NaHCO_3$ aq., and the organic layer was separated. The organic layer was washed with 6% NaCl aq., and then the organic layer was separated off. The organic layer was mixed with activated carbon (80.1 kg, 0.5 wt) and THF (1120 L, 7.0 vol). After stirring for an adequate time, activated carbon was separated off. The filtrate was concentrated under reduced pressure, and then the amount of solution was adjusted to 8.3 wt with THF. The solution was mixed with *n*-heptane (528.0 L, 3.3 vol) and stirred at 33°C, and then seed crystal was added. *n*-Heptane (2912 L, 18.2 vol) was added again to the slurry at 33°C. Then the resulting slurry was cooled to − 8°C and filtered. The filtered solid was washed with a mixture of *n*-heptane (436.8 L, 2.7 vol) and THF (107.2 L, 0.7 vol). The obtained solid was dried under reduced pressure at 50°C to afford 6-chloro-2-methyl-5-nitro-2*H*-indazole (**17**; 136.86 kg, 79.8%).

Physical state: light yellowish red solid.

Melting point: 152°C

^{1}H NMR (400 MHz, $CDCl_3$): δ 8.31 (s, 1H), 8.13 (s, 1H), 7.81 (s, 1H), 4.27 (s, 3H).

^{13}C{^{1}H} NMR (100 MHz, $CDCl_3$): δ 148.6, 143.4, 127.1, 123.2, 120.0, 119.9, 118.8, 41.0.

HRMS-ESI (*m*/*z*): $[M + H]^+$ calcd for $[C_8H_7ClN_3O_2]^+$ 212.0227, found 212.0226.

Compound **10**, 6-chloro-2-methyl-2*H*-indazol-5-amine

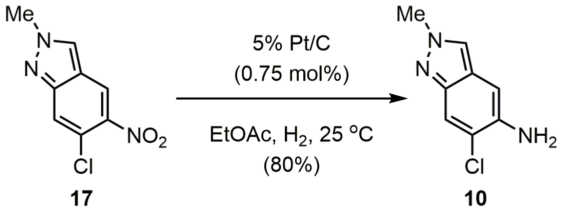

A mixture of 6-chloro-2-methyl-5-nitro-2*H*-indazole (**17**; 187.3 kg, 885 mol) and 5% Pt/C (59.0 kg, 6.6 mol) in EtOAc (1874.6 L, 10.0 vol) was stirred under hydrogen atmosphere at 25°C for 5.5 h. After the reaction, Pt/C was filtered off, and the cake was washed with EtOAc (838.0 L, 10.0 vol). The combined filtrates were mixed with 0.1% HCl aq., and the organic layer was separated. The organic layer was mixed with 0.14% NaOH aq., and then the organic layer was separated. The separated organic layer was washed with water, and then the organic layer was separated off. Above separated aqueous layers were extracted in order with EtOAc (3373.6 L, 18.0 vol). Then the separated organic layers were combined and concentrated under reduced pressure, and then the amount of solution was adjusted to 6.0 wt with EtOAc. The seed crystal was added to the mixture at 25°C. *n*-Heptane (2998.2 L, 16.0 vol) was added to the slurry at 25°C. Then the resulting slurry was cooled to 0°C and filtered. The filtered solid was washed with a mixture of *n*-heptane and EtOAc (*n*-heptane/EtOAc = 3/1 vol/vol; 1143 L). The obtained solid was dried under reduced pressure at 50°C to afford 6-chloro-2-methyl-2*H*-indazol-5-amine (**10**; 128.8 kg, 80.1%).

Physical state: light brown solid.

Melting point: 129°C

^{1}H NMR (400 MHz, $CDCl_3$): δ 7.69 (s, 1H), 7.61 (s, 1H), 6.87 (s, 1H), 4.13 (s, 3H), 3.95 (brs, 2H)

^{13}C{^{1}H} NMR (100 MHz, $CDCl_3$): δ 145.1, 137.5, 124.4, 121.7, 121.3, 117.4, 100.6, 40.2.

HRMS-ESI (*m*/*z*): $[M + H]^+$ calcd for $[C_8H_9ClN_3]^+$ 182.0485, found 182.0482.

Compound **27**, 3-(*tert*-butyl)-6-(1*H*-pyrazol-1-yl)-1,3,5-triazine-2,4(1*H*,3*H*)-dione

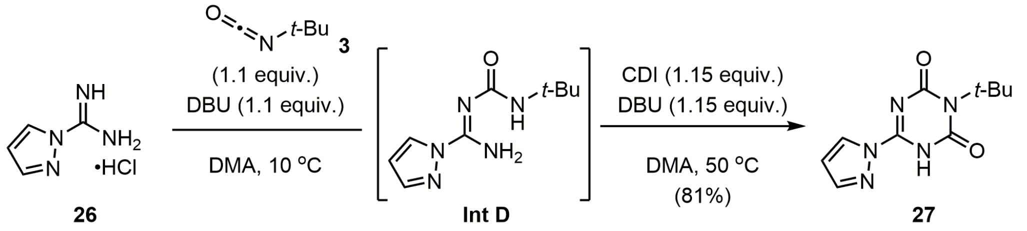

To a mixture of 1-amidinopyrazole·hydrochloride (**26**; 375.0 kg, 2558 mol) and DBU (428.4 kg, 2814 mol) in DMA (2610.0 kg, 7.4 vol) were added *tert*-butyl isocyanate (**3**; 279.0 kg, 2814 mol) at 10 °C. After stirring for 0.5 h, CDI (477.1 kg, 2942 mol) and DBU (447.9 kg, 2942 mol) were added to the reaction mixture. After stirring at 50°C for 4 h, MeOH (248 L, 6230 mol) was added to the reaction mixture at 20°C. The mixture was adjusted to pH 2.5 with 10% H_2SO_4 aq. at 20°C. Then the resulting slurry was cooled to 5°C and filtered. The filtered solid was washed with a mixture of water and MeOH (water/MeOH = 4/1 vol/vol; 800.0 L). The obtained wet solid of 3-(*tert*-butyl)-6-(1*H*-pyrazol-1-yl)-1,3,5-triazine-2,4(1*H*,3*H*)-dione (**27**; 530.0 kg) was used in next step without drying (assay yield: 80.7%).

Physical state: white solid.

Melting point: 162°C

^{1}H NMR (400 MHz, $CDCl_3$): δ 9.47 (brs, 1H), 8.42 (d, J = 2.9 Hz, 1H), 7.81 (d, J = 1.5 Hz, 1H), 6.57 (dd, J = 2.9, 1.6 Hz, 1H), 1.72 (s, 9H),

^{13}C{^{1}H} NMR (100 MHz, $CDCl_3$): δ 148.4, 145.2, 129.2, 111.2, 61.9, 29.6, 29.1.

HRMS-ESI (*m/z*): [M + Na]$^+$ calcd for $[C_{10}H_{13}N_5O_2Na]^+$ 258.0967, found 258.0968.

Compound **28**, 3-(*tert*-butyl)-6-(1*H*-pyrazol-1-yl)-1-(2,4,5-trifluorobenzyl)-1,3,5-triazine-2,4(1*H*,3*H*)-dione

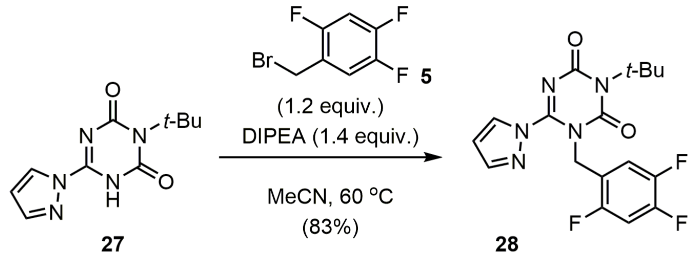

Wet 3-(*tert*-butyl)-6-(1*H*-pyrazol-1-yl)-1,3,5-triazine-2,4(1*H*,3*H*)-dione (**27**, 530.0 kg) was divided into two halves. One half was dissolved in MeCN. Then the mixture was concentrated under reduced pressure and adjusted to 8.5 vol with MeCN. DIPEA (174.1 kg, 1347 mol) and 2,4,5-trifluorobenzyl bromide (279.7 kg, 1243 mol) were added to the above mixture, and the mixture was warmed to 60°C. After stirring for 5 h, water (1105 L, 5.0 vol) was added to the reaction mixture at 25°C. Then the resulting slurry was cooled to 5°C and filtered. The filtered solid was washed with a mixture of water and MeCN (water/MeCN = 1/2 vol/vol; 728 L). Above procedure was repeated for the other half. The obtained solid were combined and dried under reduced pressure at 50°C to afford 3-(*tert*-butyl)-6-(1*H*-pyrazol-1-yl)-1-(2,4,5-trifluorobenzyl)-1,3,5-triazine-2,4(1*H*,3*H*)-dione (**28**; 653.19 kg, 83.4%).

Physical state: white solid.

Melting point: 153°C

^{1}H NMR (400 MHz, CDCl$_3$): δ 8.29 (dd, J = 2.8, 0.6 Hz, 1H), 7.80 (d, J = 2.2 Hz, 1H), 7.04 (ddd, J = 10.3, 8.6, 6.9 Hz, 1H), 6.84 (ddd, J = 9.7, 9.7, 6.5 Hz, 1H), 6.46 (dd, J = 2.8, 1.6 Hz, 1H), 5.54 (s, 2H), 1.64 (s, 9H).

^{13}C{^{1}H} NMR (100 MHz, CDCl$_3$): δ 155.6 (ddd, $J_{C\text{-}F}$ = 245.0, 9.4, 2.8 Hz), 154.1, 150. 7, 149.9, 149.6 (ddd, $J_{C\text{-}F}$ = 251.0, 13.6, 13.6 Hz), 146.5 (ddd, $J_{C\text{-}F}$ = 244.0, 12.5, 3.7 Hz), 145.1, 132.6, 119.4 (ddd, $J_{C\text{-}F}$ = 16.0, 5.5, 4.5 Hz), 117.7 (ddd, $J_{C\text{-}F}$ = 20.0, 5.1, 1.5 Hz), 109.5, 105.6 (dd, $J_{C\text{-}F}$ = 27.5, 20.9 Hz), 61.5, 43.3 (d, $J_{C\text{-}F}$ = 3.0 Hz), 28.4.

HRMS-ESI (*m*/*z*): [M + Na]$^+$ calcd for $[C_{17}H_{16}F_3N_5O_2Na]^+$ 402.1154, found 402.1152.

Compound **29**, 6-(*meta*-tolyloxy)-1-(2,4,5-trifluorobenzyl)-1,3,5-triazine-2,4(1*H*,3*H*)-dione

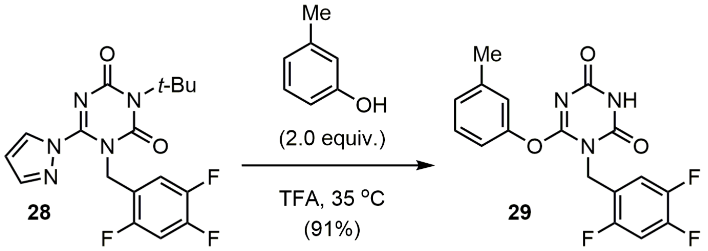

To a mixture of *meta*-cresol (104.3 kg, 964 mol) in TFA (681.6 kg, 2.5 vol) was added 3-(*tert*-butyl)-6-(1*H*-pyrazol-1-yl)-1-(2,4,5-trifluorobenzyl)-1,3,5-triazine-2,4(1*H*,3*H*)-dione (**28**; 183.0 kg, 482 mol). After stirring for 3 h at 35°C, EtOAc (2196.0 kg, 12.0 vol) was added to the resulting mixture at − 8°C to prepare the pre-quench solution. pH of the solution was adjusted to 6.0 with 9% NaOH aq., and the organic layer was separated. The organic layer was subsequently washed with 2% NaCl aq. and water. The organic layer was concentrated to 7.0 vol. *n*-Heptane (125.2 kg, 1.0 vol) and seed crystal were added to the concentrate at 40°C. *n*-Heptane (1502.1 kg, 12.0 vol) was added to the resulting slurry again. Then the resulting slurry was cooled to − 10°C and filtered. The filtered solid was washed with a mixture of EtOAc and *n*-heptane (EtOAc/*n*-heptane = 1/3 vol/vol; 1020 L). The obtained solid was dried under reduced pressure at 50°C to afford 6-(*meta*-tolyloxy)-1-(2,4,5-trifluorobenzyl)-1,3,5-triazine-2,4(1*H*,3*H*)-dione (**29**; 159.2 kg, 90.9%).

Physical state: white solid.

Melting point: 161°C

^{1}H NMR (400 MHz, $CDCl_3$): δ 7.35–7.26 (m, 2H), 7.10 (d, J = 7.4 Hz, 1H), 7.00 (ddd, J = 9.6, 9.6, 6.5 Hz, 1H), 6.87–6.84 (m, 2H), 5.22, (s, 2H), 2.37 (s, 3H).

^{13}C{^{1}H} NMR (100 MHz, $CDCl_3$): δ 159.8, 155.9 (ddd, $J_{C\text{-}F}$ = 246.0, 9.5, 2.2 Hz), 153.9, 150.4, 150.2, (ddd, $J_{C\text{-}F}$ = 253.0, 13.2, 13.2 Hz), 149.9, 146.9 (ddd, $J_{C\text{-}F}$ = 246.5, 12.5, 3.7 Hz), 140.3, 129.5, 128.0, 121.6, 118.5–118.2 (m), 118.4 (dd, $J_{C\text{-}F}$ = 2.3, 1.0 Hz), 117.9, 106.0 (dd, $J_{C\text{-}F}$ = 27.9, 21.3 Hz), 39.2 (d, $J_{C\text{-}F}$ = 2.9 Hz), 21.3.

HRMS-ESI (*m*/*z*): $[M + Na]^+$ calcd for $[C_{17}H_{12}F_3N_3O_3Na]^+$ 386.0728, found 386.0727.

Compound **30**, 3-((1-methyl-1*H*-1,2,4-triazol-3-yl)methyl)-6-(*meta*-tolyloxy)-1-(2,4,5-trifluorobenzyl)-1,3,5-triazine-2,4(1*H*,3*H*)-dione

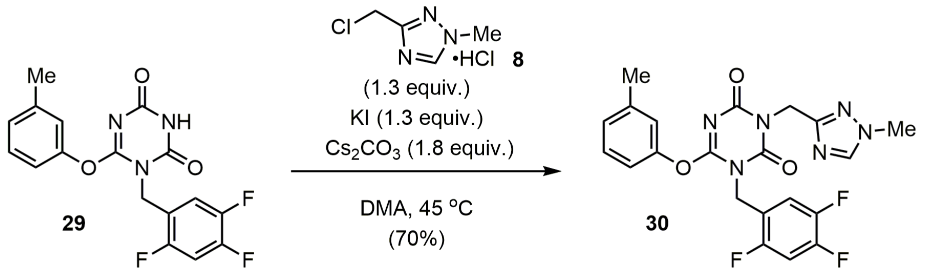

6-(*meta*-Tolyloxy)-1-(2,4,5-trifluorobenzyl)-1,3,5-triazine-2,4(1*H*,3*H*)-dione (**29**; 260.0 kg, 716 mol), 3-(chloromethyl)-1-methyl-1*H*-1,2,4-triazole·hydrochloride (**8**; 156.3 kg, 930 mol), DMA (1471.0 kg, 6.0 vol), KI (154.4 kg, 930 mol), and Cs_2CO_3 (419.7 kg, 1288 mol) were mixed. After stirring for 7.5 h at 45°C, AcOH (386.8 kg, 6441 mol) was added to the reaction mixture at 25°C, and the insoluble material was filtered. The insoluble material was washed with MeCN (1040 L, 4.0 vol). The obtained filtrates were combined and mixed with water (2080 L, 8.0 vol) and stirred at 25°C. After adding water (1560 L, 6.0 vol) to the resulting solution, and the mixture was cooled to 0°C and filtered. The obtained solid was washed with a mixture of MeCN (260 L, 1.0 vol) and water (1040 L, 4.0 vol). The obtained solid was dried under reduced pressure at 50°C to afford 3-((1-methyl-1*H*-1,2,4-triazol-3-yl)methyl)-6-(*meta*-tolyloxy)-1-(2,4,5-trifluorobenzyl)-1,3,5-triazine-2,4(1*H*,3*H*)-dione (**30**; 228.8 kg, 69.7%).

Physical state: white solid.

Melting point: 128°C

^{1}H NMR (400 MHz, $CDCl_3$): δ 7.93 (s, 1H), 7.36 (ddd, *J* = 10.3, 8.6, 6.9 Hz, 1H), 7.25 (dd, *J* = 8.0, 8.0 Hz, 1H), 7.08 (d, *J* = 7.4 Hz, 1H), 6.98 (ddd, *J* = 9.6, 9.6, 6.5 Hz, 1H), 6.84–6.87 (m, 2H), 5.27 (s, 2H), 5.22 (s, 2H), 3.84 (s, 3H), 2.35 (s, 3H).

^{13}C{^{1}H} NMR (100 MHz, $CDCl_3$): δ 159.3, 158.2, 155.7 (ddd, $J_{C\text{-}F}$ = 246.0, 9.4, 2.8 Hz), 153.1, 150.9, 150.3, 150.1 (ddd, $J_{C\text{-}F}$ = 352.0, 14.3, 12.1 Hz), 146.9 (ddd, $J_{C\text{-}F}$ = 245.8, 13.2, 3.7 Hz), 144.2, 140.1, 129.4, 127.8, 121.6, 118.6 (ddd, $J_{C\text{-}F}$ = 16.0, 5.2, 5.2 Hz), 118.0, 117.9 (ddd, $J_{C\text{-}F}$ = 22.0, 5.1, 1.5 Hz), 105.8, (dd, $J_{C\text{-}F}$ = 27.9, 21.3 Hz), 40.1, 39.8 (d, $J_{C\text{-}F}$ = 3.7 Hz), 36.1, 21.2.

HRMS-ESI (*m*/*z*): $[M + H]^+$ calcd for $[C_{21}H_{18}F_3N_6O_3]^+$ 459.1392, found 459.1394.

Compound **1**, (6*E*)-6-[(6-Chloro-2-methyl-2*H*-indazol-5-yl)imino]-3-[(1-methyl-1*H*-1,2,4-triazol-3-yl)methyl]-1-(2,4,5-trifluorobenzyl)-1,3,5-triazinane-2,4-dione (Ensitrelvir)

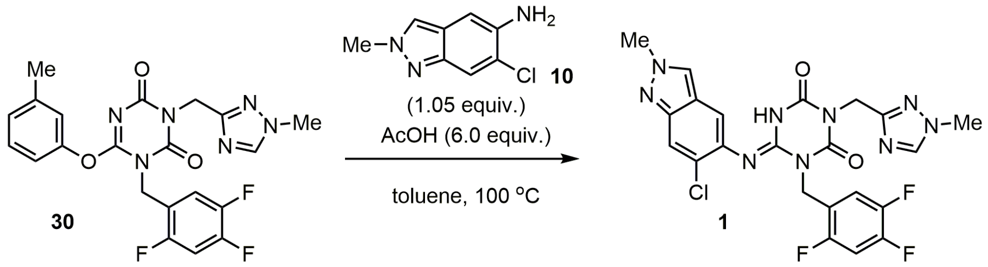

3-((1-Methyl-1*H*-1,2,4-triazol-3-yl)methyl)-6-(*meta*-tolyloxy)-1-(2,4,5-trifluorobenzyl)-1,3,5-triazine-2,4(1*H*,3*H*)-dione (**30**; 195.0 kg, 425 mol), 6-chloro-2-methyl-2*H*-indazol-5-amine (**10**; 81.0 kg, 446 mol), AcOH (153.3 kg, 2553 mol) and toluene (1171 L, 6.0 vol) were mixed, and stirred at 100°C for 8 h. After toluene (1950 L, 10.0 vol) was added, the mixture was cooled to 25°C and filtered. The filtered solid was washed with toluene (975 L, 5.0 vol) and acetone (585 L, 3.0 vol). The obtained wet solid of Ensitrelvir (**1**; 225.4 kg) was used in next step without drying.

Physical state: white solid.

Melting point: 234°C

^{1}H NMR (400 MHz, $CDCl_3$): δ 7.94 (s, 1H), 7.80 (d, J = 3.1 Hz, 1H), 7.56 (s, 1H), 7.44–7.37 (m, 1H), 7.07 (s, 1H), 6.92 (ddd, J = 9.6, 9.6, 6.6 Hz, 1H), 5.35 (s, 2H), 5.15 (s, 2H), 4.20 (s, 3H), 3.88 (s, 3H).

^{13}C{^{1}H} NMR (100 MHz, THF-d_8): δ 160.6, 156.7 (ddd, $J_{C\text{-}F}$ = 244.0, 9.5, 2.6 Hz), 151.3–148.6 (m), 150.8, 148.7, 147.9 (ddd, $J_{C\text{-}F}$ = 241.0, 12.5, 3.7 Hz), 147.8, 145.5, 140.2, 138.0, 128.6, 123.9, 122.6, 122.7–122.5 (m), 118.6, 117.5 (dd, $J_{C\text{-}F}$ = 20.5, 5.1 Hz), 111.6, 106.1 (dd, $J_{C\text{-}F}$ = 28.6, 21.3 Hz), 40.3, 40.0, 39.9 (d, $J_{C\text{-}F}$ = 7.0 Hz), 35.7.

HRMS-ESI (*m*/*z*): $[M + H]^+$ calcd for $[C_{22}H_{18}ClF_3N_9O_2]^+$ 532.1224, found 532.1227.

Compound **API**, (6*E*)-6-[(6-Chloro-2-methyl-2*H*-indazol-5-yl)imino]-3-[(1-methyl-1*H*-1,2,4-triazol-3-yl)methyl]-1-(2,4,5-trifluorobenzyl)-1,3,5-triazinane-2,4-dione·fumaric acid (Ensitrelvir·fumaric acid)

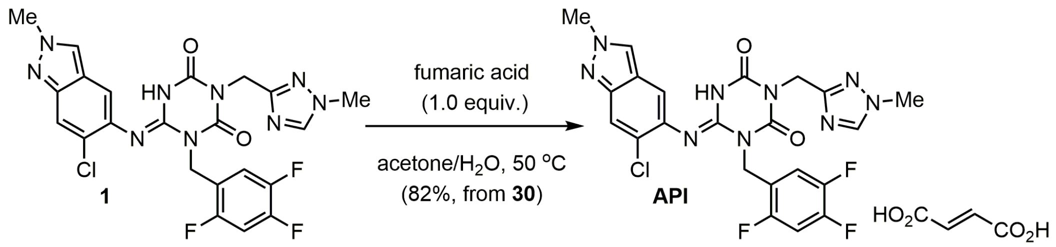

The molar equivalent, vol and weights in this step were based on the amount of compound **30** used.

Wet Ensitrelvir (**1**; 225.4 kg), acetone (7626 L, 39.1 vol), and water (1150 L, 5.9 vol) were mixed and stirred at 50°C for treatment with activated carbon (19.6 kg, 0.1 weights). After stirring for 0.5 h, the activated carbon was removed and the filtrates were concentrated to 36.0 volumes and mixed with fumaric acid (48.9 kg, 421 mol), acetone (1112 L, 5.7 vol) and water (59 L, 0.3 vol). The mixture was concentrated to 14.0 volumes. Acetone (390 L, 2.0 vol) was added to the slurry and stirred at 55°C for 2 h. The resulting slurry was cooled to 0°C and filtered. The obtained solid were washed with acetone (2151.2 kg, 14.0 vol). The obtained crystals were dried under reduced pressure at 50°C to afford **API** (224.8 kg, 81.6% for 2 steps).

Physical state: white solid.

Melting point: 245°C

^{1}H NMR (400 MHz, pyridine-*d_5*): δ 12.08 (brs, 2H), 8.28 (s, 1H), 7.93–7.86 (m, 1H), 7.86 (s, 1H), 7.78 (s, 1H), 7.40 (s, 2H), 7.24 (s, 1H), 7.21–7.13 (m, 1H), 5.59 (s, 2H), 5.53 (s, 2H), 3.98 (s, 3H), 3.63 (s, 3H).

^{13}C{^{1}H} NMR (100 MHz, THF-*d_8*): δ 166.5, 160.7, 156.7 (ddd, $J_{C\text{-}F}$ = 243.0, 9.9, 2.6 Hz), 151.4–148.7 (m), 150.9, 148.0 (ddd, $J_{C\text{-}F}$ = 241.0, 12.5, 3.7 Hz), 148.0, 145.6, 140.3, 138.1, 134.9, 128.7, 124.0, 122.8, 122.7–122.6 (m),118.8, 117.6 (dd, $J_{C\text{-}F}$ = 20.5, 5.1 Hz), 111.6, 106.2 (dd, $J_{C\text{-}F}$ = 27.9, 21.3 Hz), 40.4, 40.1, 40.0 (d, $J_{C\text{-}F}$ = 5.0 Hz), 36.1.

HRMS-ESI (*m*/*z*): $[M + H]^+$ calcd for $[C_{22}H_{18}ClF_3N_9O_2]^+$ 532.1224, found 532.1221.

According to the single crystal X-Ray analysis, the bond length in one fumaric acid (C23–O38; 1.31Å *vs*. C23–O39; 1.20Å) was non-equivalent. Also, the bond length in another fumaric acid (C26–O41; 1.30Å *vs*. C26–O43; 1.19Å) was non-equivalent.[1)] Therefore, Ensitrelvir **1** forms a co-crystal with fumaric acid, not a salt. (Each position number was referred to the ORTEP in the reference 1)

Characterization of other compounds

Compound **22**, (4-chloro-2-fluoro-5-nitrobenzylidene)hydrazine.

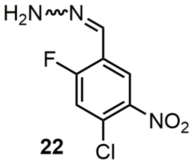

22

Physical state: yellow solid.

^{1}H NMR (400 MHz, DMSO-*d*$_6$): δ 8.24 (d, J = 7.1 Hz, 1H), 7.71 (s, 1H), 7.64–7.60 (m, 3H).

^{13}C{^{1}H} NMR (100 MHz, DMSO-*d*$_6$): δ 159.2 (d, $J_{C\text{-}F}$ = 257.5 Hz), 144.1 (d, $J_{C\text{-}F}$ = 2.9 Hz), 125.7 (d, $J_{C\text{-}F}$ = 2.9 Hz), 125.0 (d, $J_{C\text{-}F}$ = 12.5 Hz), 123.9 (d, $J_{C\text{-}F}$ = 11.7 Hz), 121.9 (d, $J_{C\text{-}F}$ = 6.6 Hz), 119.3 (d, $J_{C\text{-}F}$ = 27.1 Hz).

HRMS-ESI (*m*/*z*): $[M + H]^+$ calcd for $[C_7H_6ClFN_3O_2]^+$ 218.0133, found 218.0135.

Compound **23**, 1,2-bis(4-chloro-2-fluoro-5-nitrobenzylidene)hydrazine.

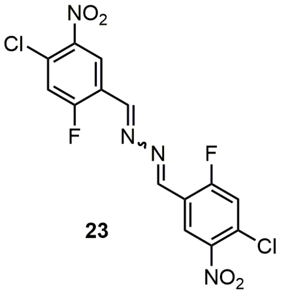

23

Physical state: yellow solid.

^{1}H NMR (400 MHz, DMSO-*d*$_6$): δ 8.79 (s, 2H), 8.70 (d, J = 6.6 Hz, 2H), 8.06 (d, J = 10.0 Hz, 2H).

^{13}C{^{1}H} NMR (100 MHz, DMSO-*d*$_6$): δ 162.4 (d, $J_{C\text{-}F}$ = 264.8 Hz), 154.2, 144.8 (d, $J_{C\text{-}F}$ = 2.9 Hz), 130.5 (d, $J_{C\text{-}F}$ = 13.0 Hz), 126.2 (d, $J_{C\text{-}F}$ = 5.0 Hz), 121.9 (d, $J_{C\text{-}F}$ = 12.5 Hz), 121.0 (d, $J_{C\text{-}F}$ = 26.4 Hz).

HRMS-ESI (*m*/*z*): $[M + Na]^+$ calcd for $[C_{14}H_6Cl_2F_2N_4O_4Na]^+$ 424.9632, found 424.9635.

Compound **24**, 6-hydrazineyl-5-nitro-2*H*-indazole.

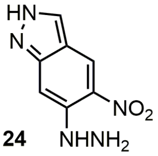

24

Physical state: red solid.

^{1}H NMR (400 MHz, DMSO-d_6): δ 12.87 (brs, 1H), 8.63 (s, 1H), 8.42 (s, 1H), 8.08 (d, J = 1.0 Hz, 1H), 7.31 (s, 1H), 4.53 (brs, 2H).

^{13}C{^{1}H} NMR (100 MHz, DMSO-d_6): δ 146.4, 143.8, 136.5, 129.9, 121.2, 115.4, 89.4.

HRMS-ESI (*m*/*z*): $[M + H]^+$ calcd for $[C_7H_8N_5O_2]^+$ 194.0678, found 194.0676.

Compound **31**, 3-((1-methyl-1*H*-1,2,4-triazol-3-yl)methyl)-6-(1*H*-pyrazol-1-yl)-1-(2,4,5-trifluorobenzyl)-1,3,5-triazine-2,4(1*H*,3*H*)-dione

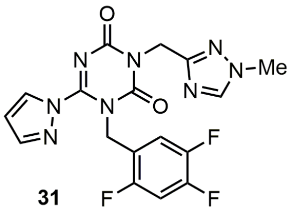

31

Physical state: white solid.

^{1}H NMR (400 MHz, CDCl$_3$): δ 8.42 (dd, J = 2.8, 0.6 Hz, 1H), 7.93 (s, 1H), 7.81–7.80 (m, 1H), 7.16 (m, 1H), 6.87 (ddd, J = 9.7, 9.7, 9.7 Hz, 1H), 6.48 (dd, J = 2.9, 1.7 Hz, 1H), 5.83 (s, 2H), 5.25 (s, 2H), 3.84 (s, 3H).

^{13}C{^{1}H} NMR (100 MHz, CDCl$_3$): δ 158.9, 155.4 (ddd, $J_{C\text{-}F}$ = 245.0, 9.5, 2.9 Hz), 153.0, 151.3, 149.5 (ddd, $J_{C\text{-}F}$ = 250.0, 13.9, 12.8 Hz), 146.7 (ddd, $J_{C\text{-}F}$ = 243.5, 12.5, 3.7 Hz), 145.4, 145.3, 144.3, 133.4, 119.4 (ddd, $J_{C\text{-}F}$ = 16.1, 4.8, 4.8 Hz), 117.2 (dd, $J_{C\text{-}F}$ = 20.5, 5.1 Hz), 109.7, 105.6 (dd, $J_{C\text{-}F}$ = 27.1, 21.3 Hz), 44.2 (d, $J_{C\text{-}F}$ = 2.9 Hz), 40.4, 36.1.

HRMS-ESI (*m*/*z*): $[M + H]^+$ calcd for $[C_{17}H_{14}F_3N_8O_2]^+$ 419.1192, found 419.1193.

^{1}H and ^{13}C NMR spectra of synthesized compounds.

^{1}H NMR of compound **13**.

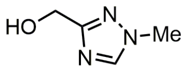

13 (DMSO-d_6, 400 MHz)

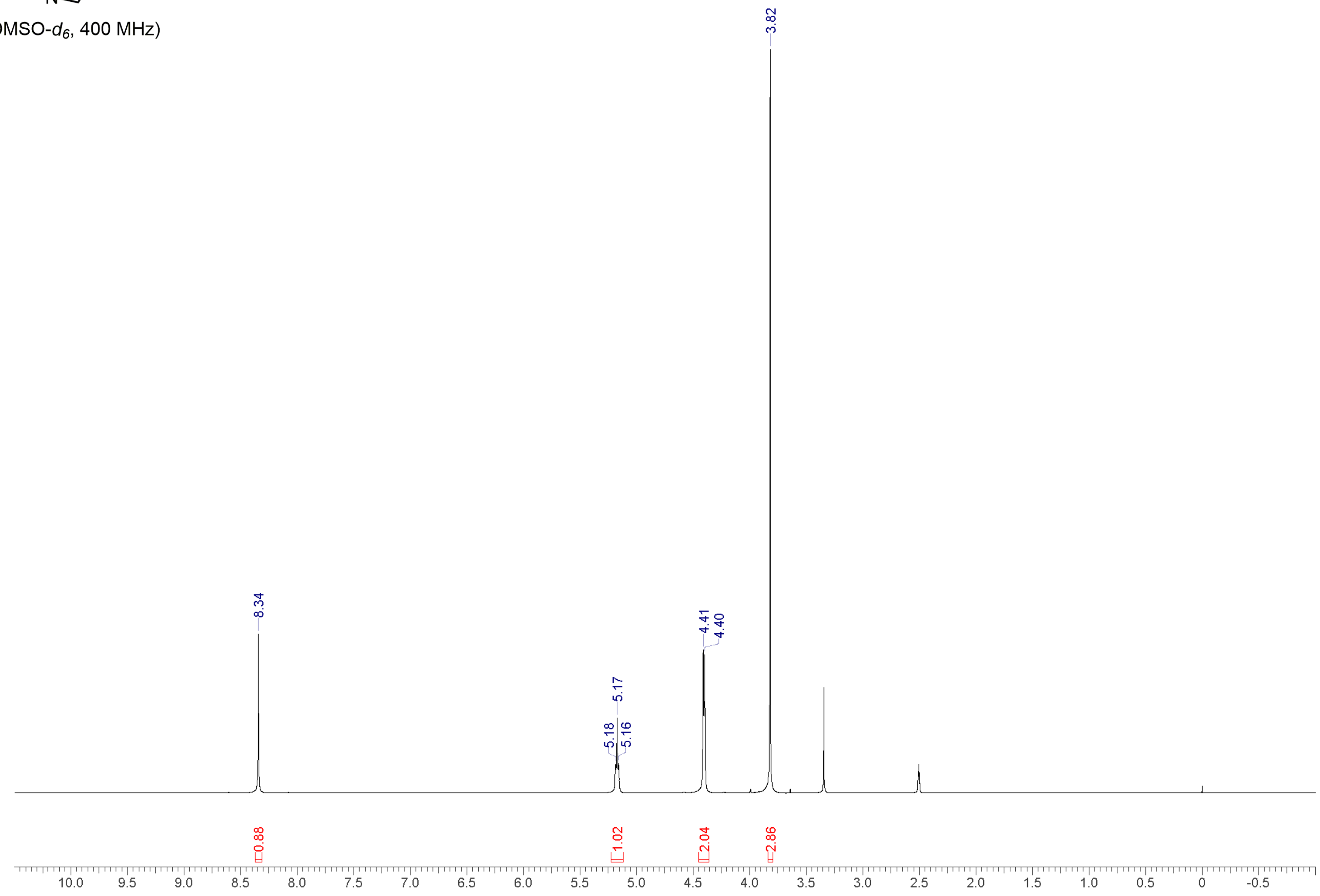

^{13}C NMR of compound **13**.

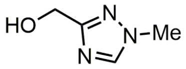

13 (DMSO-d_6, 100 MHz)

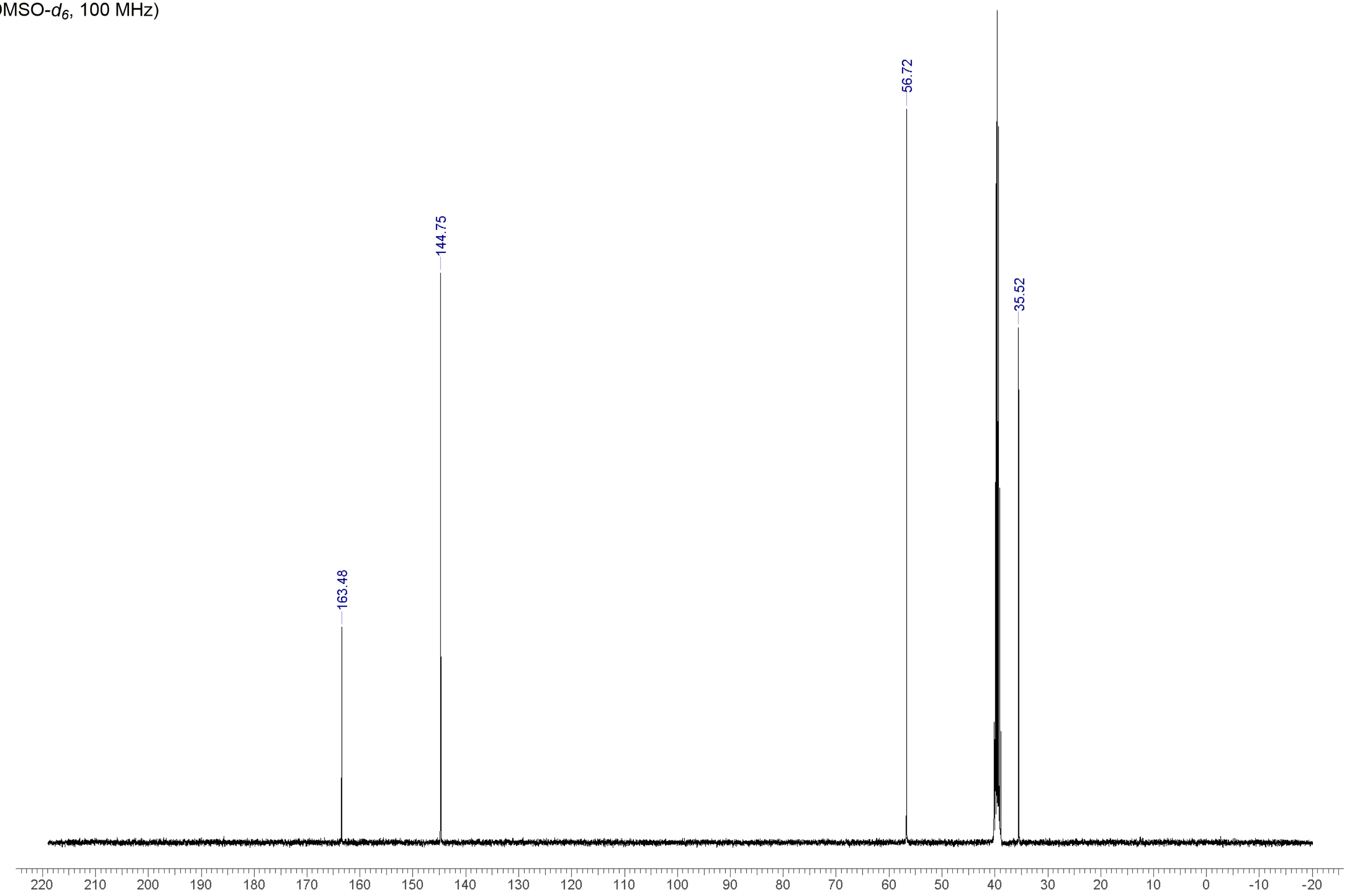

^{1}H NMR of compound **8**.

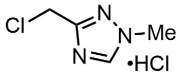

8 (DMSO-d_6, 400 MHz)

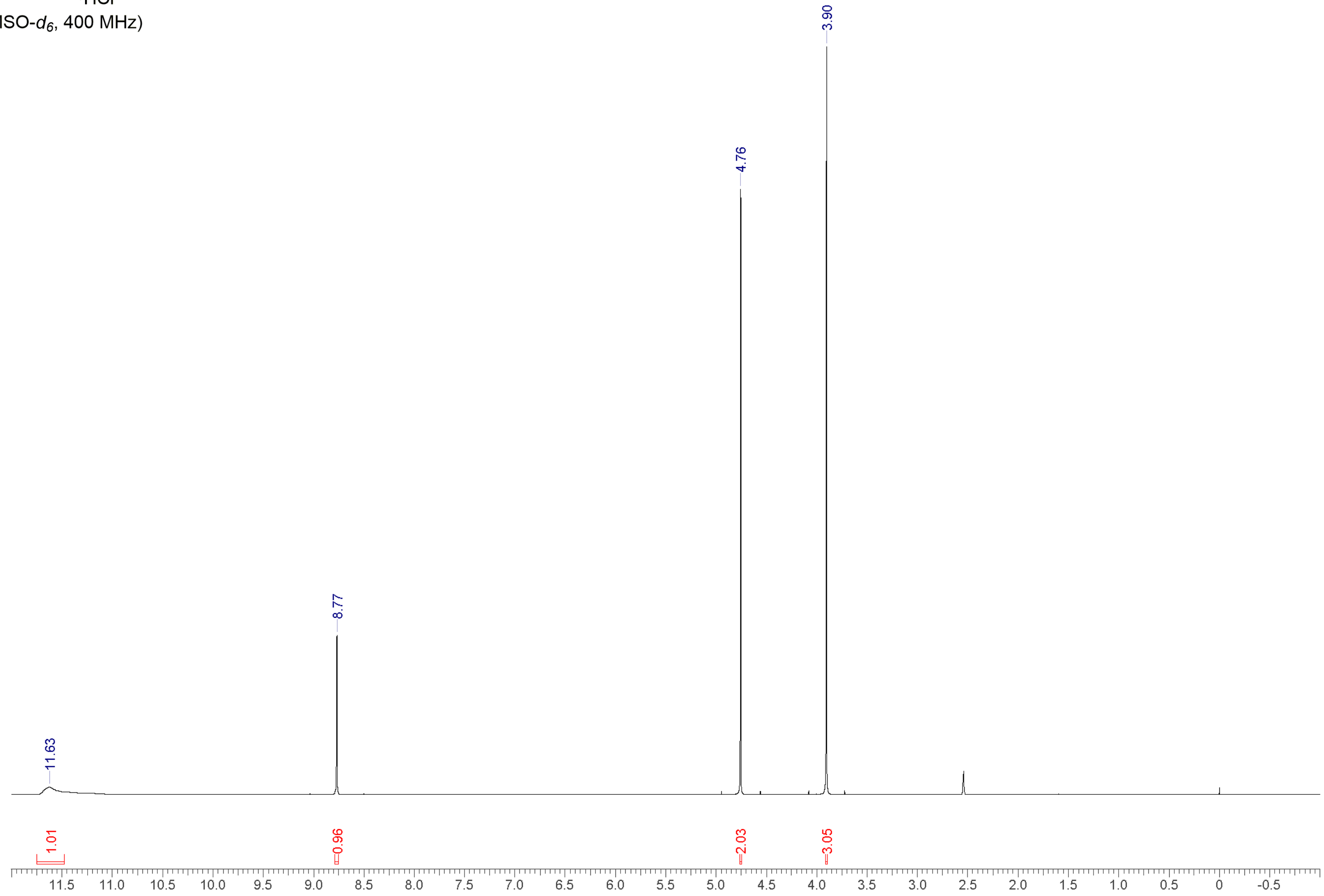

^{13}C NMR of compound **8**.

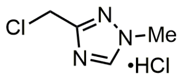

8 (DMSO-d_6, 100 MHz)

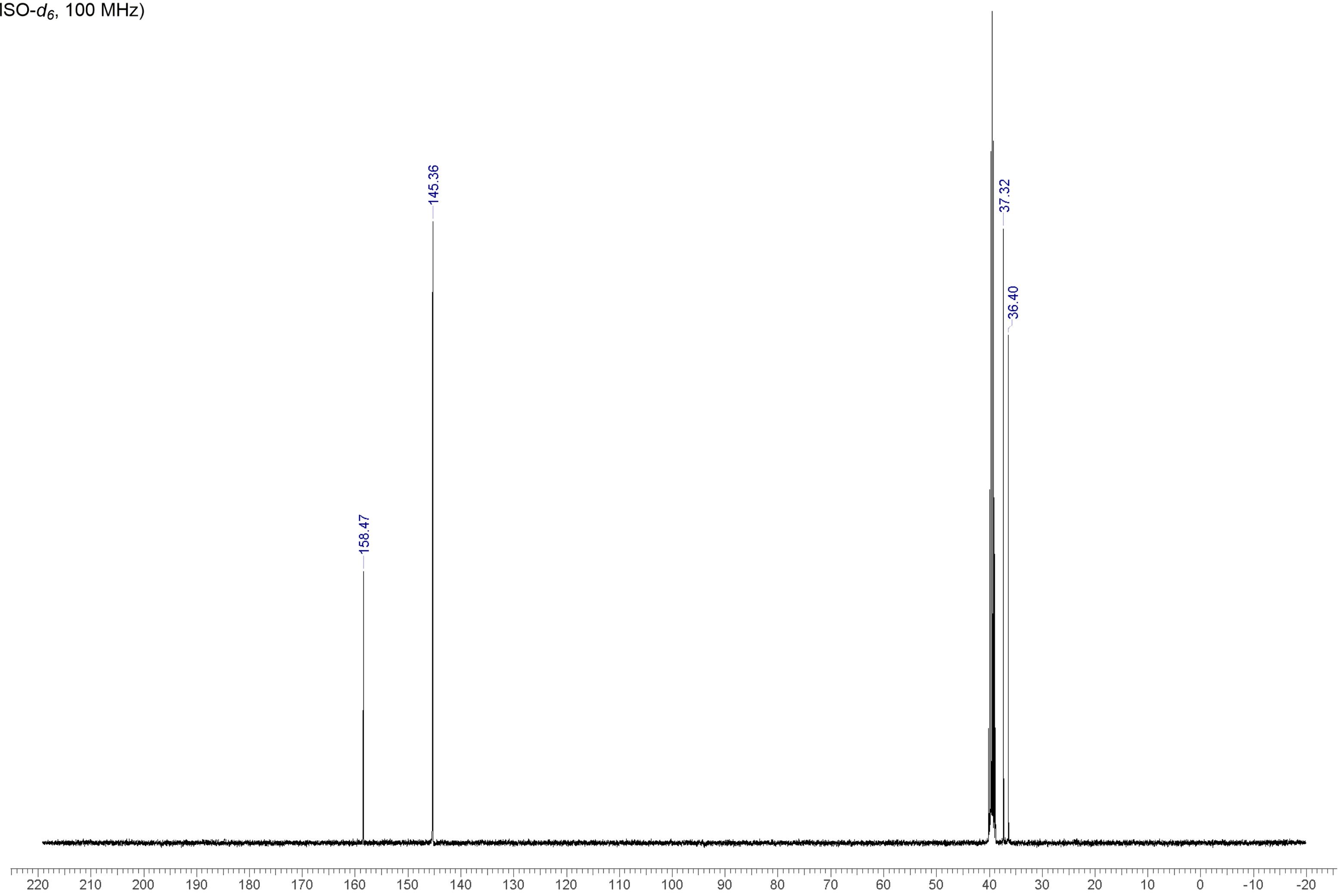

^{1}H NMR of compound **15**.

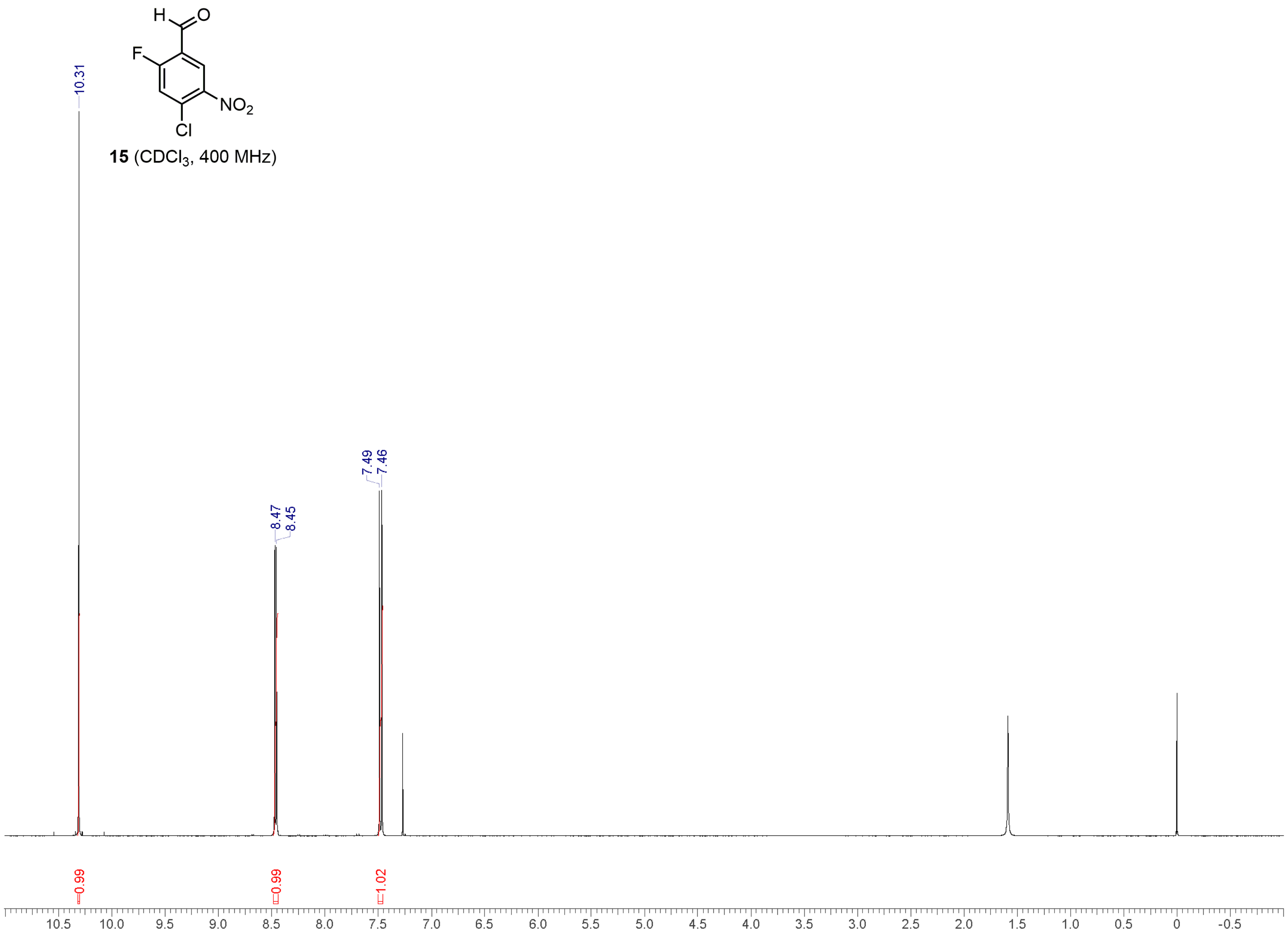

^{13}C NMR of compound **15**.

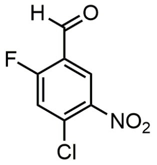

15 ($CDCl_3$, 100 MHz)

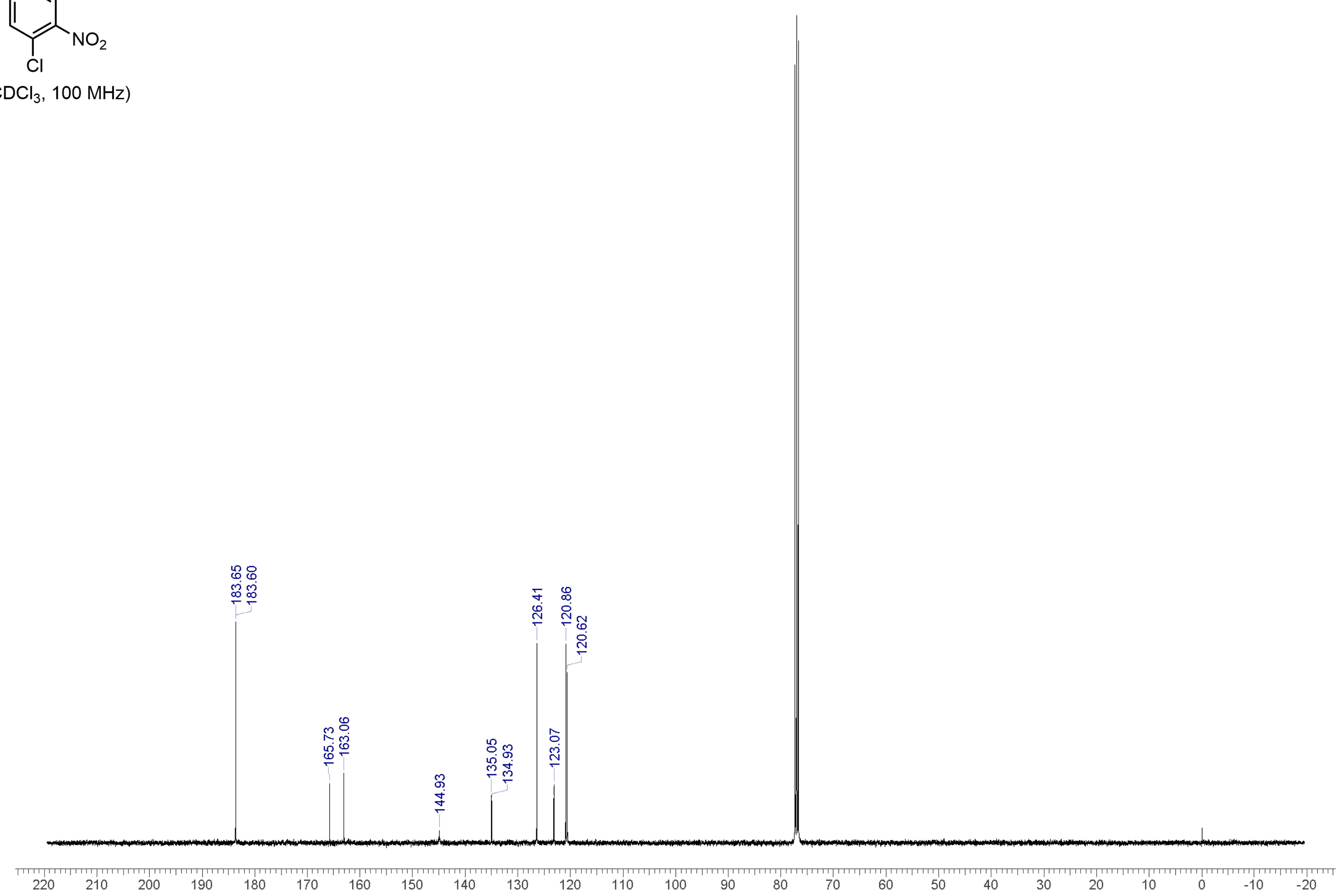

^{1}H NMR of compound **16**.

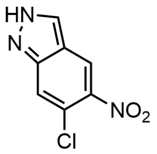

16 ($CDCl_3$, 400 MHz)

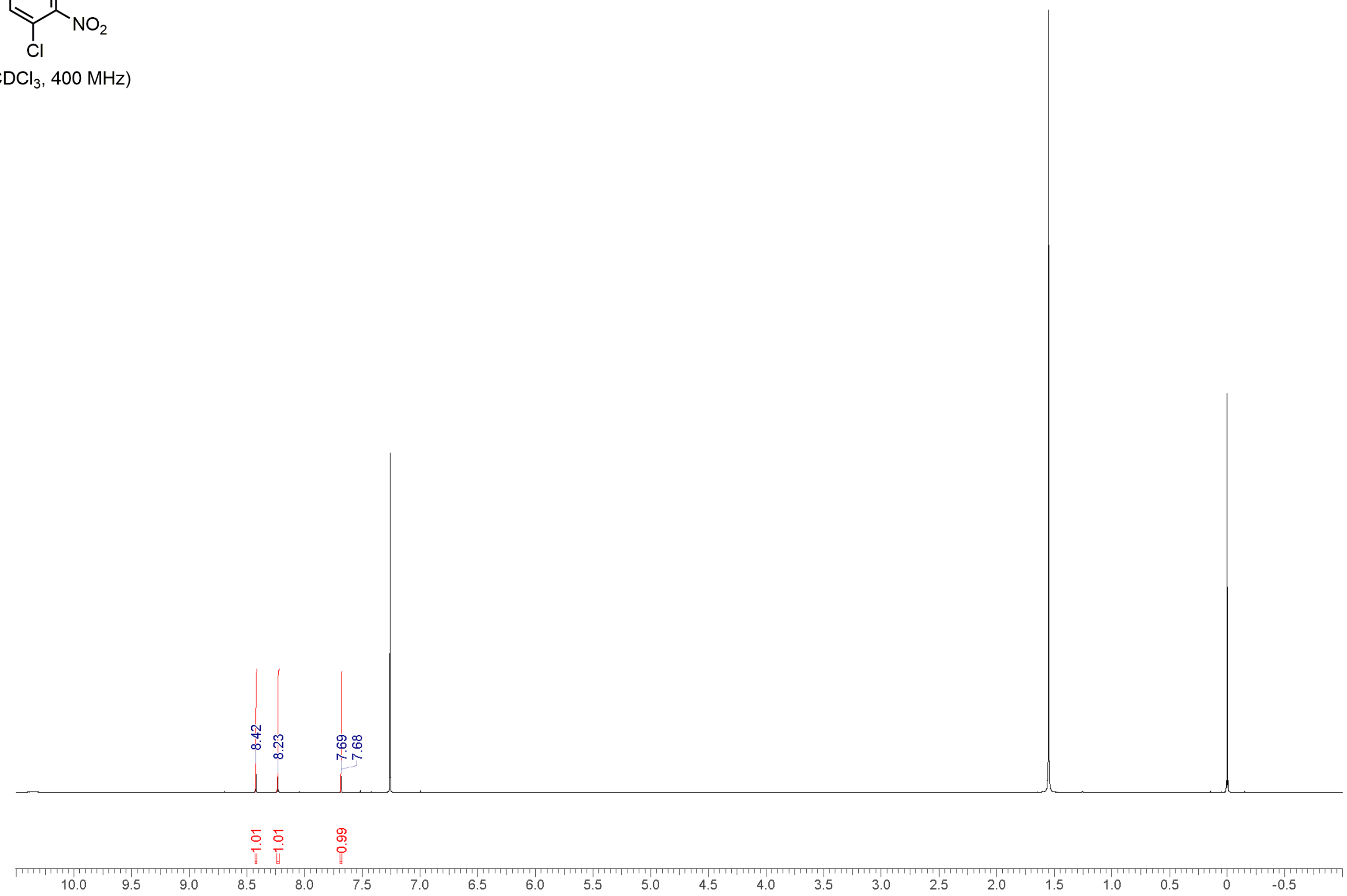

^{13}C NMR of compound **16**.

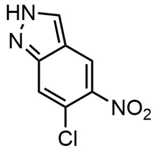

16 (DMSO-d_6, 100 MHz)

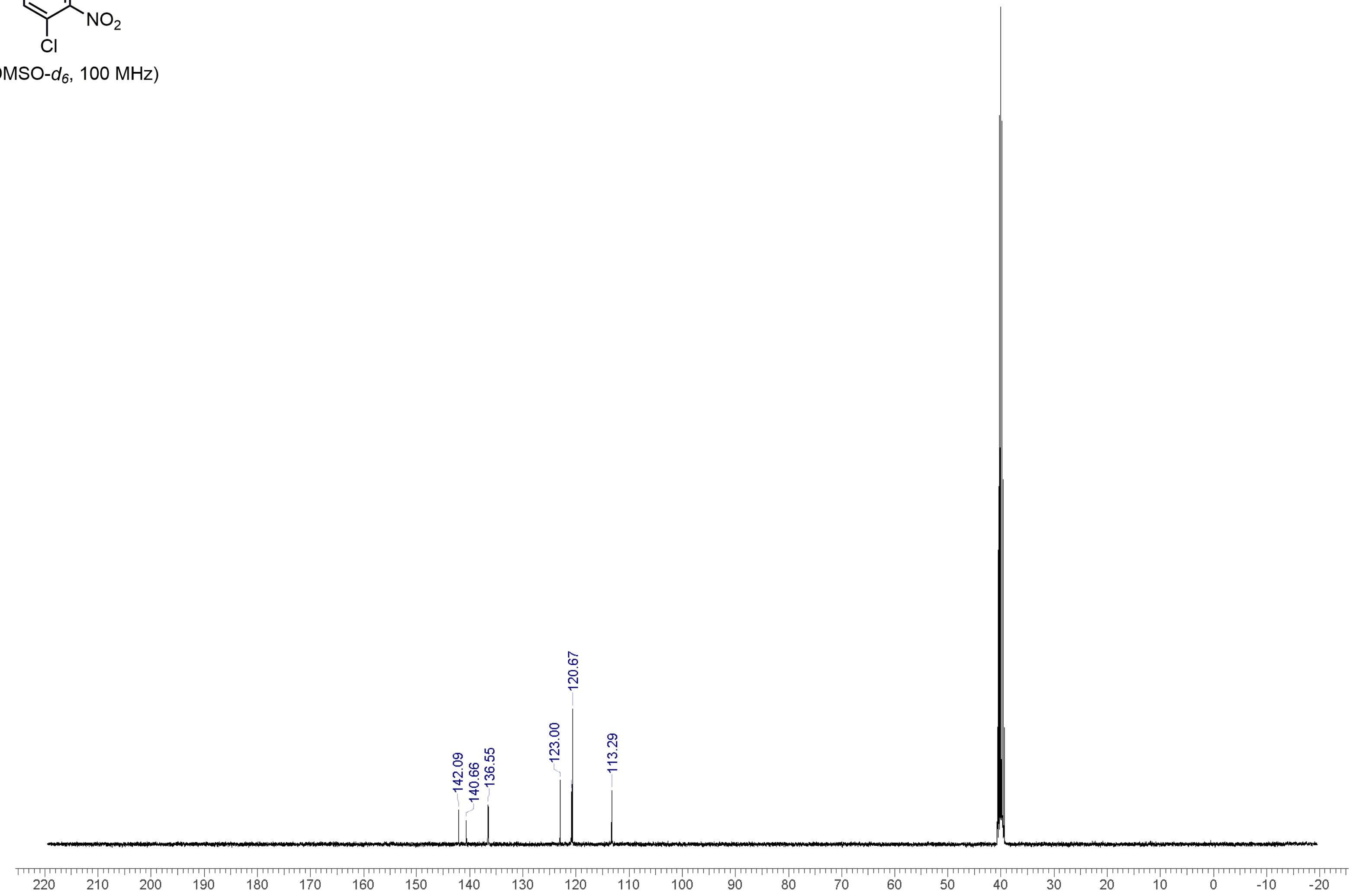

^{1}H NMR of compound **17**.

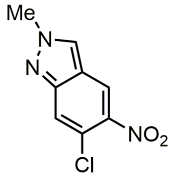

17 ($CDCl_3$, 400 MHz)

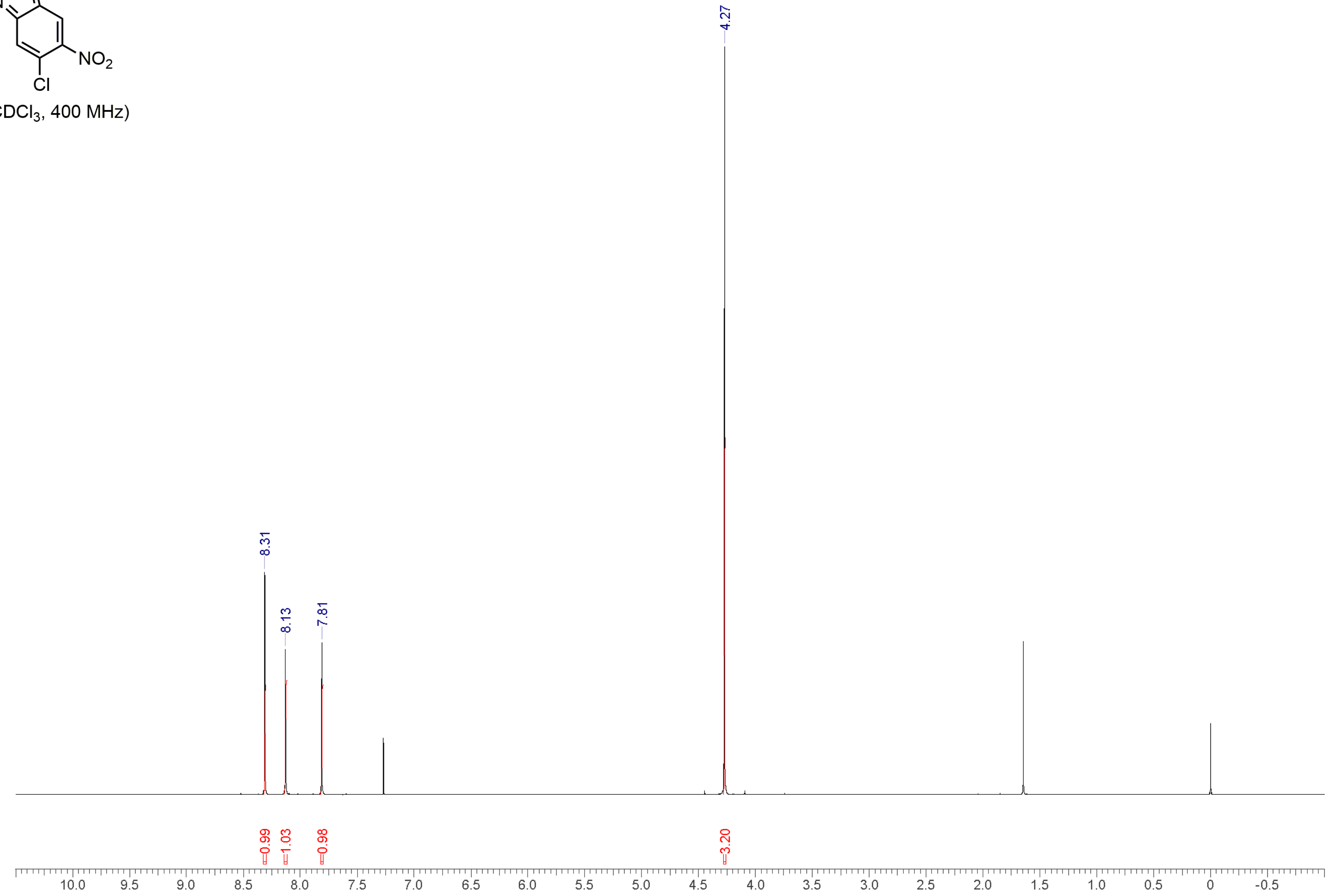

^{13}C NMR of compound **17**.

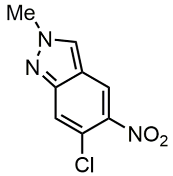

17 ($CDCl_3$, 100 MHz)

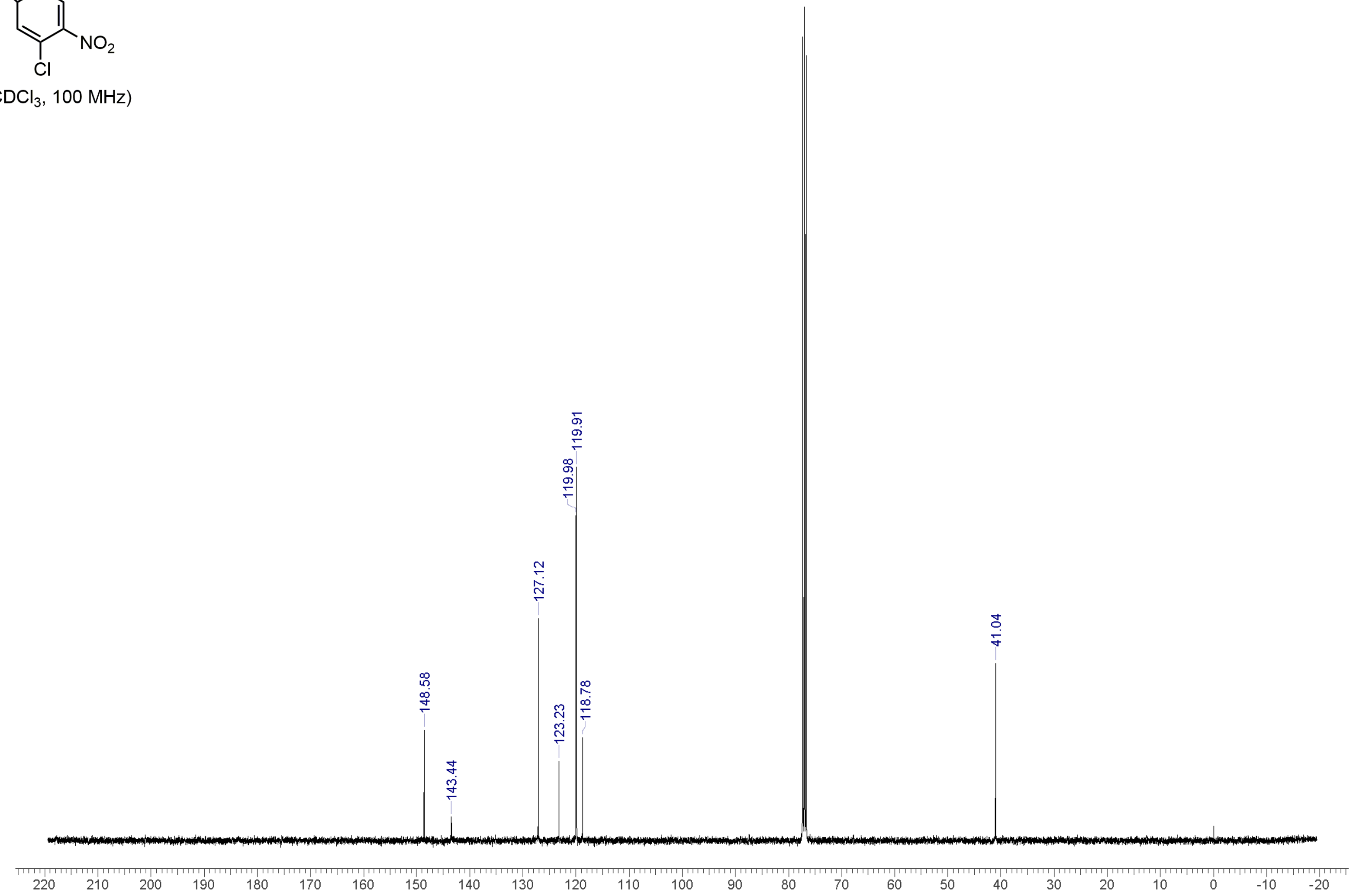

^{1}H NMR of compound **10**.

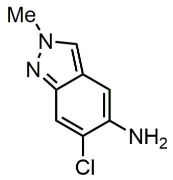

10 ($CDCl_3$, 400MHz)

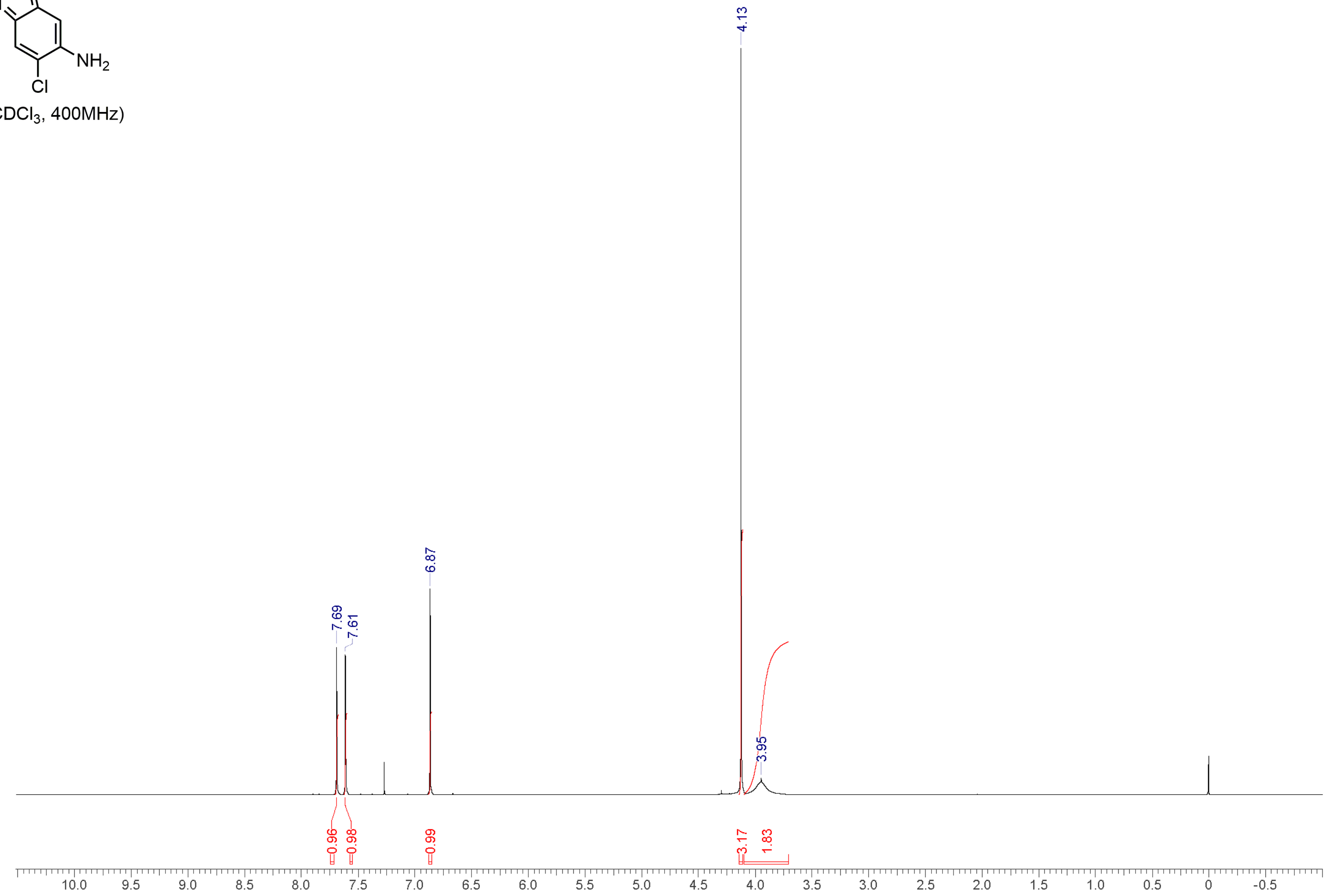

^{13}C NMR of compound **10**.

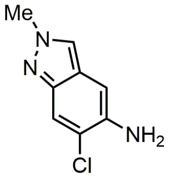

10 ($CDCl_3$, 100MHz)

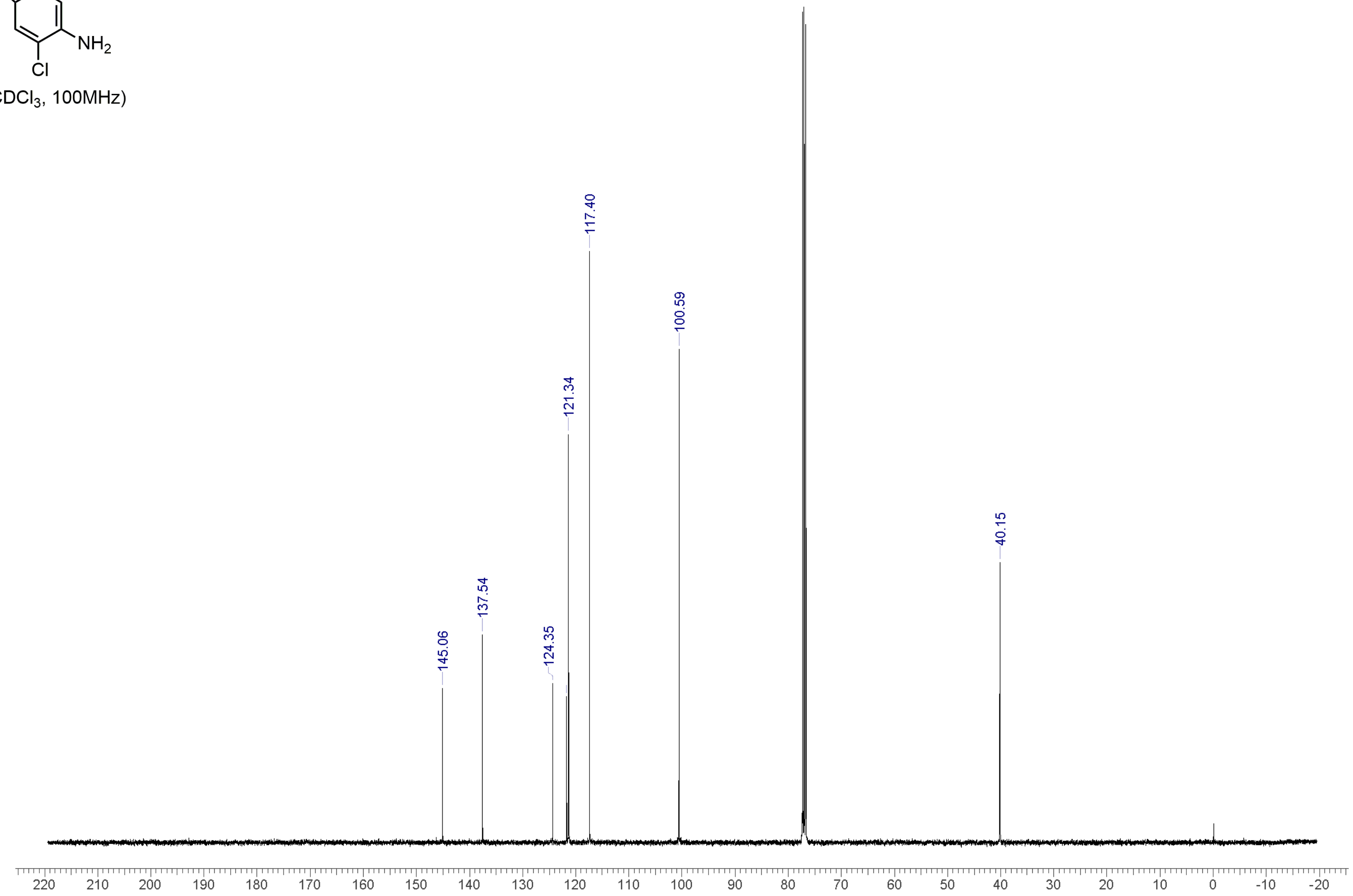

^{1}H NMR of compound **27**.

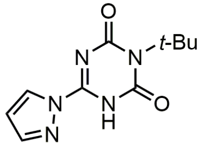

27 (CDCl$_3$, 400 MHz)

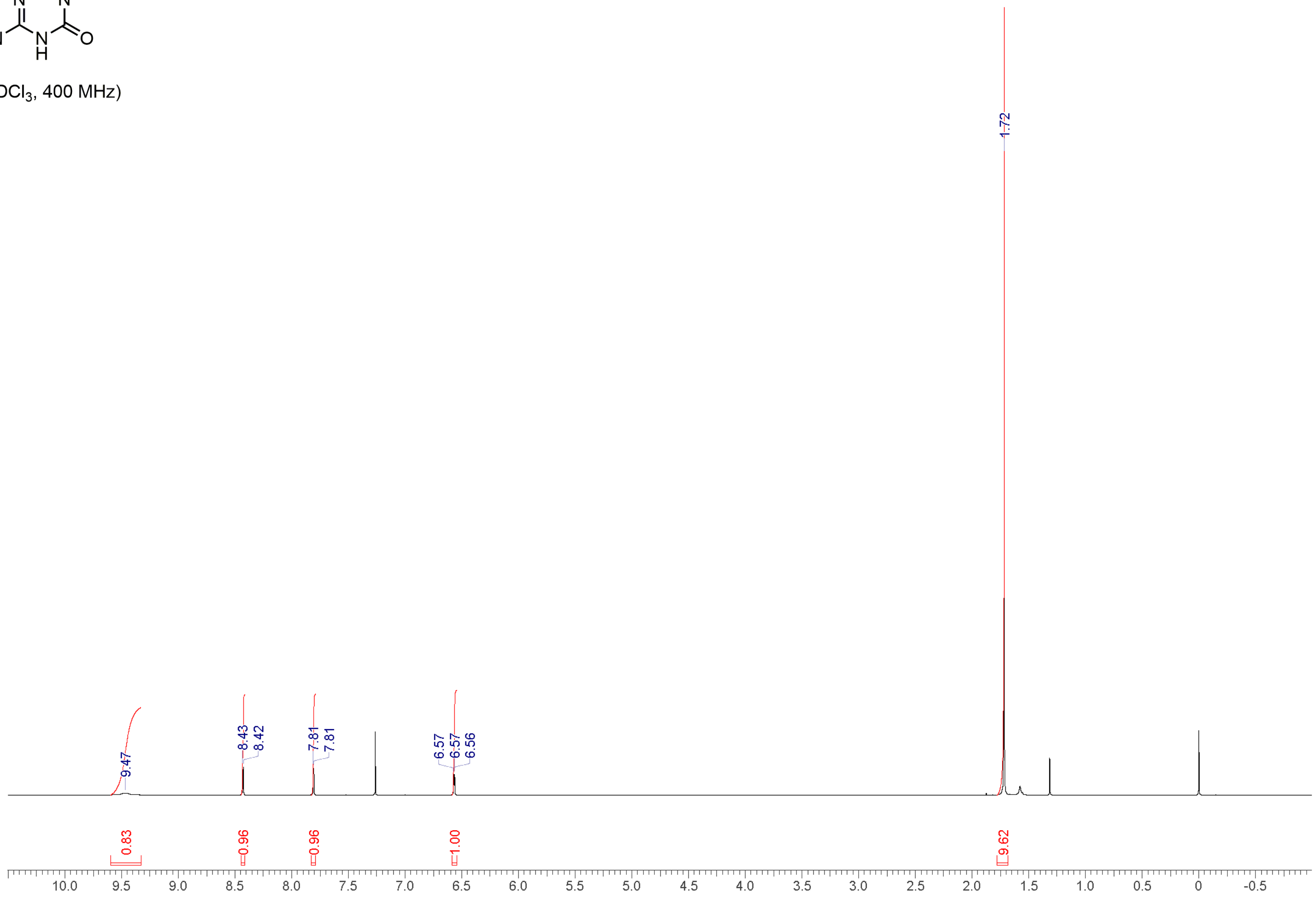

^{13}C NMR of compound **27**.

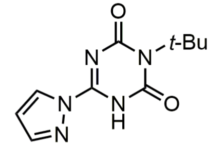

27 ($CDCl_3$, 100 MHz)

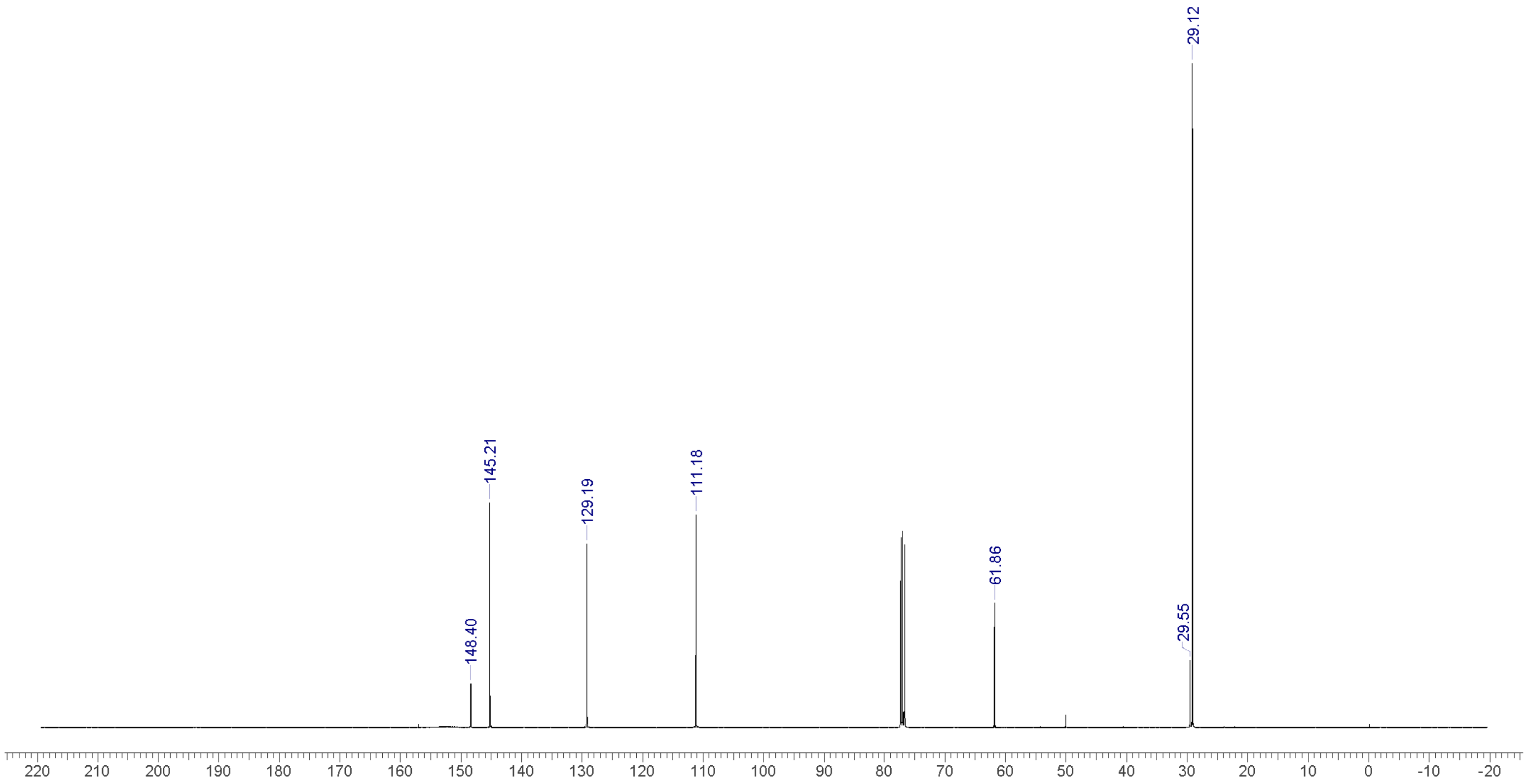

^{1}H NMR of compound **28**.

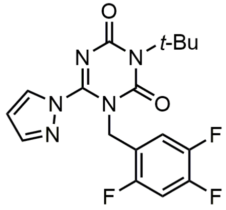

28 ($CDCl_3$, 400 MHz)

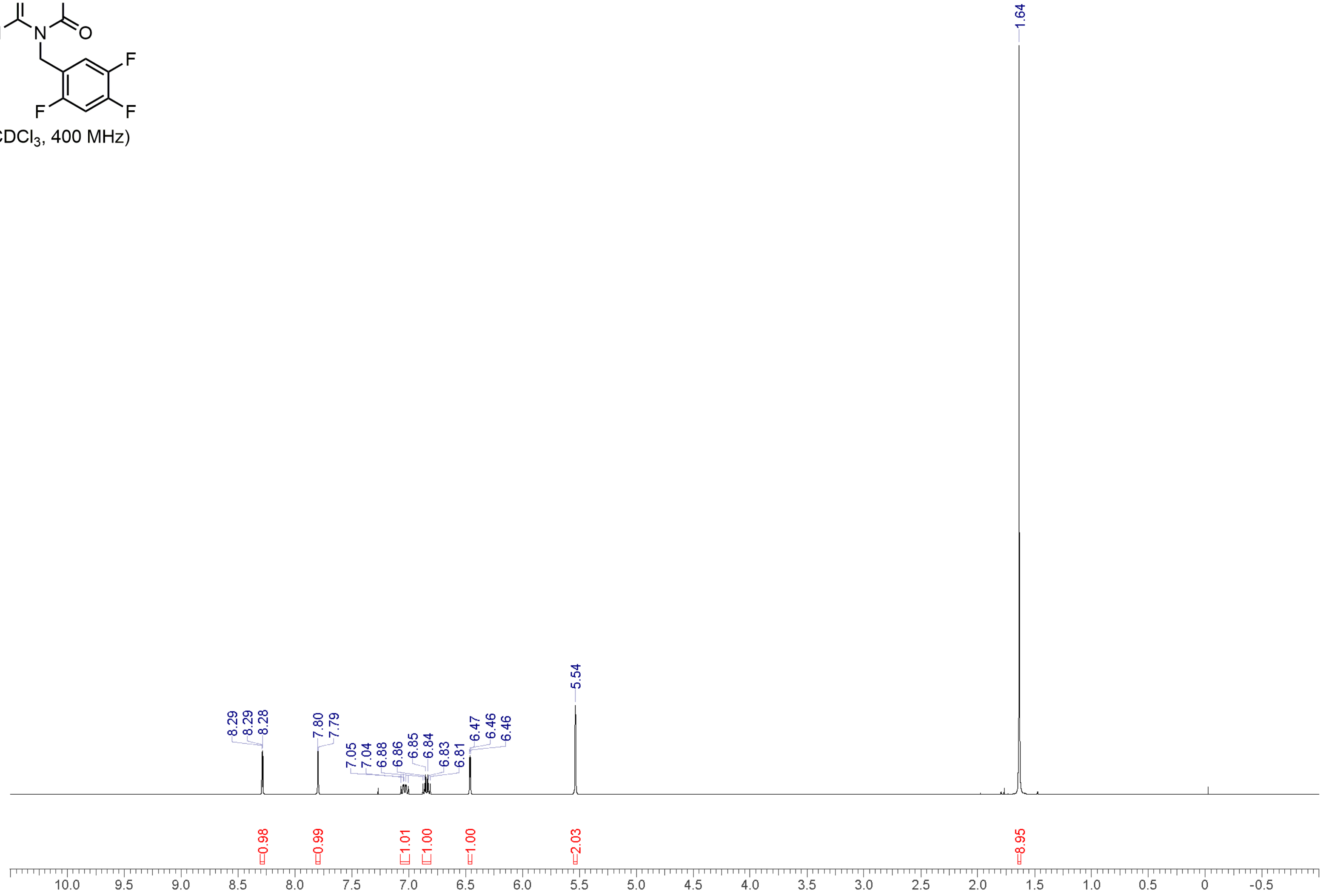

^{13}C NMR of compound **28**.

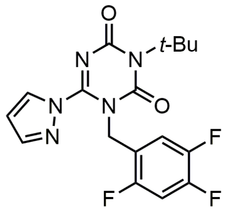

28 ($CDCl_3$, 100 MHz)

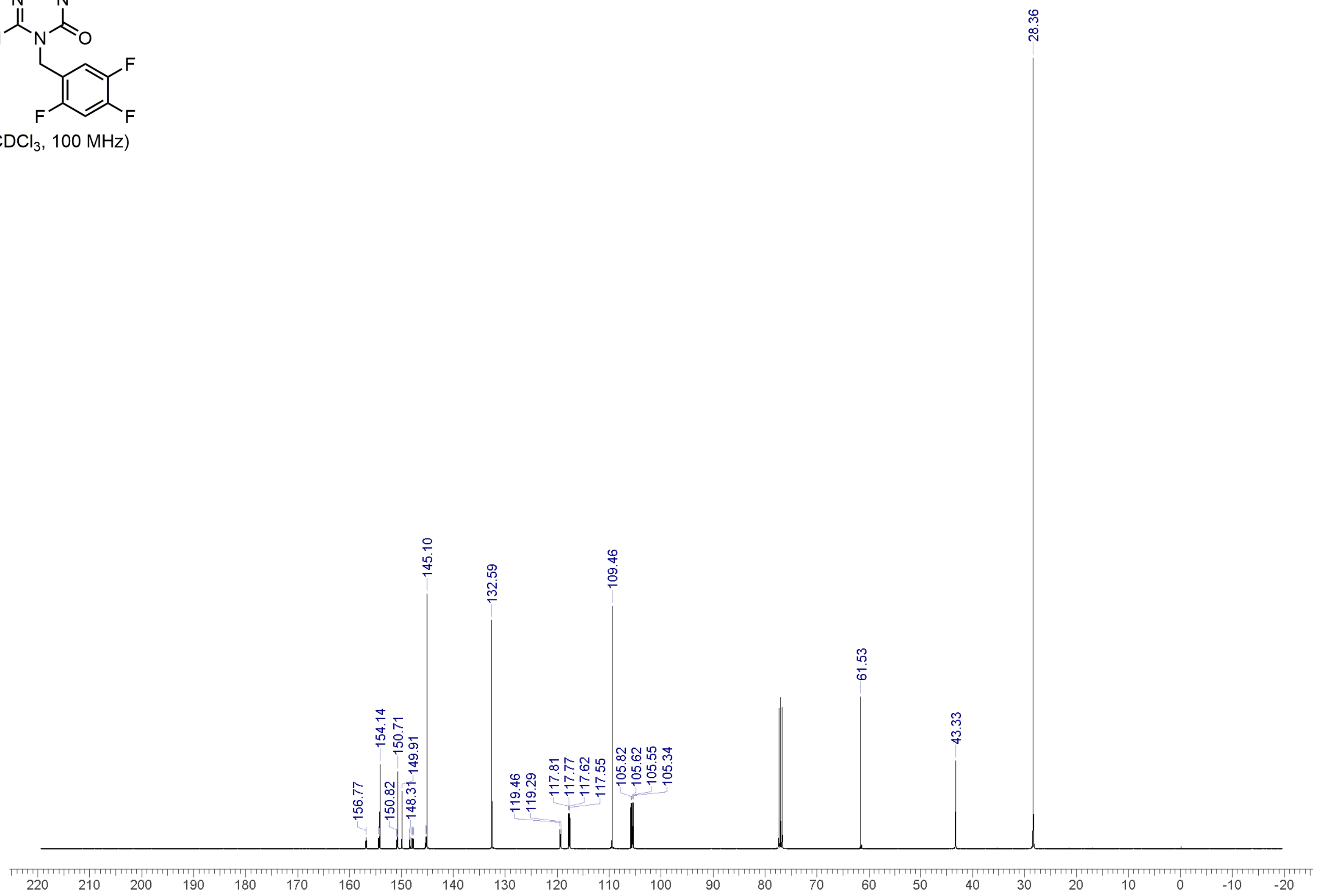

^{1}H NMR of compound **29**.

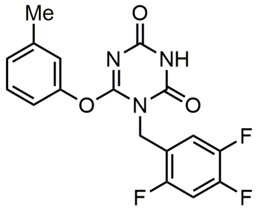
29 (CDCl$_3$, 400 MHz)

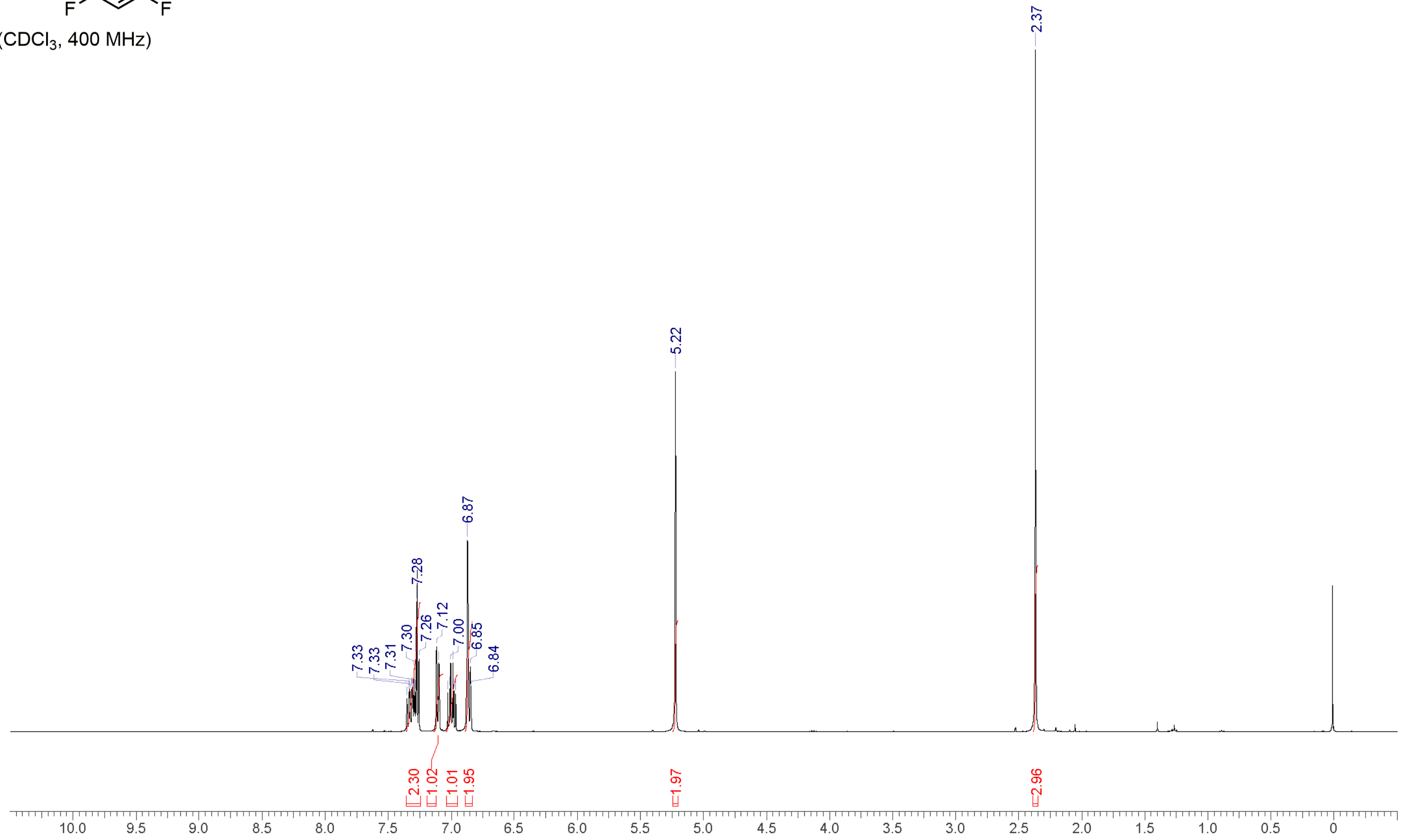

^{13}C NMR of compound **29**.

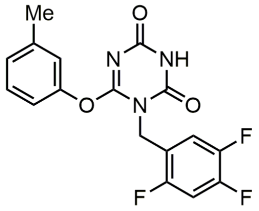

29 (CDCl$_3$, 100 MHz)

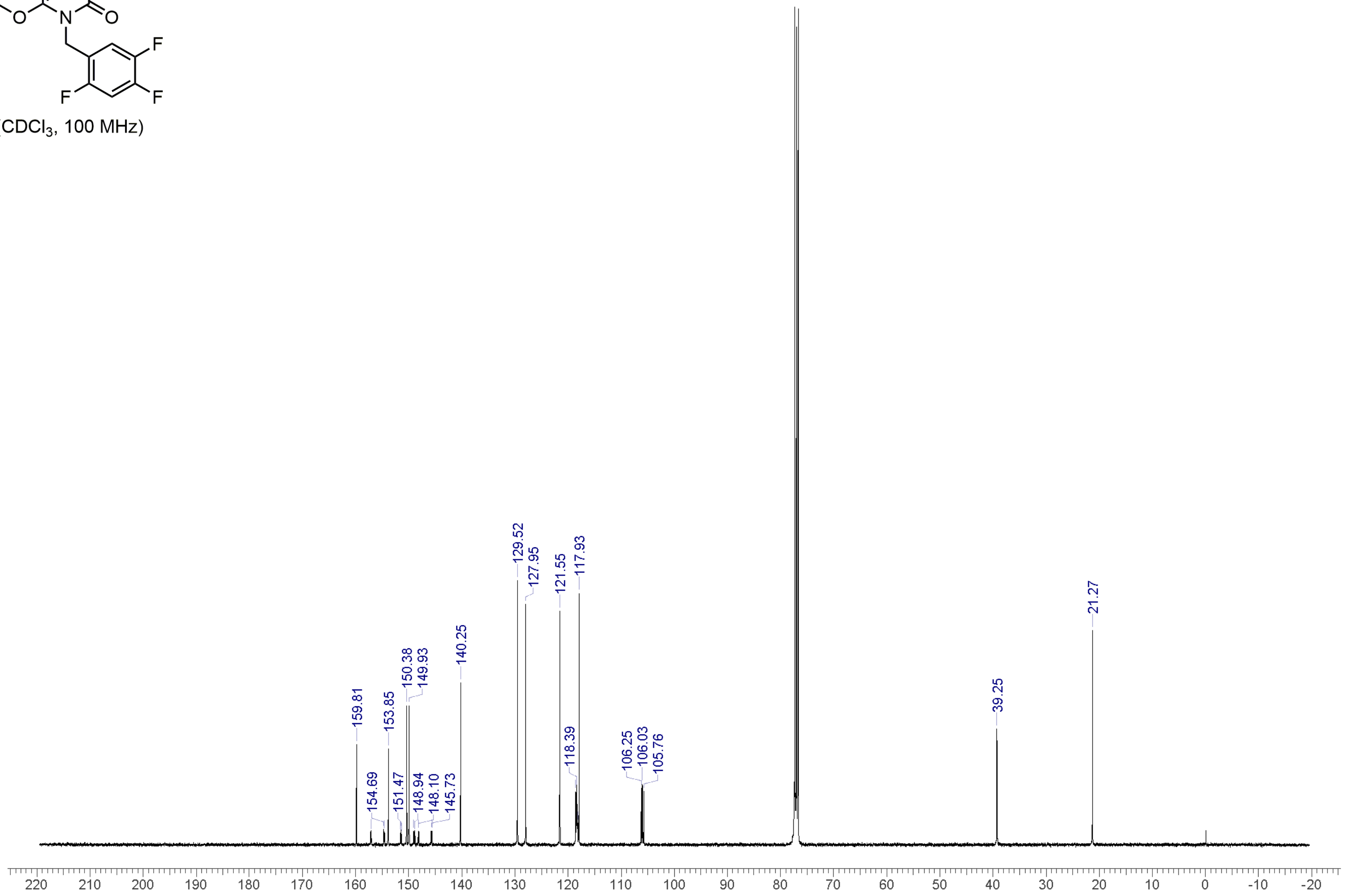

^{1}H NMR of compound **30**.

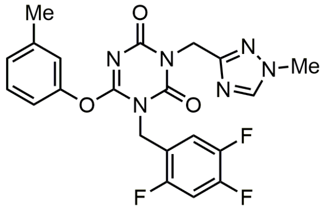

30 ($CDCl_3$, 400 MHz)

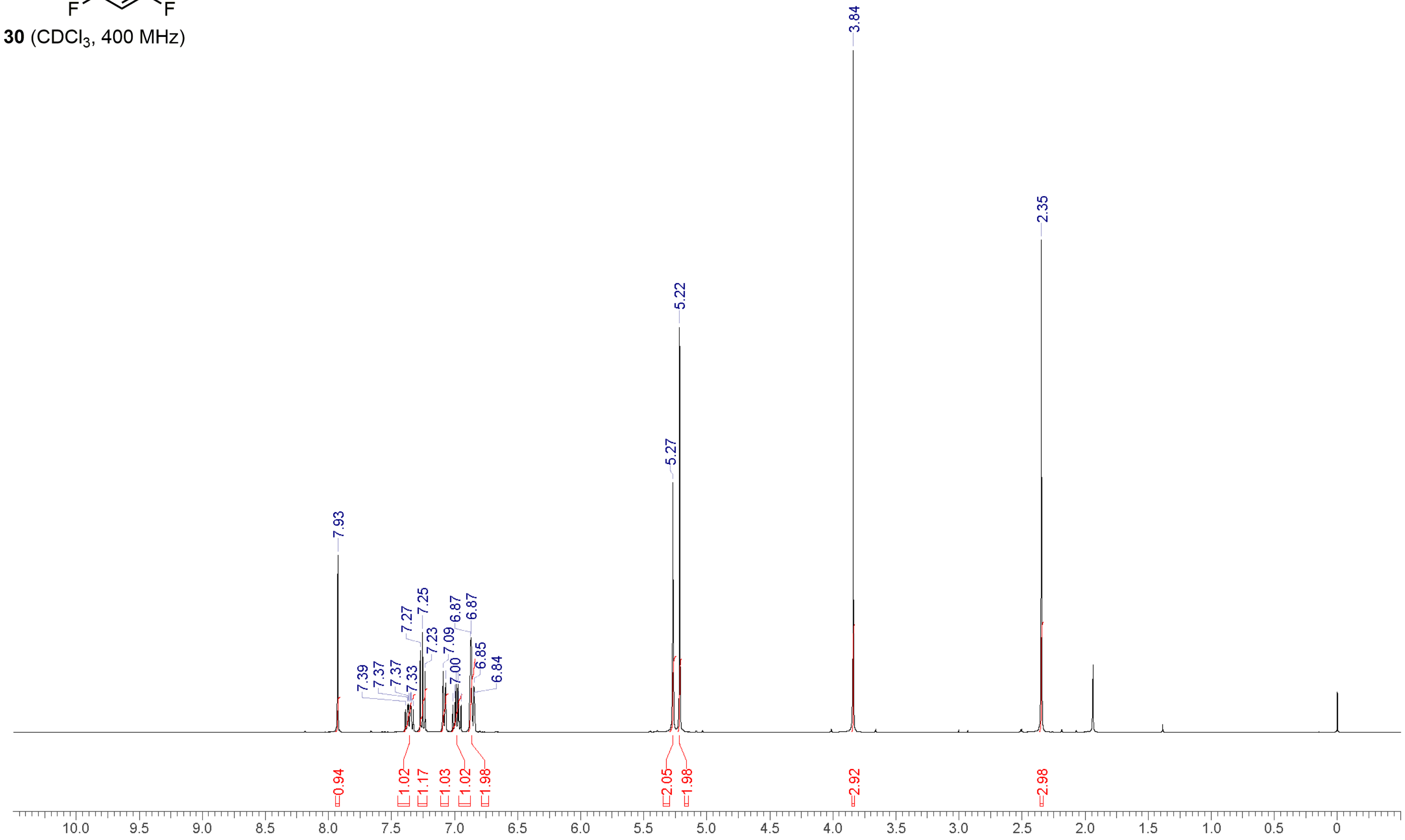

^{13}C NMR of compound **30**.

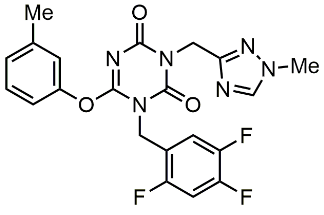

30 ($CDCl_3$, 100 MHz)

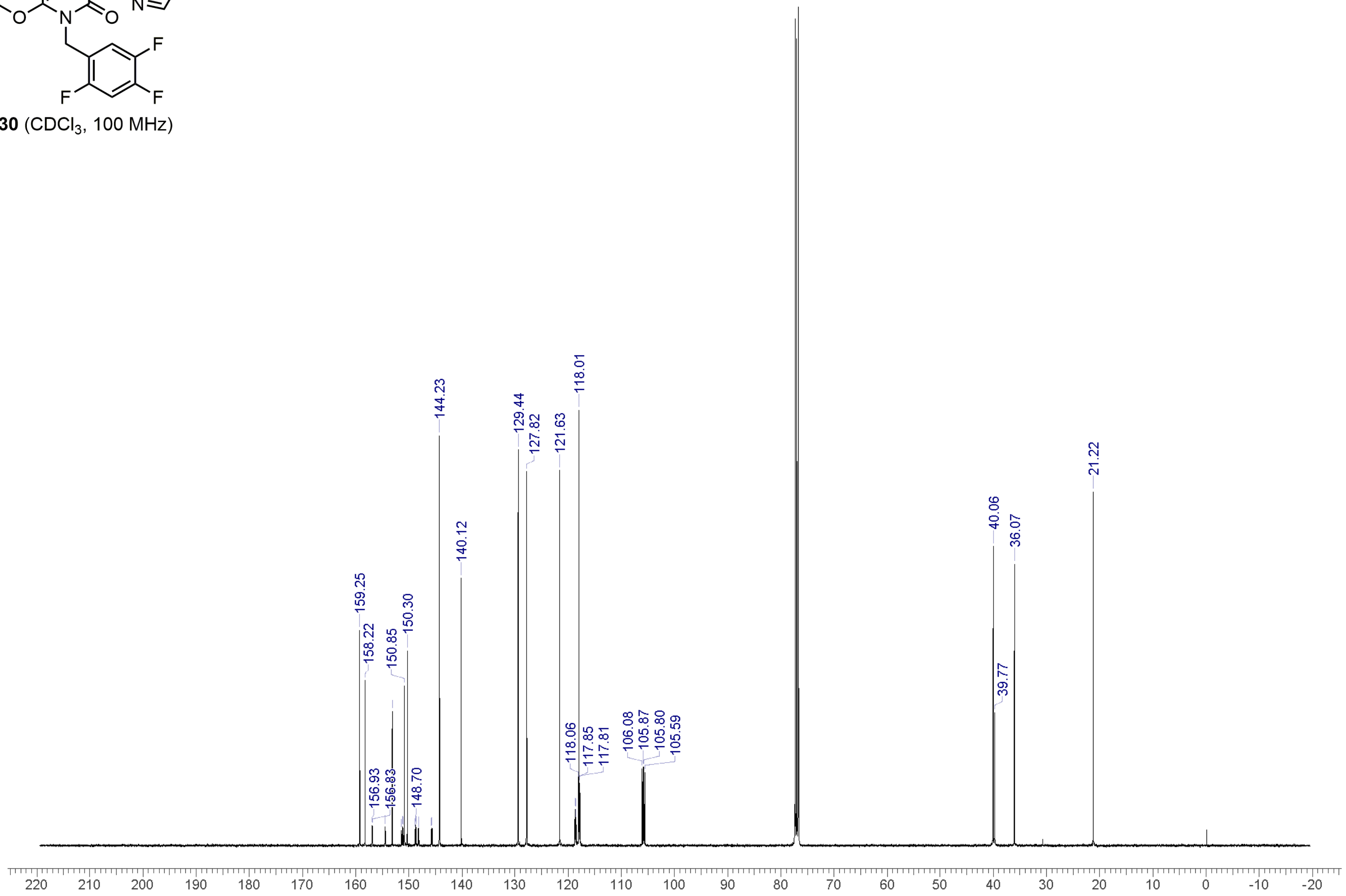

^{1}H NMR of compound **1**.

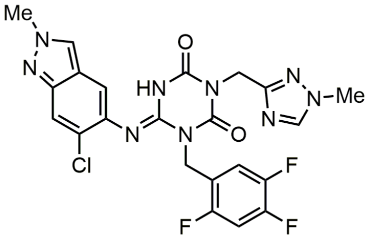

1 ($CDCl_3$, 400 MHz)

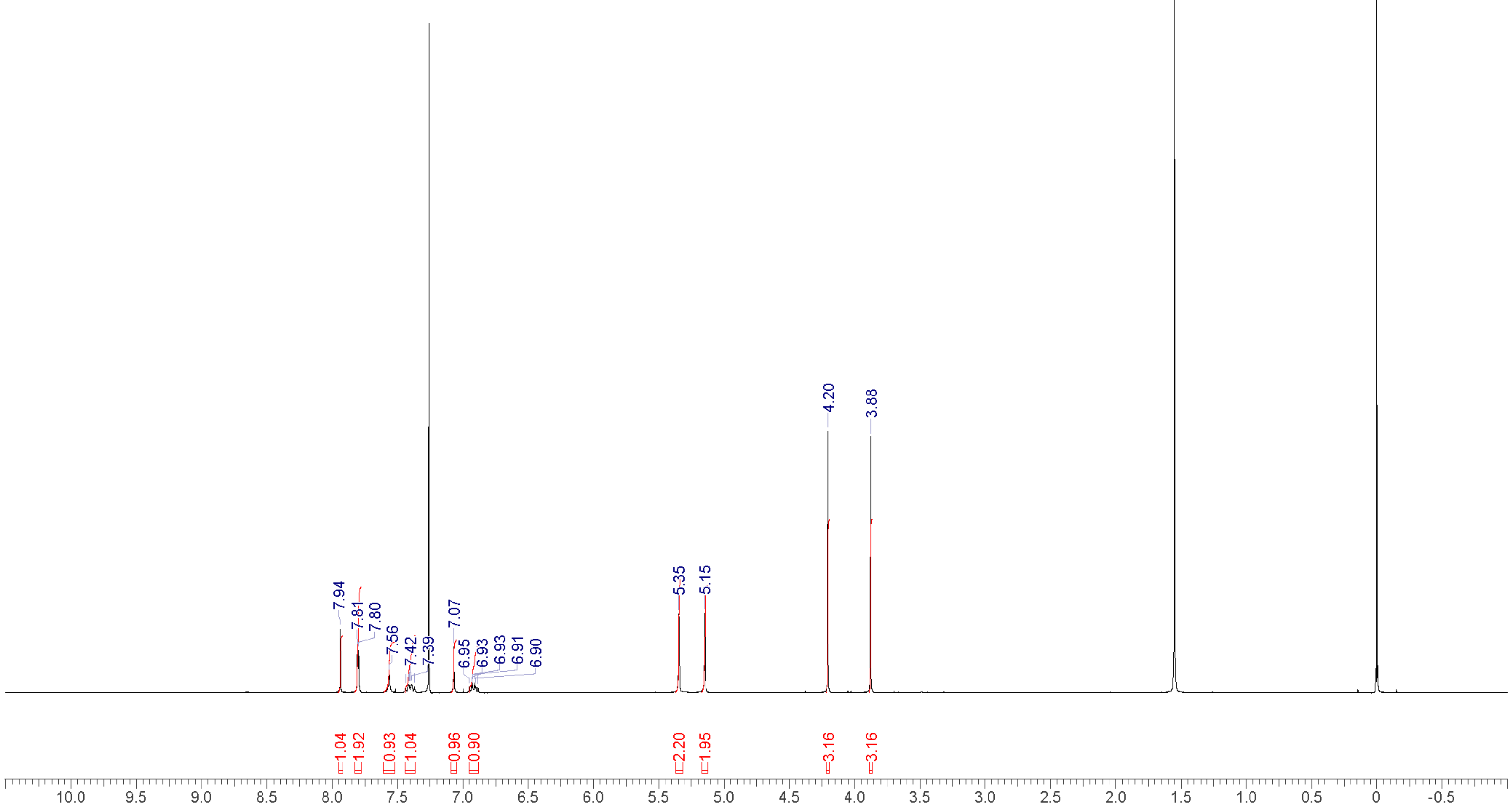

^{13}C NMR of compound **1**.

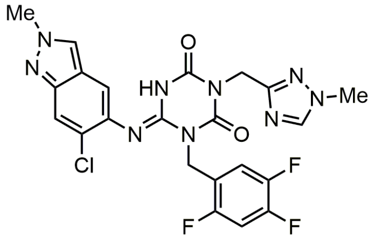

1 (THF-d_8, 100 MHz)

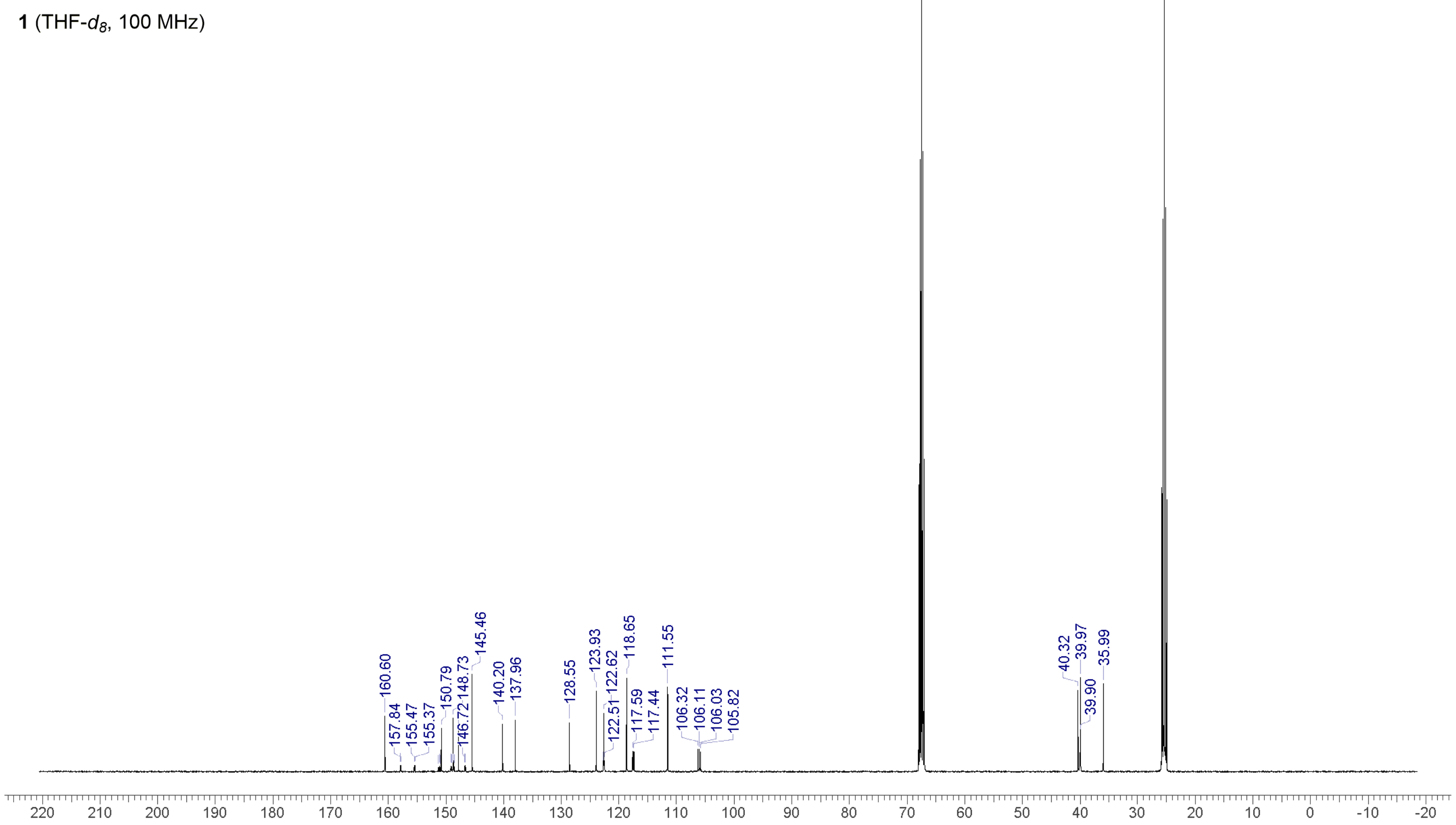

^{1}H NMR of compound **API**.

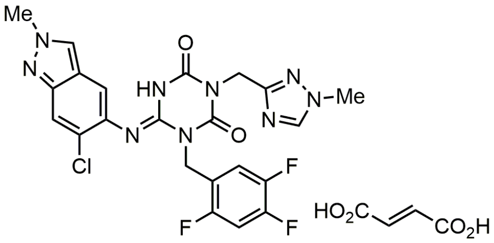

API (pyridine-d_5, 400 MHz)

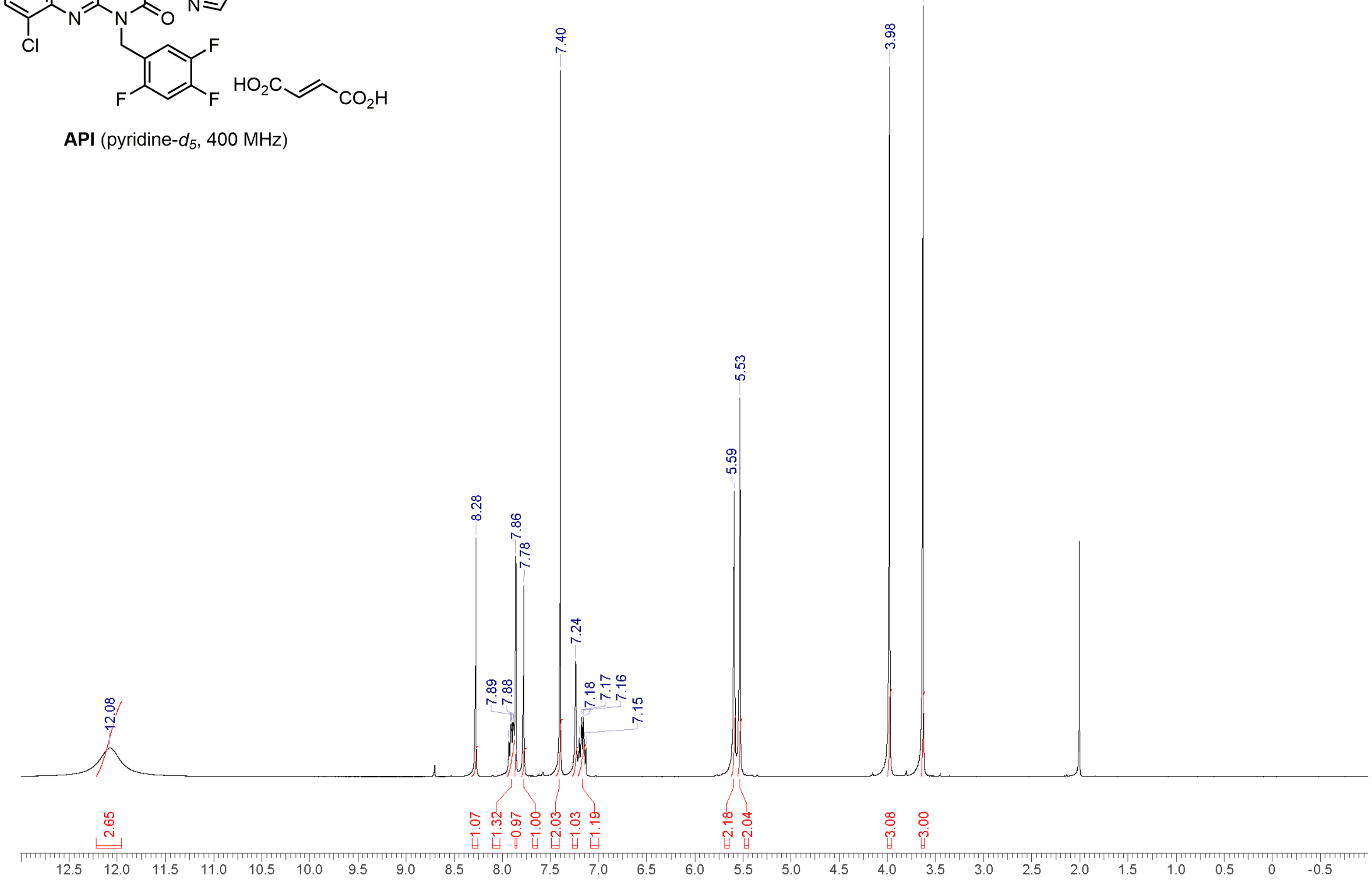

^{13}C NMR of compound **API**.

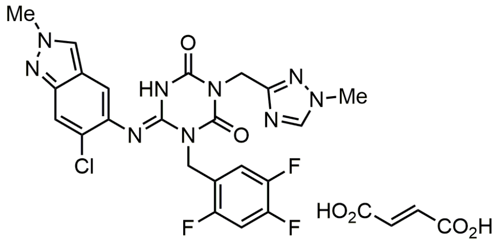

API (THF-d_8, 100 MHz)

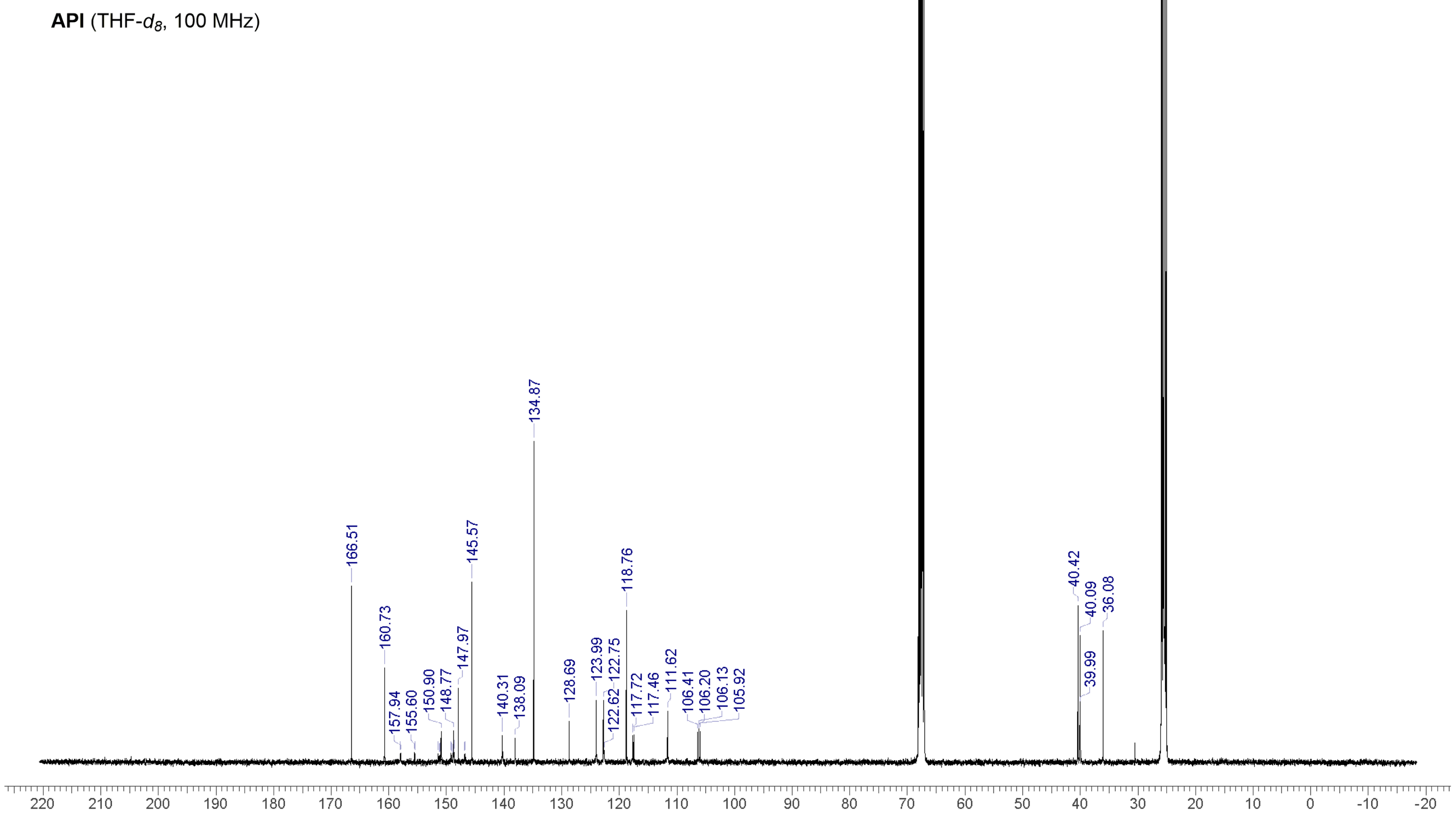

^{1}H NMR of compound **22**.

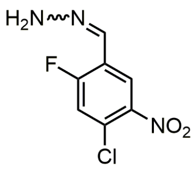

22 (DMSO-d_6, 400 MHz)

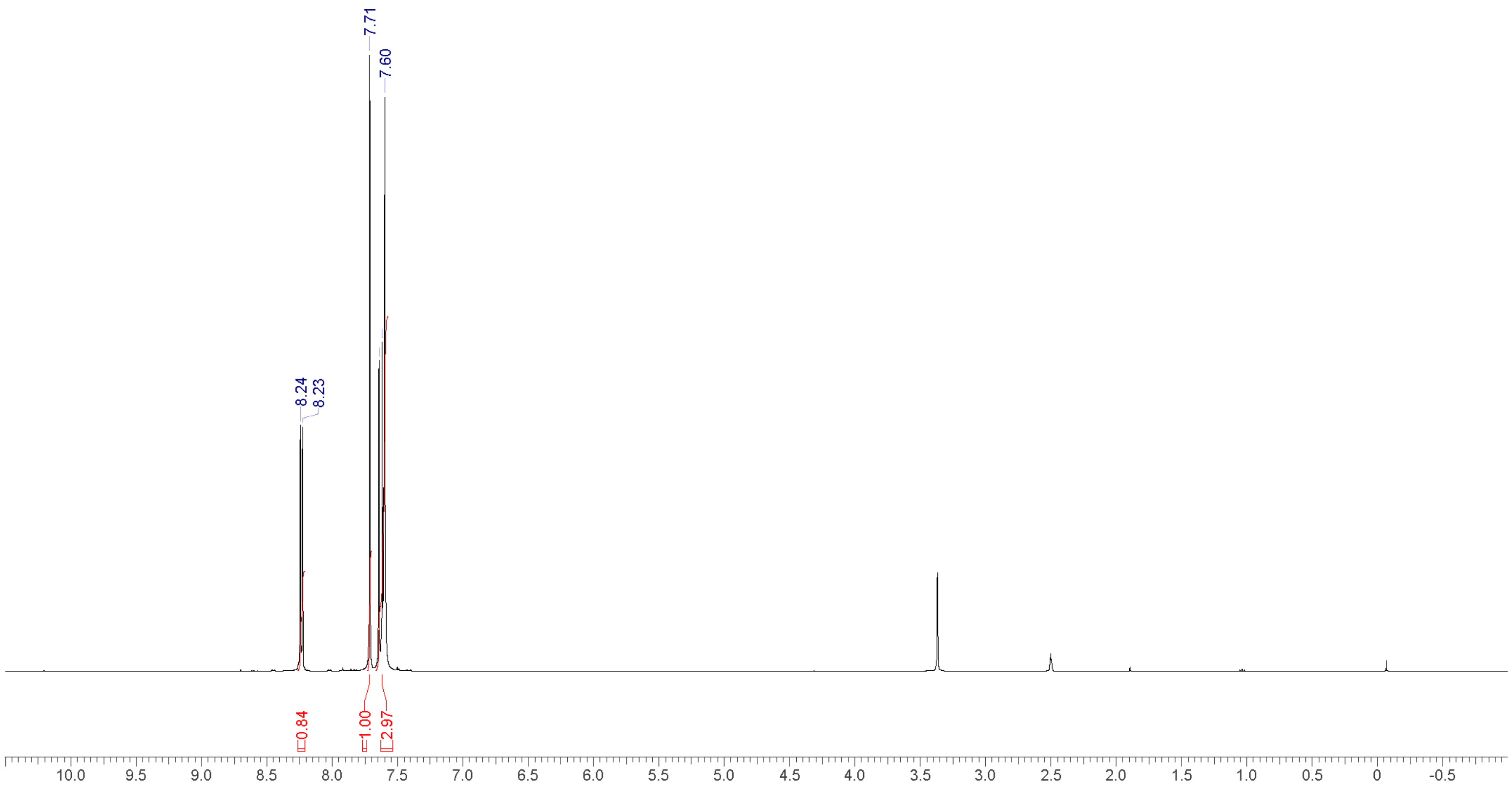

^{13}C NMR of compound **22**.

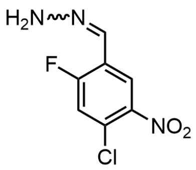

22 (DMSO-d_6, 100 MHz)

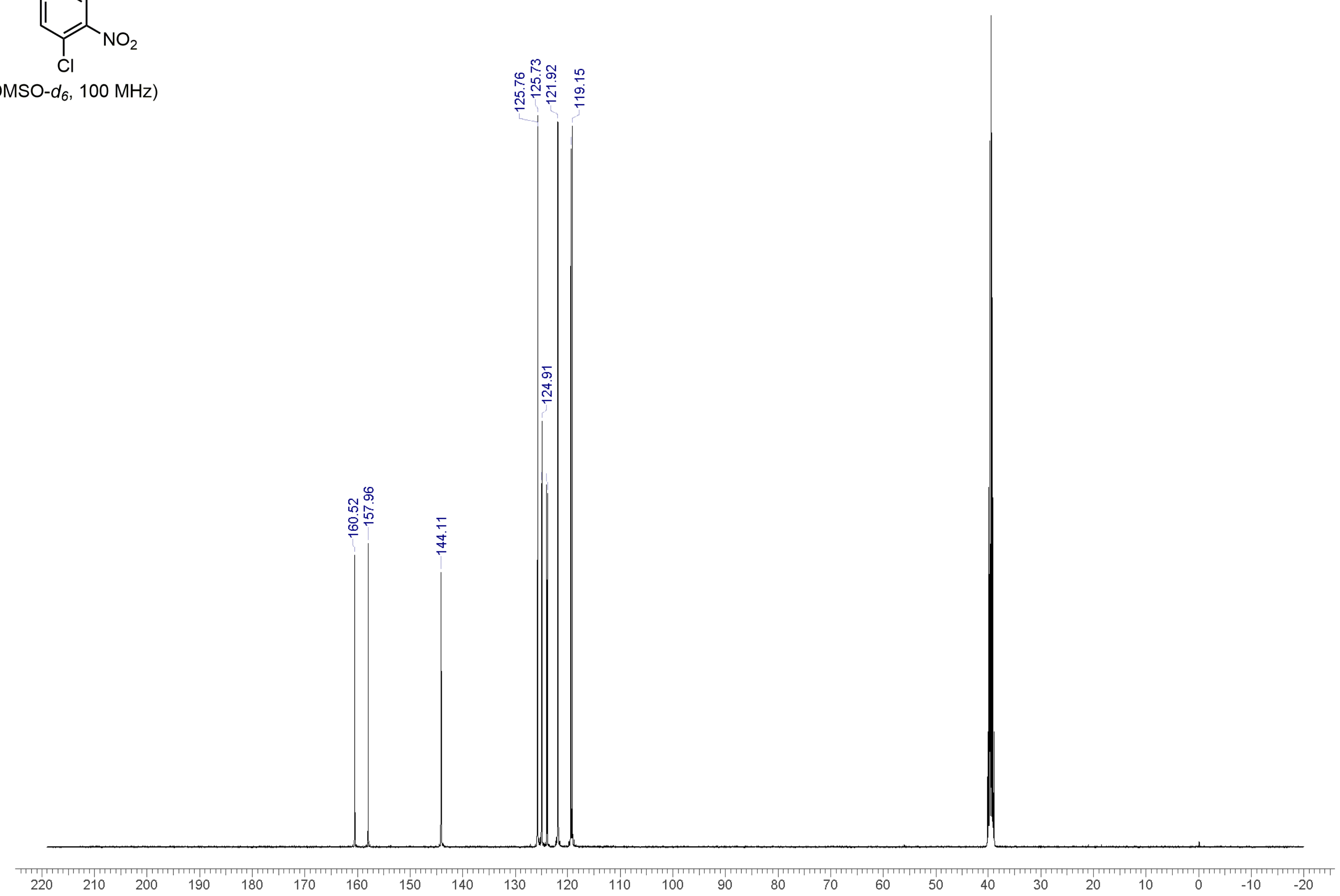

^{1}H NMR of compound **23**.

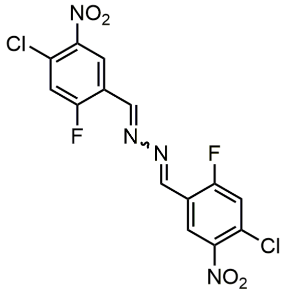

23 (DMSO-d_6, 400 MHz)

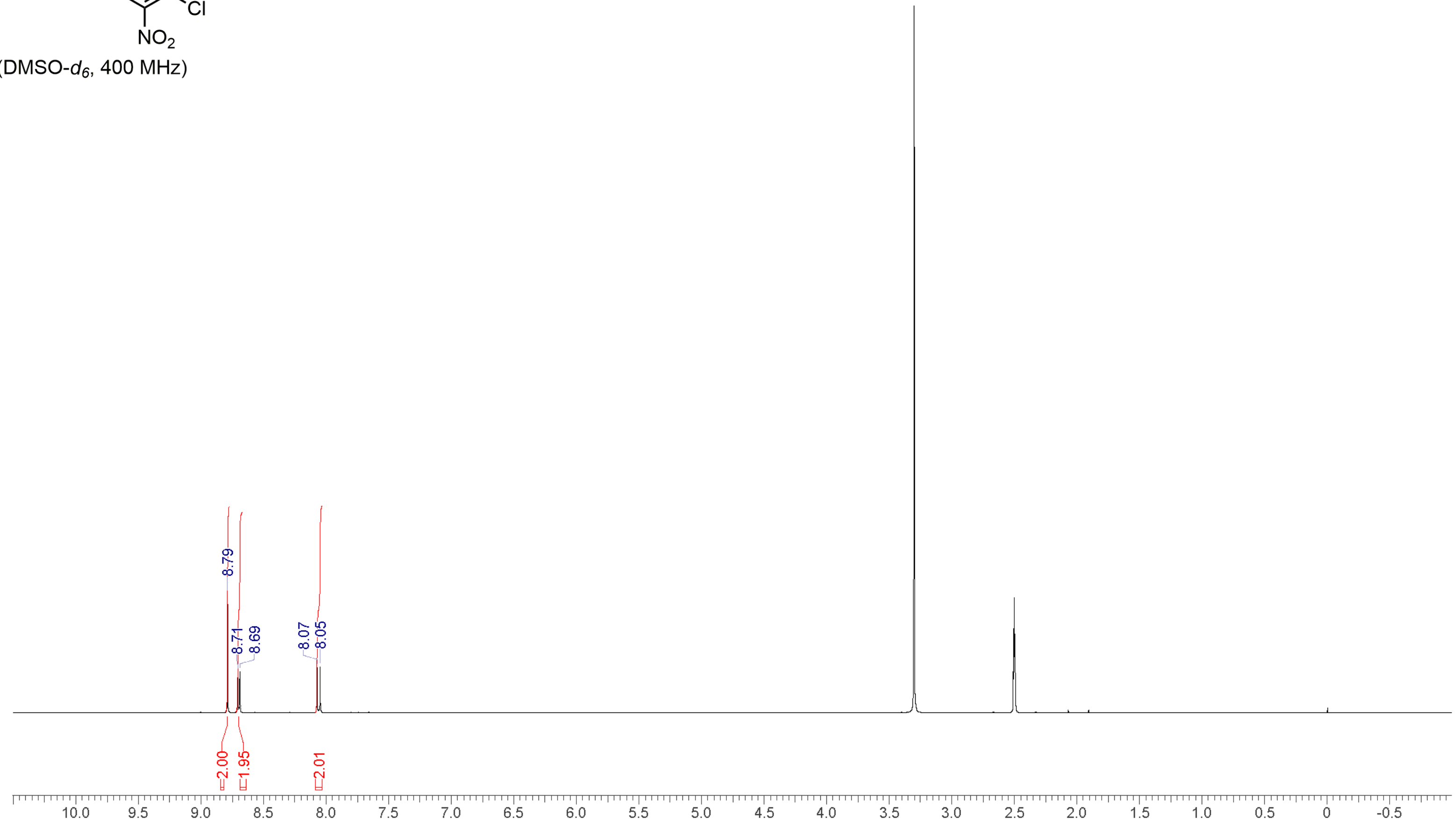

^{13}C NMR of compound **23**.

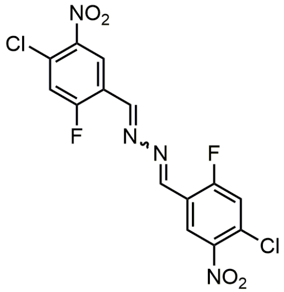

23 (DMSO-d_6, 100 MHz)

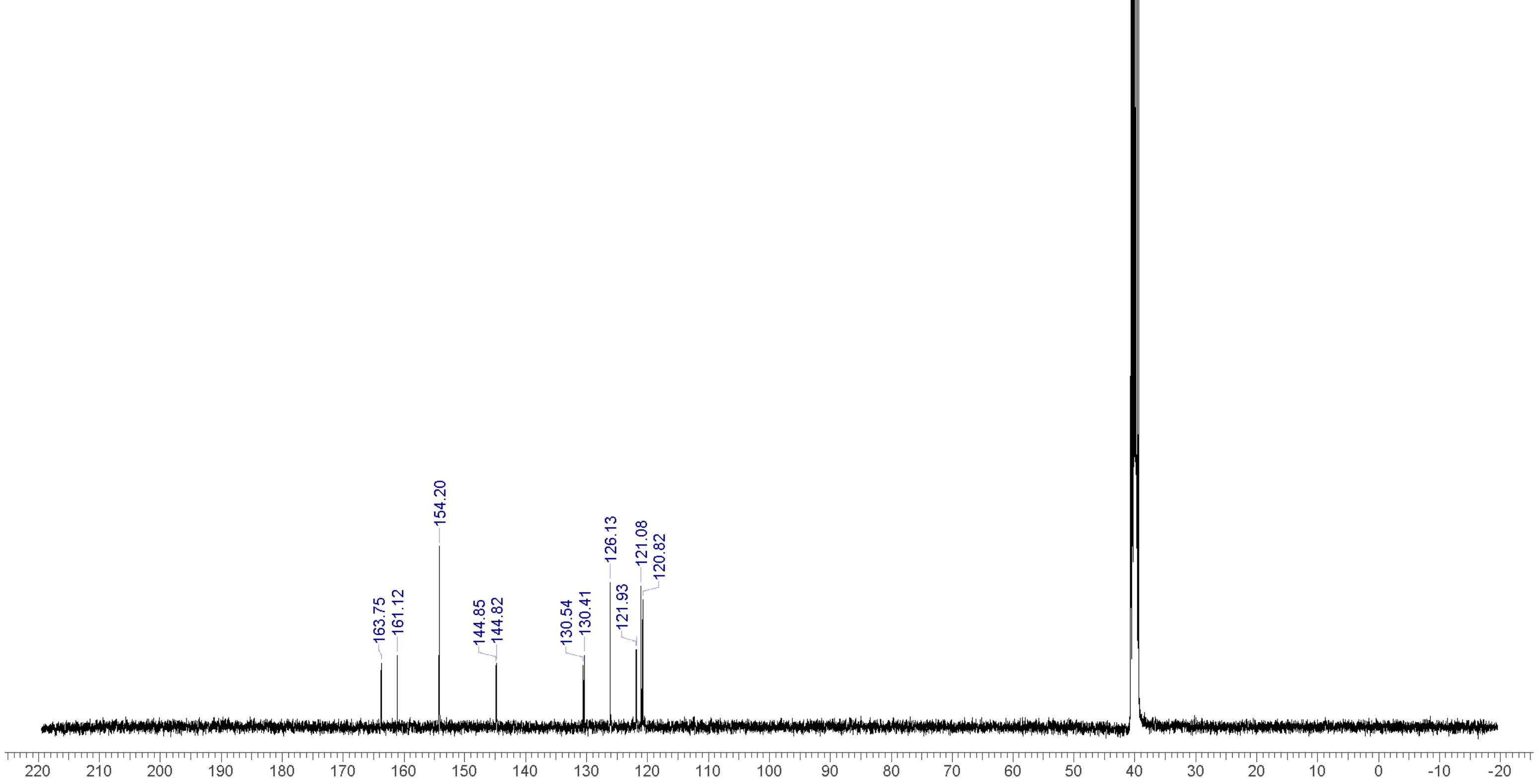

^{1}H NMR of compound **24**.

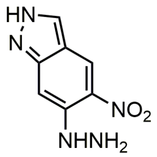

24 (DMSO-d_6, 400 MHz)

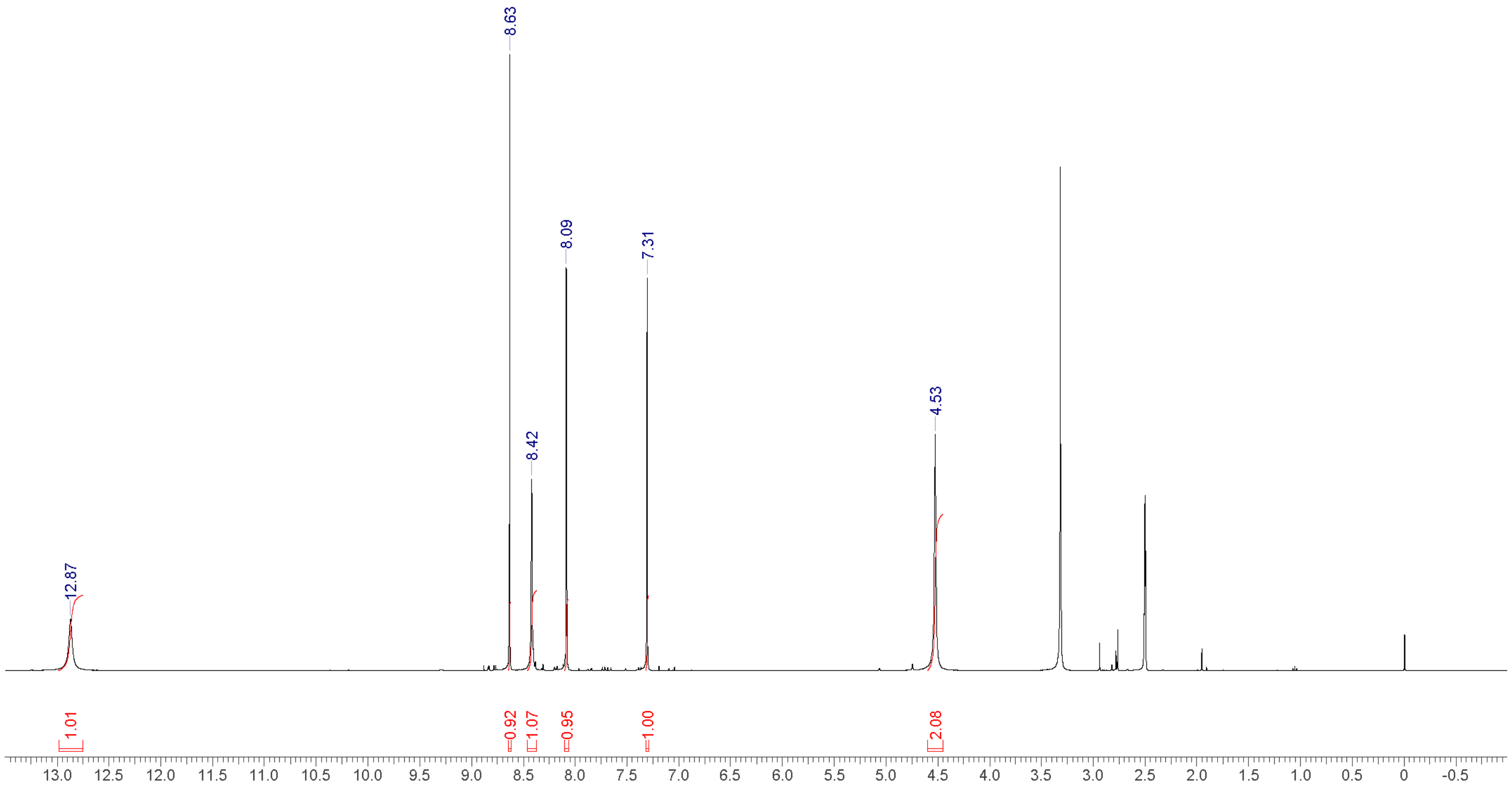

^{13}C NMR of compound **24**.

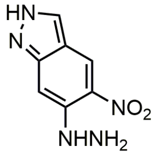

24 (DMSO-d_6, 100 MHz)

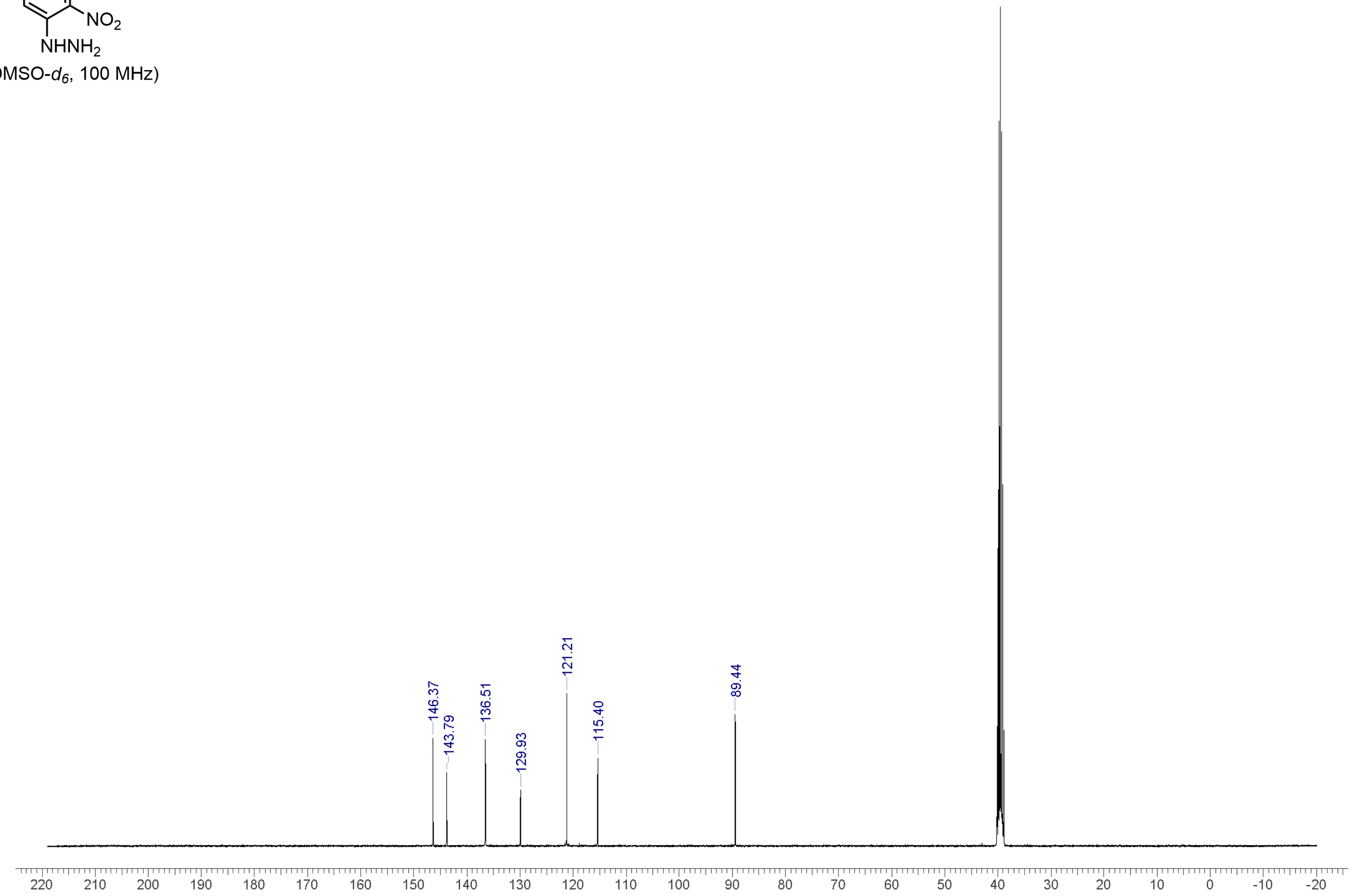

^{1}H NMR of compound **31**.

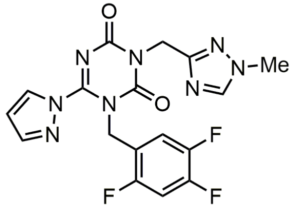

31 ($CDCl_3$, 400 MHz)

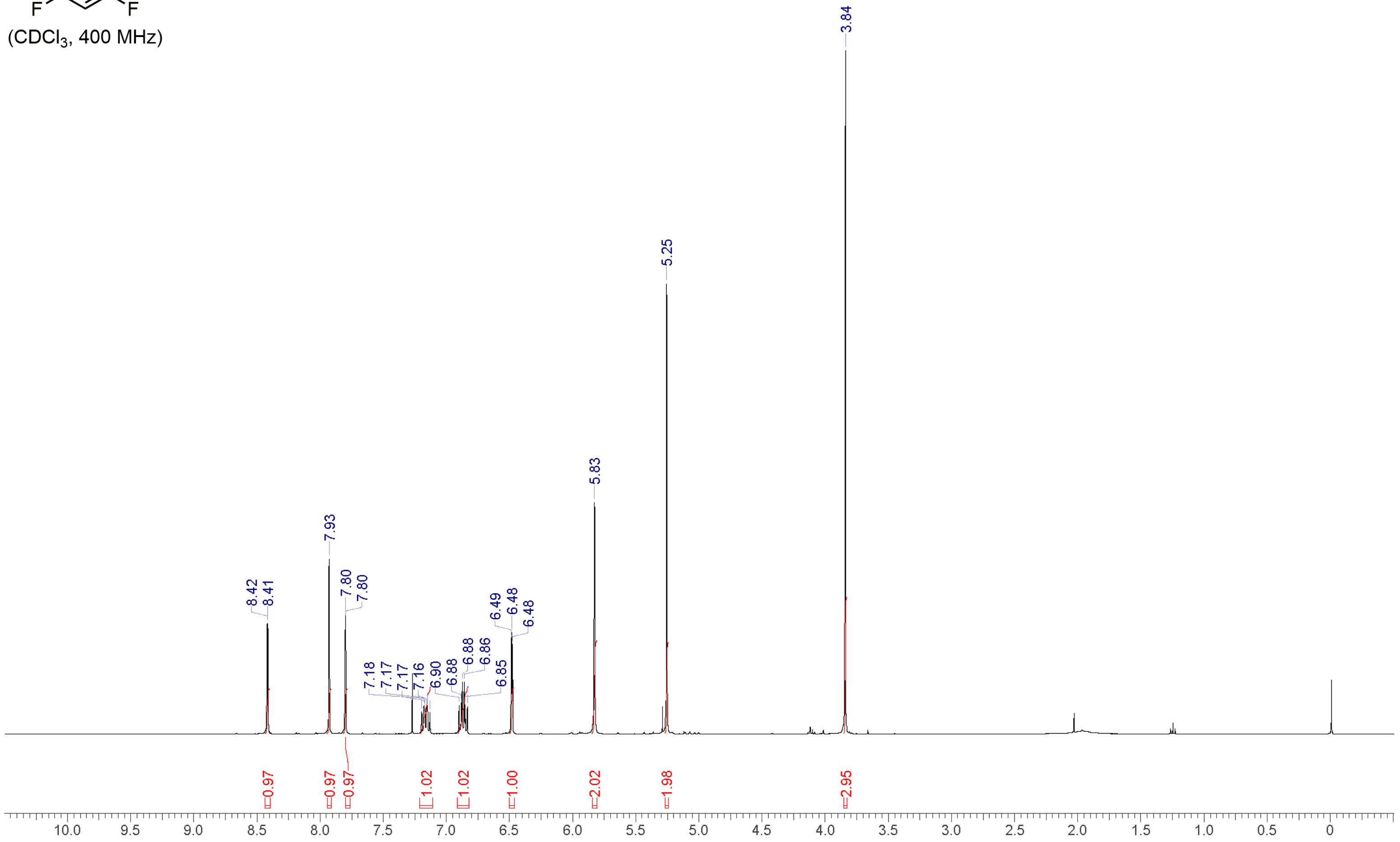

^{13}C NMR of compound **31**.

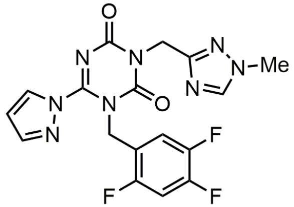

31 ($CDCl_3$, 100 MHz)

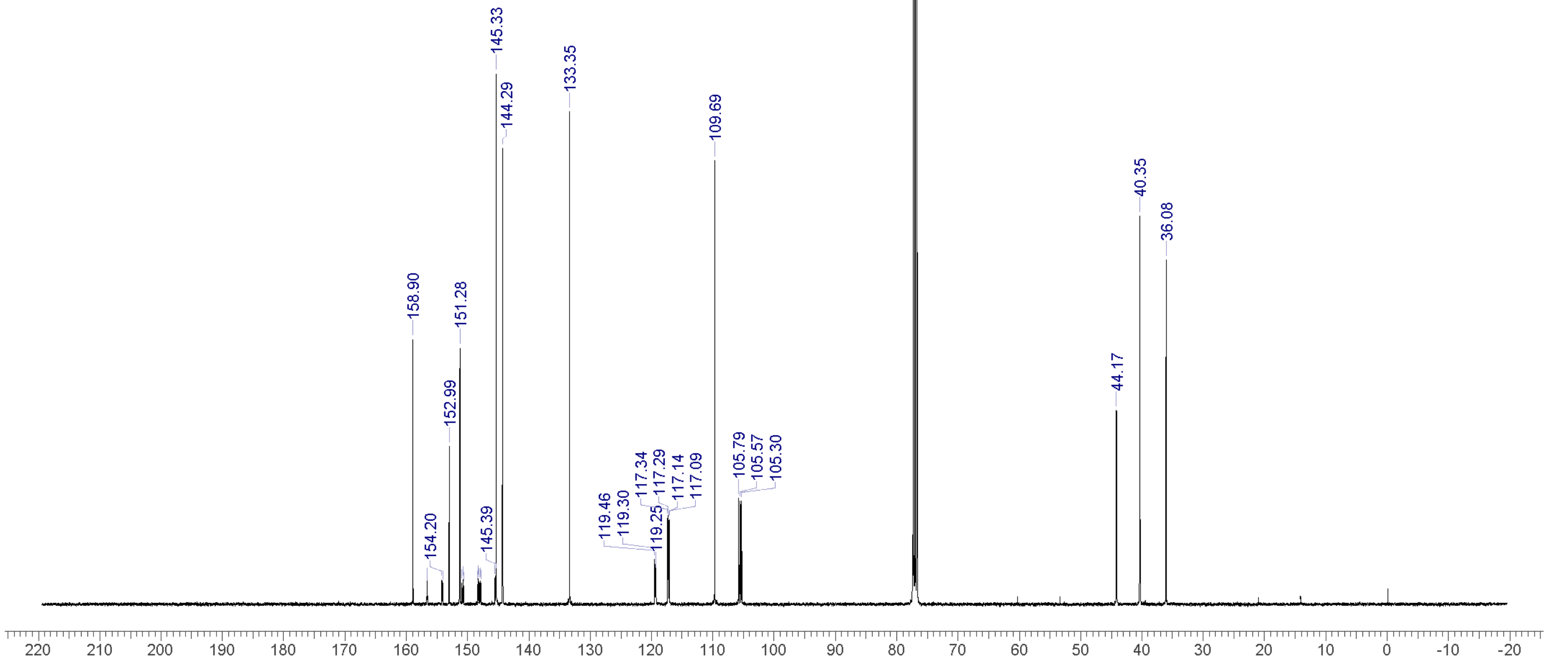

Reference

1) Tachibana, Y.; Uehara, S.; Unoh, Y.; Nakahara, K.; Taoda, Y.; Yamatsu, Y.; Ando, S.; Sasaki, M. Triazine derivative having virus propagation inhibitory effect, and pharmaceutical composition containing same. WO 2022138988 A1, Feb. 17th, 2022.
