# Supplementary material for: Development of a Manufacturing Process toward the Convergent Synthesis of the COVID-19 Antiviral Ensitrelvir
Source: ACS Cent Sci. 2023 Apr 7;9(4):836–43. doi: 10.1021/acscentsci.2c01203 (PMC10108738; doi:10.1021/acscentsci.2c01203)
Supplement: Supplementary file 2 — oc2c01203_si_002.pdf [file oc2c01203_si_002.pdf]

oc-2022-01203u.R1

Name: Peer Review Information for "Development of a manufacturing process toward the convergent synthesis of the COVID-19 antiviral Ensitrelvir"

First Round of Reviewer Comments

Reviewer: 1

Comments to the Author

Manuscript Summary:

An efficient large-scale synthesis of the SARS-CoV-2 antiviral drug Ensitrelvir is described. This is the first non-peptidic and non-covalent 3CL inhibitor and complements the drugs developed by Merck and Pfizer. The highly convergent approach utilizes multiple fragment couplings to rapidly (6 steps, LLS) produce the target drug, and has been demonstrated to work in high overall yields (35% yield) on hundred-kilogram scale. The approach outlined should allow for efficient generation of this important antiviral drug, and therefore efficacious treatment of symptoms of COVID-19.

Manuscript General Comments:

The manuscript is well written and has few grammatical or word-choice errors. The background and hypotheses are clearly outlined, and the development of the synthesis is clearly communicated.

The process described is a clear improvement on the previous state of the art (medicinal chemistry route) and should contribute greatly to the production of this important drug candidate for the treatment of COVID-19 symptoms.

In various points in the manuscript the authors use the phrase “medicinal synthetic stage” or “medicinal chemistry synthetic stage”. This is not a phrase the reviewer is familiar with but is under the impression the author means to refer to one of the early stages of drug development, such as research and development. See the line-by-line comments for each mention of this phrase. Overall, this should be a good contribution to the literature on syntheses of COVID-19 medicines.

Minor edits to the grammar and word choice have been suggested for clarity below. Please see the line-by-line for details.

Manuscript Line-by-Line Comments:

As mentioned above, “medicinal synthetic stage” or “medicinal chemistry synthetic stage” is mentioned in the following places: page 3, line 10; page 4, line 40; page 5, line 20–22; page 10, line 3; page 10, line 23; page 14, line 12. Perhaps, a better statement would be “at the research and development stage” to differentiate it from the “process chemistry stage”

Page 3, Line 30: the word “unfettered” is grammatically incorrect within the sentence and should be removed altogether for clarity.

Page 3, Line 33: “COVID-19 has been recognized as one of the illnesses for which a cure is the most strongly desired in human history” is awkwardly written. This should be removed or rephrased to something like “COVID-19 has been recognized as a palpable threat that demands efficacious treatment”.

Page 3, Line 51–53: “collaboration research of” should be changed to “research collaboration between”.

Page 3, Line 56: “properties in terms of” should be removed for conciseness.

Page 6, Line 42: “like LiAlH4” should be changed to “of LiAlH4”. Alternatively, the preceding “the hazardous” could be changed to “hazardous”, omitting the “the” while “like LiAlH4” is kept as is.

Page 6, Line 47: “known as Rochelle salt” should have a possessive “’s” to read as “known as Rochelle’s salt” as well as a comma before and after, as this is a dependent clause, to read “, known as Rochelle’s salt,”.

Page 7, Line 20–22: “methodologies of indazole derivatives” should be changed to “methodologies for the synthesis of indazole derivatives”.

Page 7, Line 27: “indazole” should be followed with a bolded compound number.

Page 7, Line 36: the bolded compound number should not be in parentheses, as this is part of the proper noun of the sentence. It should read as “to afford the corresponding compound 15,”. This is the case THROUGHOUT the manuscript and should be changed in all other instances as well.

Page 7, Line 45: “important” is redundant and should be removed for conciseness.

Page 8, Line 29: “Meerwein Reagent” should be written with a possessive “’s” to read “Meerwein’s Reagent”.

Page 8, Line 39: “As a matter of fact” should be removed. Alternatively, one could say something like: “Consistent with these reports,”.

Page 8, Line 52: “was” should be changed to “is”.

Page 10, Line 36: “27 crystals” should be changed to “crystals of 27”.

Page 11, Line 3: there should be a “the” prior to “tert-butyl moiety”.

Page 11, Line 6: “enabling avoidance” should be changed to “obviating” for conciseness.

Page 12, Line 3–6: it is unclear to the reviewer the meaning of “The present process without evaporation of TFA could facilitate equipment selection.” And could be removed for clarity.

Page 12, Line 31: there should be an “a” prior to “70% yield”. Alternatively, one could replace “to obtain” with “in”.

Page 12, Line 45: “unapplicable” should be changed to “hazardous” or some other descriptor, as unapplicable doesn’t make sense here. Does the author mean that the reagents were used with difficulty? If so, this should be changed to convey this.

Page 13, Line 6: “This described process to 1” chould be changed to “the process to 1 just described” or “the described process to 1” for smoothness.

Page 17, Line 10: DIOs should not be included in references. Please change your reference formatting to match that of refences 2–8.

Page 18, Line 5: DIOs should not be included in references. Please change your reference formatting to match that of refences 2–8.

At one point, the word “subsequentially” is used. This should be changed to “subsequently”

Supplementary Information General Comments:

The supplementary information includes high quality spectra, summaries of the outlined work, and methods workflows for optimization. No changes or edits have been suggested.

Reviewer: 2

Comments to the Author

This is a review of the manuscript submitted to ACS Central Science titled “Development of a manufacturing process toward the convergent synthesis of the COVID-19 antiviral Ensitrelvir” by Takahiro Kawajiri, Akihito Kijima, Atsuhiro Iimuro, Eisaku Ohashi, Katsuya Yamakawa, Kazushi Agura, Kengo Masuda, Kensuke Kouki, Koji Kasamatsu, Shuichi Yanagisawa, Sho Nakashima, Setsuya Shibahara, Takashi Toyota, Takafumi Higuchi, Takahiro Suto, Tadashi Oohara, Toshikatsu Maki, Naoto Sahara, Nobuaki Fukui, Hisayuki Wakamori, Hidaka Ikemoto, Hiroaki Murakami, Hiroyasu Ando, Masahiro Hosoya, Mizuki Sato, Yusuke Suzuki, Yuta Nakagawa, Yuto Unoh, Yoichi Hirano, Yoshitomo Nagasawa, Satoshi Goda, Takafumi Ohara, and Takayuki Tsuritani.

The authors describe the development of a practical, concise, and scalable route to the oral 3C-like protease inhibitor ensitrelvir (1) for the treatment of COVID-19. The process features the synthesis and convergent coupling of key indazole, triazole and triazinone fragments, with convenient purification via crystallization that eliminated the need for column chromatography and provided the API in >35% overall yield. The manuscript is well written, and I compliment the authors on highlighting the key drivers for their process design throughout – e.g. developing direct-drop isolations where no extractions are required as a superior alternative when dealing with water soluble intermediates (like triazole 13). The detailed impurity profile mapping of the synthetic route leading to indazole 10 is also very impressive. Finally, introduction of the m-cresolyl unit to enhance the stability of process intermediates and improve process control was an impressive design element that enabled a scalable preparation of 1.

Overall, this is an excellent contribution on the development of an efficient and robust process from the research team at Shionogi & Co. However, given the broad and diverse readership of ACS Central Science and the more specialized nature of this submission, my recommendation is to publish in a specialized journal, such as OPRD, after minor revisions, particularly regarding experimental aspects of the work.

Suggested revisions:

My overall impression of the experimental section in the Supporting Information is that it is too light on detail. A high-level summary of the scale up recipe is not enough and details on the process (unit operations) including temperatures, heat-cool times, feed rates, drying conditions etc. are important to ensure reproducibility. For example, the description of the ensitrelvir – fumaric acid crystallization process indicates that a solution of the API free base in acetone/water was treated with activated carbon but does not reveal how much carbon was used, how long the slurry was held, and doesn't indicate that the carbon was even removed via filtration (I assume it was). In addition, details on other unit operations are missing e.g. how was the mixture concentrated to 36.0 volumes – distillation over how long and at what temperature / pressure? Was this a seeded crystallization? How much acetone was used to wash the product? What pressure was the product dried at? For how long? Was an agitated filter dryer used? Other? What was the melting point of the product thus obtained? Any solid form characterization to be shared? XRPD?

Similar comments hold for all the other procedures provided – details are missing throughout (e.g. for compound 28, details of the direct drop isolation procedure including water feed rate, hold times, cooling rates, seed loading, cake washing volumes and number of washes etc are all missing)

A lot of optimization work was performed in developing a scalable (100s of kg) reduction process to afford compound 10. The final process employs a catalytic hydrogenation using Pt/C. Nitro reductions are known to proceed via the intermediacy of nitroso and hydroxyl amine species and disproportionation reactions commonly lead to dimeric byproducts including azoxy, azo, and hydrazo species which can be considered potentially mutagenic impurities (PMIs).

1. How were these PMIs controlled in the process?

2. Additionally, catalytic hydrogenations of nitro compounds can also be highly exothermic – was this observed in this case and how was it controlled? Was there any accumulation of energetic species like hydroxylamine etc?

3. Were additional steps needed to eliminate residual metal in the product (metal scavengers etc)?

During the synthesis of compound 30, it was noted that Cs2CO3 was superior to K2CO3 in improving the 'reproducibility' of the reaction. Do the authors believe this due to differences in particle size or water levels for the K2CO3 lots examined?

The final API is described as a ensitrelvir-fumaric acid co-crystal. What evidence do the authors have that this material is obtained as a co-crystal versus a traditional fumarate salt? Is their single crystal X-Ray or NMR data or other that are consistent with a co-crystal versus salt? Physical characterization of this material should be included (mp etc)

Reviewer: 3

Comments to the Author

The manuscript goes into great detail on the synthesis of a critical COVID-19 antiviral compound, Ensitrelvir. It is extremely well written, both the manuscript and supporting information, and showcases a beautiful story of process chemistry. While one could suggest it may be more suited for a journal such as OPRD, my opinion is the well-developed process, coupled with the importance of the target (an oral antiviral that can be taken without high risk of DDIs) make it suitable to the readership of ACS Central Science.

As a general comment, I would suggest that the authors revise the manuscript to highlight the novelty and challenges inherent to each of the individual transformations rather than the focusing on the ultimate conditions which were utilized. While these generally appear to be straightforward, on the scale the authors operate on, numerous process challenges are bound to have arisen. While the authors provide sufficient understanding around the advantage of the meta-cresolyl moiety, additional detail should be provided to help the reader understand how the authors arrived at that strategy. What experiments were conducted? Was the use of modelling employed?

Additionally, additional detail around the final API crystallization with fumaric acid should be provided. This is a key point which is not fully explained, both from a crystallization process and biological significance perspective.

One very minor comment - on S19, the word "acetone" is misspelled in a scheme.

Overall, with minor edits to highlight the novelty of the process, I think this is a very appealing story for the readers of ACS Central Science.

Author's Response to Peer Review Comments:

Dear Dr. Editor,

Manuscript ID: oc-2022-01203u

Title: "Development of a manufacturing process toward the convergent synthesis of the COVID-19 antiviral Ensitrelvir"

I appreciate the time spent by you and the reviewers. I believe the revised manuscript is improved. Below, we have addressed your and the reviewers' comments.

sincerely,

Takahiro Kawajiri

Formatting Needs:

SI STATEMENT: Because your manuscript is accompanied by Supporting Information for publication, a brief description of the supplementary material is required in the manuscript. The appropriate format is: Supporting Information (header), followed by a brief statement in nonsentence format listing the contents of the material supplied as Supporting Information.

[Reply] It was appropriately rectified.

Response to Reviewer 1

1. In various points in the manuscript the authors use the phrase "medicinal synthetic stage" or "medicinal chemistry synthetic stage". This is not a phrase the reviewer is familiar with but is under the impression the author means to refer to one of the early stages of drug development, such as research and development. See the line-by-line comments for each mention of this phrase.
   As mentioned above, "medicinal synthetic stage" or "medicinal chemistry synthetic stage" is mentioned in the following places: page 3, line 10; page 4, line 40; page 5, line 20–22; page 10, line 3; page 10, line 23; page 14, line 12. Perhaps, a better statement would be "at the research and development stage" to differentiate it from the "process chemistry stage"

   [Reply] Thank you for your constructive feedback. For better clarity, we rectified it to "at the early research and development stage".

2. Page 3, Line 30: the word "unfettered" is grammatically incorrect within the sentence and should be removed altogether for clarity.

   [Reply] It was removed according to the comment.

3. Page 3, Line 33: "COVID-19 has been recognized as one of the illnesses for which a cure is the most strongly desired in human history" is awkwardly written. This should be removed or rephrased to something like "COVID-19 has been recognized as a palpable threat that demands efficacious treatment".

[Reply] It was rephrased according to the comment.

4. Page 3, Line 51–53: “collaboration research of” should be changed to “research collaboration between”.

   [Reply] It was changed according to the comment.

5. Page 3, Line 56: “properties in terms of” should be removed for conciseness.

   [Reply] It was removed according to the comment.

6. Page 6, Line 42: “like LiAlH4” should be changed to “of LiAlH4”. Alternatively, the preceding “the hazardous” could be changed to “hazardous”, omitting the “the” while “like LiAlH4” is kept as is.

   [Reply] “the” prior to “hazardous” was removed according to the comment.

7. Page 6, Line 47: “known as Rochelle salt” should have a possessive “’s” to read as “known as Rochelle’s salt” as well as a comma before and after, as this is a dependent clause, to read “, known as Rochelle’s salt,”.

   [Reply] According to the literature (e.g. *Org. Process Res. Dev.* **2017**, *21*, 1145–1155; *Org. Lett.* **2014**, *16*, 5890–5893.), “Rochelle salt” seems to be correct. A comma was added according to the comment.

8. Page 7, Line 20–22: “methodologies of indazole derivatives” should be changed to “methodologies for the synthesis of indazole derivatives”.

   [Reply] It was changed according to the comment.

9. Page 7, Line 27: “indazole” should be followed with a bolded compound number.

   [Reply] A bolded compound number was added according to the comment.

10. Page 7, Line 36: the bolded compound number should not be in parentheses, as this is part of the proper noun of the sentence. It should read as “to afford the corresponding compound 15,”. This is the case THROUGHOUT the manuscript and should be changed in all other instances as well.

    [Reply] All parentheses associated with the bolded compound numbers were removed in accordance with the comment.

11. Page 7, Line 45: “important” is redundant and should be removed for conciseness.

    [Reply] It was removed according to the comment.

12. Page 8, Line 29: “Meerwein Reagent” should be written with a possessive “’s” to read “Meerwein’s Reagent”.

    [Reply] It was rectified according to the comment.

13. Page 8, Line 39: “As a matter of fact” should be removed. Alternatively, one could say something like: “Consistent with these reports,”.

[Reply] It was rephrased according to the comment.

14. Page 8, Line 52: “was” should be changed to “is”.

    [Reply] It was corrected according to the comment.

15. Page 10, Line 36: “27 crystals” should be changed to “crystals of 27”.

    [Reply] It was corrected according to the comment.

16. Page 11, Line 3: there should be a “the” prior to “tert-butyl moiety”.

    [Reply] It was added according to the comment.

17. Page 11, Line 6: “enabling avoidance” should be changed to “obviating” for conciseness.

    [Reply] It was rectified according to the comment.

18. Page 12, Line 3–6: it is unclear to the reviewer the meaning of “The present process without evaporation of TFA could facilitate equipment selection.” And could be removed for clarity.

    [Reply] Evaporation of TFA makes the selection of equipment difficult because TFA vapors can cause corrosion of equipment. It was rewritten for clarity.

19. Page 12, Line 31: there should be an “a” prior to “70% yield”. Alternatively, one could replace “to obtain” with “in”.

    [Reply] “a” was added according to the comment.

20. Page 12, Line 45: “unapplicable” should be changed to “hazardous” or some other descriptor, as unapplicable doesn’t make sense here. Does the author mean that the reagents were used with difficulty? If so, this should be changed to convey this.

    [Reply] It was rewritten for clarity.

21. Page 13, Line 6: “This described process to 1” chould be changed to “the process to 1 just described” or “the described process to 1” for smoothness.

    [Reply] It was changed according to the comment.

22. Page 17, Line 10: DIOs should not be included in references. Please change your reference formatting to match that of refences 2–8.

    [Reply] It was corrected according to the comment.

23. Page 18, Line 5: DIOs should not be included in references. Please change your reference formatting to match that of refences 2–8.

    [Reply] I have referred to the online version Electronic Encyclopedia of Reagents for Organic Synthesis (e-EROS). According to the Author guideline, the including DOI in references would be acceptable if the paper is published online.

24. At one point, the word “subsequentially” is used. This should be changed to “subsequently”

    [Reply] It was corrected according to the comment.

Response to Reviewer 2

1. Overall, this is an excellent contribution on the development of an efficient and robust process from the research team at Shionogi & Co. However, given the broad and diverse readership of ACS Central Science and the more specialized nature of this submission, my recommendation is to publish in a specialized journal, such as OPRD, after minor revisions, particularly regarding experimental aspects of the work.

   [Reply] I appreciate the review. I believe that this achievement has a significant implication for re-establishing the safety and security of society and addressing the COVID-19 pandemic, and will stimulate not only medicinal/process chemists but also the wide range of readers of *ACS Central Science*.

2. My overall impression of the experimental section in the Supporting Information is that it is too light on detail. A high-level summary of the scale up recipe is not enough and details on the process (unit operations) including temperatures, heat-cool times, feed rates, drying conditions etc. are important to ensure reproducibility. For example, the description of the ensitrelvir – fumaric acid crystallization process indicates that a solution of the API free base in acetone/water was treated with activated carbon but does not reveal how much carbon was used, how long the slurry was held, and doesn’t indicate that the carbon was even removed via filtration (I assume it was). In addition, details on other unit operations are missing e.g. how was the mixture concentrated to 36.0 volumes – distillation over how long and at what temperature / pressure? Was this a seeded crystallization? How much acetone was used to wash the product? What pressure was the product dried at? For how long? Was an agitated filter dryer used? Other? What was the melting point of the product thus obtained? Any solid form characterization to be shared? XRPD?

   Similar comments hold for all the other procedures provided – details are missing throughout (e.g. for compound 28, details of the direct drop isolation procedure including water feed rate, hold times, cooling rates, seed loading, cake washing volumes and number of washes etc are all missing)

   [Reply] In light of the comments, necessary operational conditions for reproducibility were incorporated, including operation temperature, weight of reagent and volume of solvent. Unless otherwise noted, crystallization occurred without seed loading process. Throughout the manufacturing process, the vacuum concentration was used for the concentration of the solvent. The comments were added in the Supporting Information. Moreover, solid form of API was characterized by single crystal X-Ray (also see comment/reply 7). Other process parameters were not disclosed due to the confidentiality of equipment capability. I appreciate your understanding.

The addition of operational conditions has generally been sufficient to describe the procedure. I thank the reviewer again.

3. Nitro reductions are known to proceed via the intermediacy of nitroso and hydroxyl amine species and disproportionation reactions commonly lead to dimeric byproducts including azoxy, azo, and hydrazo species which can be considered potentially mutagenic impurities (PMIs).

   How were these PMIs controlled in the process?

   [Reply] In compliance with ICH M7 guidelines, PMIs are properly assessed and controlled. I have chosen not to divulge the specific control strategy due to its confidential nature.

4. Additionally, catalytic hydrogenations of nitro compounds can also be highly exothermic – was this observed in this case and how was it controlled? Was there any accumulation of energetic species like hydroxylamine etc?

   [Reply] The rate of the heat generation during the reaction was mitigated by minimizing the quantity of Pt/C utilized. Consequently, no significant heat generation was observed with appropriate cooling equipment.

5. Were additional steps needed to eliminate residual metal in the product (metal scavengers etc)?

   [Reply] No additional manipulation was required.

6. During the synthesis of compound 30, it was noted that Cs2CO3 was superior to K2CO3 in improving the ‘reproducibility’ of the reaction. Do the authors believe this due to differences in particle size or water levels for the K2CO3 lots examined?

   [Reply] These are the important points for the reproducibility. We chose Cs_2CO_3 based on the experimental results. However, the exact reason is under the investigation.

7. The final API is described as a ensitrelvir-fumaric acid co-crystal. What evidence do the authors have that this material is obtained as a co-crystal versus a traditional fumarate salt? Is their single crystal X-Ray or NMR data or other that are consistent with a co-crystal versus salt? Physical characterization of this material should be included (mp etc)

   [Reply] I thank the reviewer for the careful review about the final API form. The analysis using single crystal X-Ray supported that the final API was obtained as co-crystal of fumaric acid. For clarity, some comments and an appropriate reference were added in the Supporting Information.

   Also, melting point was added in the Supporting Information.

Response to Reviewer 3

1. As a general comment, I would suggest that the authors revise the manuscript to highlight the novelty and challenges inherent to each of the individual transformations rather than the focusing on the ultimate conditions which were utilized. While these generally appear to be straightforward, on the scale the authors operate on, numerous process challenges are bound to have arisen. While the authors provide sufficient understanding around the advantage of the meta-cresolyl moiety, additional detail should be provided to help the reader understand how the authors arrived at that strategy. What experiments were conducted? Was the use of modelling employed?

   [Reply] I appreciate the reviewer's valuable comment. As the reviewer pointed out, many innovations and investigations were required to make each step successful. Some significant optimizations were included in the Supporting Information. In the present manuscript, we highlighted and focused on the introducing strategy of *meta*-cresolyl group. Stability experiments/subsequent substituted reactions were investigated using the compounds possessing other protecting/leaving groups alternative to *meta*-cresolyl group. As a result, it was revealed that *meta*-cresolyl group was superior to the other substituents. The comment was added in the manuscript.

2. Additionally, additional detail around the final API crystallization with fumaric acid should be provided. This is a key point which is not fully explained, both from a crystallization process and biological significance perspective.

   [Reply] As pointed out in the reviewer's comment, the selection of co-crystal as API and its significance are important. The comment was added in the manuscript.

3. One very minor comment - on S19, the word "acetone" is misspelled in a scheme.

   [Reply] It was corrected according to the comment.

oc-2022-01203u.R2

Name: Peer Review Information for "Development of a manufacturing process toward the convergent synthesis of the COVID-19 antiviral Ensitrelvir"

Second Round of Reviewer Comments

Reviewer: 3

Comments to the Author

After reading the revised manuscript, I believe it is suitable for publication in ACS Central Science without additional changes. The authors made suitable revisions that highlight the novelty of the process development central to this article. While I completely understand feedback that this is manuscript would be more suited for OPRD, I respectfully disagree with that assessment. This paper showcases the culmination of an excellent story of process development and coupled with the high value target, ACS Central Science is a suitable journal for its publication. I believe readers who are not familiar with process development will be rewarded with an informative article and thank the authors for making the changes requested.

Author's Response to Peer Review Comments:

Dear Dr. Editor,

Manuscript ID: oc-2022-01203u.R1

Title: "Development of a manufacturing process toward the convergent synthesis of the COVID-19 antiviral Ensitrelvir"

I appreciate the time and effort you and the reviewer. I have addressed the formatting issues. The revised points are listed below.

I look forward to the editorial decision.

sincerely,

Takahiro Kawajiri

Formatting Needs:

1. AU EMAIL: Please include the email address of the corresponding author on the first page of the manuscript, and the Supporting Information if submitted, with an asterisk next to their name in the author list. Please be sure to label "email."

   [Reply] It was appropriately rectified.

2. SI PARAGRAPH: If the manuscript is accompanied by any supporting information for publication, a brief description of the supplementary material is required in the manuscript. The appropriate format is: Supporting Information. Brief statement in non-sentence format listing the contents of the material supplied as Supporting Information.

   [Reply] I believe that this has already been appropriately corrected at the time of the previous point of order. Please see page 14.

3. REFS 10+ AU: References with more than 10 authors should list the first 10 authors, followed by "et al."

   [Reply] It was rectified in accordance with the comment (refs.2–9, 14, 20, 26).

[Additional change]

An appropriate citation (*ChemRxiv*., DOI: 10.26434/chemrxiv-2022-wx3nl) was newly added as Ref.39.
